# Structural Diversity in Divalent Group 14 Triflate Complexes Involving Endocyclic Thia-Macrocyclic Coordination

**DOI:** 10.1021/acs.inorgchem.2c03613

**Published:** 2023-01-05

**Authors:** Rhys P. King, Julie M. Herniman, William Levason, Gillian Reid

**Affiliations:** School of Chemistry, University of Southampton, Southampton SO17 1BJ, U.K.

## Abstract

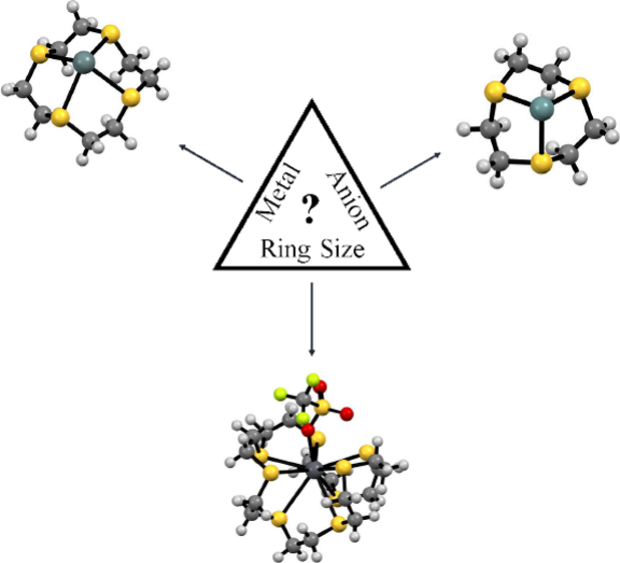

A highly unusual series of M(II) (M = Ge, Sn, Pb) complexes with endocyclic thioether macrocyclic coordination and with coordination numbers ranging from three to nine have been prepared by the reaction of [9]aneS_3_ (1,4,7-trithiacyclononane), [12]aneS_4_ (1,4,7,10-tetrathiacyclododecane), or [24]aneS_8_ (1,4,7,10,13,16,19,22-octathiacyclotetracosane) with M(OTf)_2_ (M = Sn and Pb; OTf = CF_3_SO_3_^–^) or with GeCl_2_·dioxane and 2 mol equiv of TMSOTf (Me_3_SiO_3_SCF_3_) in a mixture of anhydrous CH_2_Cl_2_ and MeCN. The isolated bulk products are characterized by ^1^H, ^13^C{^1^H}, ^19^F{^1^H}, and ^119^Sn{^1^H} NMR and IR spectroscopy, high-resolution ESI^+^ MS, and microanalytical data. Crystal structures are also reported for [M(L)][OTf]_2_ (M = Ge, Sn, Pb; L = [9]aneS_3_, [12]aneS_4_) and for [M([24]aneS_8_)][OTf]_2_ (M = Sn, Pb). In all cases, the ligand is bound in an endocyclic fashion, but the coordination environment and number are highly dependent on the group 14 ion, the macrocyclic ring size, and the number of S*-*donor atoms it presents. Solution NMR spectroscopic data suggest that the metal-macrocycle coordination is retained in solution but that the triflate anions are extensively dissociated on the NMR timescale. Density functional theory calculations on the [M([9]aneS_3_)]^2+^ and [M([12]aneS_4_)]^2+^ (M = Ge, Sn, Pb) dications reveal that the HOMO is centered on the group 14 atom as a directional “lone pair”; it also retains a significant amount of positive charge.

## Abstract 2

The preparation and characterization of a series of Ge(II), Sn(II), and Pb(II) triflate complexes with 9-, 12-, and 24-membered thia-macrocyclic ligands are described, revealing endocyclic coordination in all cases, with highly variable coordination numbers from three to nine.

## Introduction

Over the past two decades, there has been an escalation of interest in developing new coordination chemistry of the main group elements, driven by a number of different factors, including the desire for new precursors for the deposition of semiconductor materials for high tech applications,^1^ new radiopharmaceuticals for medical imaging and therapy,^2^ and metal-free catalysts,^3^ as well as intrinsic interest in broadening the types of ligands that form complexes, particularly with the lower oxidation state p-block ions. In contrast to the d-block ions where octahedral coordination predominates, p-block acceptors can display much more variable coordination numbers and geometries, with the structure, denticity, donor type(s), and steric requirements of the ligands significantly influencing the speciation in main group complexes.^4^ Moreover, while to-date much work has focused on coordination complexes of main group halides, MX_*n*_ (M = p-block acceptor; X = F, Cl, Br, I), it is well-established that the halide can influence the Lewis acidity of the p-block center very considerably, and the M–X bonding tends to be dominant, with weaker, secondary coordination to neutral Lewis base ligands. This contrast is exemplified by the coordination chemistry of thioether macrocyclic ligands with mid and late d-block halides, where substitution of the halides and endocyclic coordination of the macrocycle are typical (driven by the macrocyclic effect), whereas reaction of p-block halides from groups 14–16 with thioether macrocycles tends to produce weakly associated oligomers and polymers with retention of the MX_*n*_ fragments (primary coordination) and exocyclic (often bridging) thia-macrocyclic coordination.^5^ Swapping the halide for more weakly coordinating anions, such as triflate, fluorous tetra-arylborates, fluorous aluminates, etc., in which the negative charge is more delocalized and diffuse,^6^ can aid solubility in low polarity solvents and enable the stabilization of highly unusual and reactive species, such as the univalent [Ga(PPh_3_)_3_]^+^,^7^ as well as alkali metal cation complexes with homoleptic soft phosphine or thioether coordination.^8^ However, systematic studies on p-block acceptors, beyond the halides, are rare.

Within group 14 (Si–Pb), both the +2 and + 4 oxidation states are accessible, with the +2 oxidation state becoming more common as the group is descended.^9^ This means that for silicon, the coordination chemistry is almost exclusively based upon silicon(IV), with only a very small number of molecular complexes containing silicon(II) (typically with strong σ-donating and sterically demanding N-heterocyclic carbenes).^10^ For germanium, recent reports have described a range of molecular germanium(II) species, typically with multidentate or macrocyclic ligands (vide infra). While for tin, complexes of both the +4 and +2 oxidation states are common, the coordination chemistry of lead with neutral Lewis bases is dominated by the +2 oxidation state.

While many complexes of divalent group 14 species with neutral ligands involve coordination to the dihalides, often forming neutral complexes with the halides retained,^5^ cationic and dicationic complexes of the heavy group 14 elements have been isolated with a variety of ligands. These include N- and O-donors, for example, cryptands,^11^ aza-macrocycles,^12^ crown ethers,^12,13^ imines,^14^ imidazolyl-based chelates,^15^ as well as with C-donor ligands (e.g., N-heterocyclic carbenes^16^ and isocyanides^17^). Discrete dications of germanium with homoleptic soft phosphine and arsine donor sets have also been reported very recently, including [Ge(PMe_3_)_3_]^2+^ and [Ge(triars)]^2+^ (triars = MeC(CH_2_AsMe_2_)_3_).^18^ An important factor for the isolation of the dications was to use a weakly coordinating anion (in this case OTf) to decrease the likelihood that the anion would compete with the neutral pnictine for coordination. In contrast, there are no reported examples of dications of germanium(II) with weaker thioether or selenoether donor ligands. A small number of tin(II) and lead(II) dications with phosphine ligands have been known for a long time, although they have only been detected in solution in multinuclear NMR studies.^19^ With group 16 donor ligands. While there are many examples of lead(II) complexes with mixed S,O- and S,N-donor macrocycles, only three structurally characterized examples are known with S-donor only macrocycles, [Pb([10]aneS_3_)(H_2_O)][ClO_4_]_2_ ([10]aneS_3_ = 1,4,7-trithiacyclodecane), [Pb_2_([28]aneS_8_)][ClO_4_]_4_ ([28]aneS_8_ = 1,4,8,11,14,18,21,25-octathiacyclooctacosane), and [Pb([9]aneS_3_)_2_][ClO_4_]_2_.^20^ To the best of our knowledge, there are no structurally characterized complexes of tin(II) with S-donor-only macrocycles, although there are a number with mixed S,O-donor sets, for example, [Sn([18]aneS_2_O_4_)(H_2_O)_2_][PF_6_][F] and [Sn([18]aneS_2_O_4_)(HO(CH_2_)_2_OH)][BF_4_]_2_ ([18]aneS_2_O_4_ = 1,10-dithia-4,7,13,16-tetraoxacyclo-octadecane).^21^ Each of these lead(II) and tin(II) complexes has the macrocycle coordinated in an endocyclic fashion. For germanium(II), there are three structurally characterized complexes with mixed S,O-donor macrocycles, [GeCl([18]aneS_3_O_3_)][X] (X = GeCl_3_^–^ or OTf^–^; [18]aneS_3_O_3_ = 1,3,7-trithia-10,13,16-trioxacyclooctadecane) and [GeCl([15]aneS_2_O_3_][GeCl_3_] ([15]aneS_2_O_3_ = 1,4-dithia-7,10,13-trioxacyclopentadecane), all based on endocyclic coordination to the germanium(II) and forming monocations.^22^ With thioether-only macrocycles, a small number of examples with germanium(II) halides, GeX_2_(thia-macrocycle), are known. However, without exception, the macrocycle binds in an exocyclic manner, bridging between GeX_2_ units to form either 2D sheets or 1D chain polymers.^23^

Here, we report a systematic study of the coordination chemistry of divalent group 14 triflates (Ge, Sn, Pb) with three neutral thioether macrocycles, [9]aneS_3_, [12]aneS_4_, and [24]aneS_8_, which incorporate different numbers of S-donor atoms and different ring sizes (and therefore binding cavities). The molecular structures of eight of the resulting (monometallic) complexes have been determined via X-ray crystallography, and density functional theory (DFT) calculations have been used to probe their electronic structures and charge distributions.

## Experimental Section

GeCl_2_·dioxane, Sn(OTf)_2_, Pb(OTf)_2_, [9]aneS_3_, [12]aneS_4_, and [24]aneS_8_ were obtained from Sigma-Aldrich. The metal triflates were dried by gentle heating in vacuo for 2–3 h prior to use. TMSOTf (Sigma-Aldrich) was distilled prior to use. All reactions were conducted using Schlenk, vacuum line, and glovebox techniques and under a dry dinitrogen atmosphere. CH_2_Cl_2_ and MeCN were dried by distillation from CaH_2_ and *n*-hexane from Na and stored over activated molecular sieves. NMR solvents were also stored over 4 Å sieves.

IR spectra were recorded as Nujol mulls between CsI plates using a PerkinElmer Spectrum 100 spectrometer over the range of 4000–200 cm^–1^. NMR spectra were recorded using a Bruker AVII 400 or AVIII HD400 spectrometer. ^1^H and ^13^C{^1^H} NMR spectra were referenced to residual solvent resonances, ^19^F{^1^H} NMR spectra to external CFCl_3_, and ^119^Sn{^1^H} NMR spectra to SnMe_4_. Microanalytical measurements were performed by Medac Ltd. For ESI^+^, mass spectrometry samples were diluted into acetonitrile to an approximate concentration of 10 μg/mL. The solution was infused using a syringe driver at a constant flow rate of 3 μL/min. High-resolution positive ion electrospray mass spectra were recorded using a MaXis (Bruker Daltonics, Bremen, Germany) time of flight mass spectrometer. Data were processed using Bruker Compass DataAnalysis software 1.3.

### X-ray Crystallography

Single crystals were grown as described in the Results and Discussion section. Single-crystal X-ray data were collected using a Rigaku AFC12 goniometer equipped with an enhanced sensitivity (HG) Saturn724+ detector mounted at the window of an FR-E+ SuperBright molybdenum (λ = 0.71073 Å) rotating anode generator with VHF or HF Varimax optics (70 or 10 μm focus), with the crystal held at 100 K (N_2_ cryostream). Structure refinements were performed with either SHELX(S/L)97 or SHELX(S/L)2013 through Olex225^24^ and were mostly straightforward, with H atoms bonding to C atoms placed in calculated positions using default C–H distances. Where additional constraints or restraints were required, details are provided in the cif file for each structure. For [Sn([24]aneS_8_)(OTf)][OTf], there are two complexes in the asymmetric unit, one of which shows disorder in the CH_2_ units within the macrocyclic ring, which has been modeled satisfactorily by using a split (1:1 ratio) C atom occupancy; for the disordered C atoms, the associated H atoms were not located. For [Pb([24]aneS_8_)(OTf)][OTf], the bound triflate is disordered over two sites in a 0.88:0.12 ratio. For [Pb([12]aneS_4_)][OTf]_2_, the whole macrocycle is disordered over two sites which have been modeled with split occupancies (1:1 ratio); H atoms have not been located in this case. CCDC reference numbers for the crystallographic information files in the cif format are as follows: [Ge([9]aneS_3_)][OTf]_2_ (**1**) (2209364), [Ge([12]aneS_4_)][OTf]_2_ (**2**) (2209363), [Sn([9]aneS_3_)][OTf]_2_ (**4**) (2209365), [Sn([12]aneS_4_)][OTf]_2_ (**5**) (2209366), [Ge([9]aneS_3_)][OTf]_2_·MeCN (**1**·MeCN) (2209367), [Pb([9]aneS_3_)][OTf]_2_ (**7**) (2209368), [Pb([12]aneS_4_)][OTf]_2_ (**8**) (2209369), [Sn([24]aneS_8_)][OTf]_2_ (**6**) (2209370), [Pb([24]aneS_8_)(OTf)][OTf] (**9**) (2209371).

### Complex Syntheses

#### [Ge([9]aneS_3_)][OTf]_2_ (**1**)

GeCl_2_·dioxane (0.200 g, 0.864 mmol) was suspended in CH_2_Cl_2_ (2 mL), and to this were added [9]aneS_3_ (0.156 g, 0.864 mmol) and a solution of TMSOTf (0.384 g, 1.73 mmol) in CH_2_Cl_2_ (2 mL). The resulting clear, colorless solution was stirred for 2 h. Volatiles were removed in vacuo to leave a white solid, which was washed with hexane (3 × 10 mL) and dried in vacuo. Yield: 0.302 g, 63%. Required for C_8_H_12_F_6_GeO_6_S_5_ (551.08): C, 17.43; H, 2.2. Found: C, 17.18; H, 2.5%. ^1^H NMR (CD_3_CN, 298 K): δ = 3.66 (br s). ^13^C{^1^H} NMR (CD_3_CN, 298 K): δ = 38.6 (s). ^19^F{^1^H} NMR (CD_3_CN, 298 K): δ = −79.3 (s, OTf). IR (Nujol/cm^–1^): 1137 (OSO_2_), 1221, 1261 (CF_3_). HRMS (ESI^+^, MeCN): *m*/*z* calculated for [Ge([9]aneS_3_)(OTf)]^+^ = 402.8825, found 402.8825; *m*/*z* calculated for [Ge([9]aneS_3_)]^2+^ = 126.9650, found 126.9648.

#### [Ge([12]aneS_4_)][OTf]_2_ (**2**)

The compound 2 was prepared according to the same method as above using GeCl_2_·dioxane (0.072 g, 0.312 mmol), TMSOTf (0.139 g, 0.625 mmol), and [12]aneS_4_ (0.075 g, 0.312 mmol). White solid. Yield: 0.101 g, 63%. Required for C_10_H_16_F_6_GeO_6_S_6_·CH_2_Cl_2_ (696.11): C, 18.98; H, 2.6. Found: C, 18.97; H, 3.0%. ^1^H NMR (CD_3_CN, 298 K): δ = 3.42 (br s). ^13^C{^1^H} NMR (CD_3_CN, 298 K): δ = 35.2 (br s). ^19^F{^1^H} NMR (CD_3_CN, 298 K): δ = −79.4 (s, OTf). IR (Nujol/cm^–1^): 1141 (OSO_2_), 1222, 1264 (CF_3_). HRMS (ESI^+^, MeCN): *m*/*z* calculated for [Ge([12]aneS_4_)(OTf)]^+^ = 462.8857, found 462.8861; *m*/*z* calculated for [Ge([12]aneS_4_)]^2+^ = 156.9666, found 156.9673.

#### [Ge([24]aneS_8_)][OTf]_2_ (**3**)

The compound **3** was prepared according to the same method as above using GeCl_2_·dioxane (0.012 g, 0.052 mmol), TMSOTf (0.023 g, 0.104 mmol), and [24]aneS_8_ (0.025 g, 0.052 mmol), with MeCN (1 mL), forming a colorless solution which was stirred for 1 h. Removal of the volatiles in vacuo afforded a colorless solid. Yield: 0.024 g, 55%. Required for C_18_H_32_F_6_GeO_6_S_10_ (851.63): C, 25.4; H, 3.8. Found: C, 25.1; H, 4.3. ^1^H NMR (CD_2_Cl_2_, 298 K): δ = 3.11 (br s). ^13^C{^1^H} NMR (CD_2_Cl_2_, 298 K): δ = 33.9 (s). ^19^F{^1^H} NMR (CH_2_Cl_2_, 298 K): δ = −78.7 (s, OTf). IR (Nujol/cm^–1^): 1172 (OSO_2_), 1264 (CF_3_). HRMS (ESI^+^, MeCN): *m*/*z* calculated for [Ge([24]aneS_8_)(OTf)]^+^ = 702.8991, found 702.8992.

#### [Sn([9]aneS_3_)][OTf]_2_ (**4**)

Sn(OTf)_2_ (0.200 g, 0.480 mmol) was suspended in CH_2_Cl_2_ (2 mL), and to this was added [9]aneS_3_ (0.086 g, 0.477 mmol), producing a cloudy white mixture. MeCN (5 mL) was then added, causing dissolution of the solids to give a clear, colorless solution. The reaction mixture was stirred for 1 h, volatiles were removed in vacuo, and the resultant solid was washed with hexane (3 × 10 mL) before drying in vacuo. Yield: 0.170 g, 59%. Required for C_8_H_12_F_6_O_6_S_5_Sn (551.08): C, 16.1; H, 2.0. Found: C, 16.6; H, 2.4%. ^1^H NMR (CD_3_CN, 298 K): δ = 3.42 (s). ^13^C{^1^H} NMR (CD_3_CN, 298 K): δ = 34.3 (s). ^19^F{^1^H} NMR (CD_3_CN, 298 K): δ = −79.4 (s, OTf). ^119^Sn{^1^H} NMR (CD_3_CN, 298 K): δ = −737 (s). IR (Nujol/cm^–1^): 1151 (OSO_2_), 1229, 1260 (CF_3_). HRMS (ESI^+^, MeCN): *m*/*z* calculated for [Sn([9]aneS_3_)(OTf)]^+^ = 448.8633, found 448.8639; *m*/*z* calculated for [Sn([9]aneS_3_)]^2+^ = 149.9554, found 149.9555.

#### [Sn([12]aneS_4_)][OTf]_2_ (**5**)

A Schlenk flask was charged with Sn(OTf)_2_ (0.087 g, 0.209 mmol) and [12]aneS_4_ (0.050 g, 2.08 mmol), together with CH_2_Cl_2_ (2 mL) and MeCN (4 mL), forming a slightly cloudy solution. The mixture was stirred for 2 h and filtered to remove particulates. The volatiles were then removed in vacuo, yielding a white solid which was washed in hexane and dried in vacuo. Yield: 0.109 g, 79%. Required for C_10_H_16_F_6_O_6_S_6_Sn (657.27): C, 18.3; H, 2.5. Found: C, 18.2; H, 2.9%. ^1^H NMR (CD_3_CN, 298 K): δ = 3.29 (s). ^13^C{^1^H} NMR (CD_3_CN, 298 K): δ = 33.3 (s). ^19^F{^1^H} NMR (CD_3_CN, 298 K): δ = −79.3 (s, OTf). ^119^Sn{^1^H} NMR (CD_3_CN, 298 K): δ = −903 (s). IR (Nujol/cm^–1^): 1158 (OSO_2_), 1230, 1261 (CF_3_). MS (ESI^+^, MeCN): *m*/*z* calculated for [Sn([12]aneS_4_)(OTf)]^+^ = 508.8666; found 508.8699, *m*/*z* calculated for [Sn([12]aneS_4_)]^2+^ = 223.9943; found 223.9948.

#### [Sn([24]aneS_8_)][OTf]_2_ (**6**)

Sn(OTf)_2_ (0.022 g, 0.053 mmol) was suspended in CH_2_Cl_2_ (2 mL), and [24]aneS_8_ (0.025 g, 0.052 mol) was added as a solution in CH_2_Cl_2_ (1 mL), along with MeCN (3 mL), forming a colorless solution which was stirred for 1 h. The solvent was then removed in vacuo to yield a colorless solid that was washed with *n*-hexane and dried in vacuo. Yield: 0.027 g, 68%. Required for C_18_H_32_F_6_O_6_S_10_Sn (897.71): C, 24.1; H, 3.6. Found: C, 24.3; H, 4.1%. ^1^H NMR (CD_2_Cl_2_, 298 K): δ = 3.10 (br s). ^13^C{^1^H} NMR (CD_2_Cl_2_, 298 K): δ = 33.6 (s). ^19^F{^1^H} NMR (CH_2_Cl_2_, 298 K): δ = −78.4 (s, OTf). ^119^Sn{^1^H} NMR (CD_2_Cl_2_, 298 K): δ = −1079 (s). IR (Nujol/cm^–1^): 1231 (OSO_2_), 1228, 1261 (CF_3_). HRMS (ESI^+^, MeCN): *m*/*z* calculated for [Sn([24]aneS_8_)(OTf)]^+^ = 748.8797, found 748.8813; *m*/*z* calculated for [Sn([24]aneS_8_)]^2+^ = 299.9636, found 299.9642.

#### [Pb([9]aneS_3_)][OTf]_2_ (**7**)

Pb(OTf)_2_ (0.200 g, 0.396 mmol) was suspended in CH_2_Cl_2_ (2 mL), and [9]aneS_3_ (0.072 g, 0.399 mmol) was added, yielding a cloudy yellow mixture. Addition of MeCN (3 mL) caused dissolution of the insoluble material to yield a clear yellow solution, which was stirred for 1 h. Volatiles were removed in vacuo, and the resultant white solid was washed with hexane (3 × 10 mL) and dried in vacuo. Yield: 0.141 g, 53%. Required for C_8_H_12_F_6_O_6_PbS_5_·1/4C_6_H_14_ (707.24): C, 16.1; H, 2.2. Found: C, 16.1; H, 2.4%. ^1^H NMR (CD_3_CN, 298 K): δ = 3.63 (s). ^13^C{^1^H} NMR (CD_3_CN, 298 K): δ = 33.6 (s). ^19^F{^1^H} NMR (CD_3_CN, 298 K): δ = −79.3 (s, OTf). IR (Nujol/cm^–1^): 1154 (OSO_2_), 1228, 1261 (CF_3_). HRMS (ESI^+^, MeCN): *m*/*z* calculated for [Pb([9]aneS_3_)(OTf)]^+^ = 536.9378, found 536.9390; *m*/*z* calculated for [Pb([9]aneS_3_)]^2+^ = 193.9927, found 193.9930.

#### [Pb([12]aneS_4_)][OTf]_2_ (**8**)

A Schlenk flask was charged with Pb(OTf)_2_ (0.105 g, 0.208 mmol) and [12]aneS_4_ (0.050 g, 0.208 mmol), and to this were added CH_2_Cl_2_ (2 mL) and MeCN (4 mL), forming a slightly cloudy solution. The reaction mixture was stirred for 2 h, and the resulting white precipitate was collected by filtration, washed with hexane (3 × 10 mL), and dried in vacuo. Yield: 0.117 g, 75%. Required for C_10_H_16_F_6_O_6_PbS_6_·0.25MeCN (756.03): C, 16.7; H, 2.2; N, 0.5. Found: C, 16.4; H, 2.5; N, 0.4%. ^1^H NMR (CD_3_CN, 298 K): δ = 3.38 (s). ^13^C{^1^H} NMR (CD_3_CN, 298 K): δ = 31.9 (s). ^19^F{^1^H} NMR (CD_3_CN, 298 K): δ = −79.3 (s, OTf). IR (Nujol/cm^–1^): 1169 (OSO_2_), 1221, 1285 (CF_3_). HRMS (ESI^+^, MeCN): *m*/*z* calculated for [Pb([12]aneS_4_)(OTf)]^+^ = 596.9411, found 596.9423; *m*/*z* calculated for [Pb([12]aneS_4_)]^2+^ = 223.9943, found 223.9948.

#### [Pb([24]aneS_8_)][OTf]_2_ (**9**)

Pb(OTf)_2_ (0.026 g, 0.051 mmol) was suspended in CH_2_Cl_2_ (2 mL), and [24]aneS_8_ (0.025 g, 0.052 mmol) was added as a solution in CH_2_Cl_2_ (1 mL), followed by MeCN (3 mL). This gave a colorless solution which was stirred for 1 h. The solvent was removed in vacuo to yield a white solid which was washed with hexane and dried in vacuo. Yield: 0.021 g, 41%. Required for C_18_H_32_F_6_O_6_PbS_10_·0.5C_6_H_14_ (1029.29): C, 24.5; H, 3.8. Found: C, 24.8; H, 3.9%. ^1^H NMR (CD_2_Cl_2_, 298 K): δ = 3.15 (br s). ^13^C{^1^H} NMR (CD_2_Cl_2_, 298 K): δ = 33.5 (s). ^19^F{^1^H} NMR (CH_2_Cl_2_, 298 K): δ = −78.7 (s, OTf). HRMS (ESI^+^, MeCN): *m*/*z* calculated for [Pb([24]aneS_8_)(OTf)]^+^ = 836.9543, found 836.9549; *m*/*z* calculated for [Pb([24]aneS_8_)]^2+^ = 344.0009, found 344.0011.

##### DFT Computational Details

The electronic structures of [M([9]aneS_3_)]^2+^ (M = Ge, Sn, Pb), [M([12]aneS_4_)]^2+^ (M = Ge, Sn, Pb), [Pb([24]aneS_8_)(OTf)]^+^, and [Pb([24]aneS_8_)]^2+^ were investigated using DFT calculations using the Gaussian 16W software package.^25^ The density functional chosen was B3LYP-D3^26^ with the 6-311G(d) basis set^27^ for the H, C, O, F, S, and Ge atoms, while for the Sn and Pb atoms, the LANL2DZ basis set was used.^28^ The initial geometries were taken from their crystal structures for geometry optimization calculations. In all cases, the structures converged to a stable geometry with no imaginary frequencies. The DFT-determined geometries were in good agreement with the crystallographic geometries (Supporting Information, Table S2).

## Results and Discussion

### Preparation of M(II) Complexes (M = Ge, Sn, Pb)

**Scheme 1:**
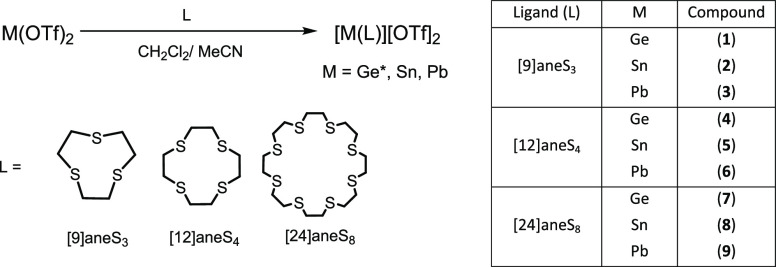
Synthesis Routes to the Thia-Macrocyclic Complexes with the Divalent Group 14 Triflates in This Work and Compound Numbers (*Ge(OTf)_2_ Was Prepared In Situ from the Reaction of GeCl_2_(dioxane) with 2 equiv of TMSOTf as Described, for Example, in Reference (15))

The germanium(II) complexes [Ge(L)][OTf]_2_ (**1–3**) were synthesized by reacting GeCl_2_·dioxane with 2 equiv of TMSOTf, followed by 1 equiv of the macrocycle, L (L = [9]aneS_3_, [12]aneS_4_, or [24]aneS_8_), in a mixture of CH_2_Cl_2_ and MeCN. The related complexes [M(L)][OTf]_2_ (M = Sn, Pb; (**4–9**)) were synthesized by the direct reaction of the ligand L with the metal triflate, M(OTf)_2_, in a mixture of CH_2_Cl_2_ and MeCN (Scheme 1). In all cases, the complexes were isolated as white powdered solids in moderate to good yields. The purity of the bulk products was determined by elemental analysis, and positive ion electrospray MS data (MeCN) showed clusters of peaks with the correct isotopic distribution corresponding to both [M(macrocycle)(OTf)]^+^ and [M(macrocycle)]^2+^ for each complex. ^1^H and ^13^C{^1^H} NMR analysis shows significant high-frequency shifts cf. the macrocycles themselves, suggesting retention of the coordinated macrocycle in the solution, albeit with some dynamic process such as “ring-whizzing” causing equivalence of the H and C environments in each case.

#### [9]aneS_3_ Complexes of Ge(II), Sn(II), and Pb(II)

**Figure 1:**
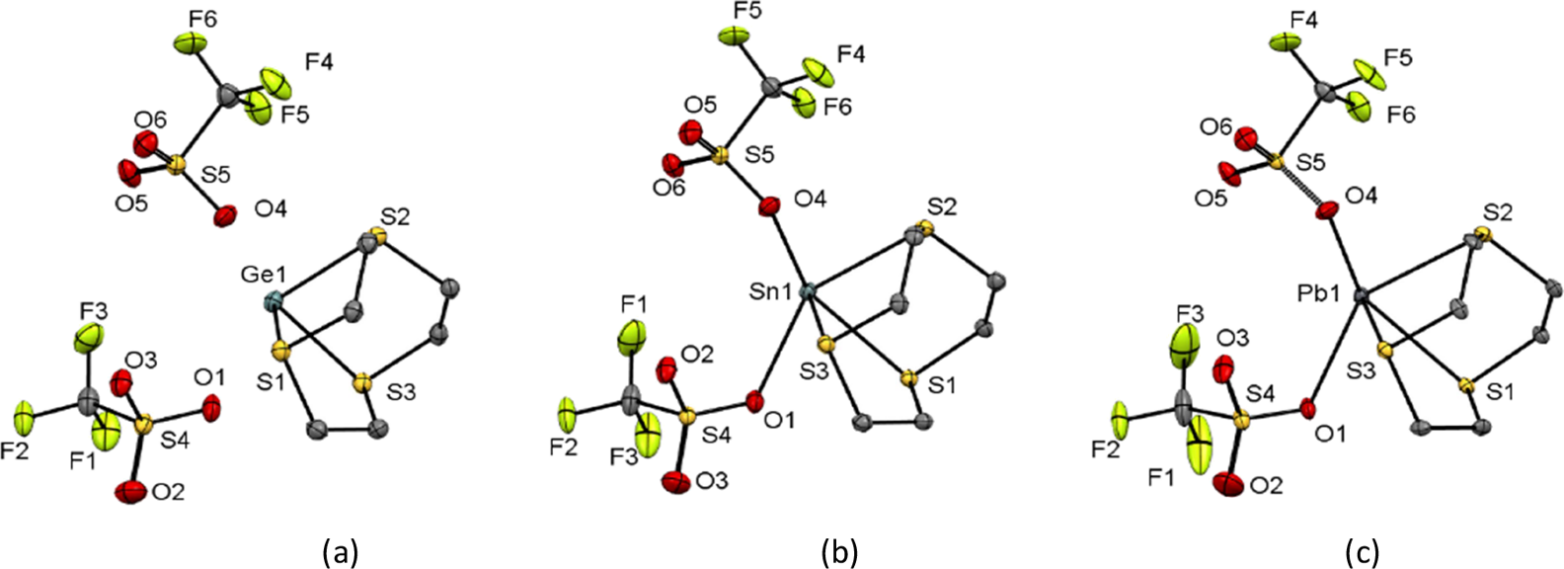
Structures of (a) [Ge([9]aneS_3_)][OTf]_2_ (**1**), (b) [Sn([9]aneS_3_)][OTf]_2_ (**4**), (c) [Pb([9]aneS_3_)][OTf]_2_ (**7**), showing the atom-labeling scheme. The ellipsoids are drawn at the 50% probability level, and H atoms are omitted for clarity.

**Figure 2:**
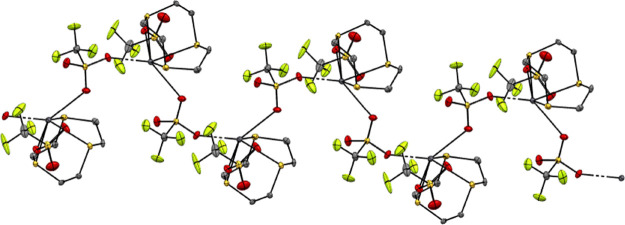
View of the extended structure of [Pb([9]aneS_3_)][OTf]_2_ (**7**) showing the 1-D polymeric structure.

Crystals of [M([9]aneS_3_)][OTf]_2_, complexes (**1**), (**4**), and (**7**), were grown from either layering a CH_2_Cl_2_ solution of the compound with hexane (M = Ge) or layering a MeCN/CH_2_Cl_2_ solution with hexane (M = Ge, Sn, Pb); the structures of all three complexes are shown in Figure 1. The complexes, [M([9]aneS_3_][OTf]_2_, are isostructural, each crystallizing in the *P*2_1_/*c* space group. The structure of an MeCN solvate, [Ge([9]aneS_3_)][OTf]_2_·MeCN (**1**·MeCN), was also obtained from recrystallization of the germanium complex in the presence of acetonitrile. They all contain one [9]aneS_3_ ligand coordinated to the group 14 center in an endocyclic κ^3^-fashion, creating a pyramidal coordination environment at M. In the extended structures, long contacts occur between Pb and one O atom from three distinct triflate anions to complete the coordination environment at the metal, with triflate groups bridging between [Pb([9]aneS_3_)]^2+^ units to form a weakly associated 1D polymer (Figure 2).

In the related complex, [GeCl_2_([9]aneS_3_)],^23^ the [9]aneS_3_ ligand binds in an exocyclic κ^1^-coordination mode and bridges GeCl_2_ centers. Replacing the halide for the more weakly coordinating triflate strengthens the Ge–S interactions and promotes endocyclic coordination to germanium(II); this is reflected in the Ge–S bond lengths with the distances being markedly shorter in the triflate case (2.5077(6), 2.4785(7), and 2.5072(6) Å vs 2.721(3) and 2.741(3) Å). This effect is also seen in the phosphine complexes of germanium(II) with halide and triflate counter anions.^18^ This facial coordination of [9]aneS_3_ to germanium(II) is broadly similar to the structure reported for the related nine-membered triaza-macrocyclic complex, [Ge(Me_3_[9]aneN_3_)][OTf]_2_ (Me_3_[9]aneN_3_ = 1,4,7-trimethyl-1,4,7-triazacyclononane), albeit the Ge–N bonds are shorter (2.084(2)–2.106(2) Å) than the Ge–S bonds in the sulfur analogue, reflecting the relative size of S versus N. In the Me_3_[9]aneN_3_ species, there are three triflates located adjacent to the dication, with Ge···O distances (∼2.85–3.39 Å), i.e., longer and weaker contacts than in the trithia system. This may be due to the face of the germanium atom being partially blocked by the Me groups preventing closer approach of the triflate anions in the Me_3_[9]aneN_3_ complex.^29^ There are no structurally authenticated tin(II) complexes with [9]aneS_3_ in the literature with which to compare; however, the tin(IV) complex, [SnCl_3_([9]aneS_3_)]_2_[SnCl_6_],^30^ involves κ^3^-coordination and, as expected, the Sn–S bond lengths are slightly shorter than in the tin(II) complex reported here. The only structurally characterized complex of [9]aneS_3_ with lead(II) is the sandwich complex, [Pb([9]aneS_3_)_2_][ClO_4_]_2_.^20a^ In this case, both macrocycles are κ^3^-coordinated, and the two O-bound ClO_4_^–^ anions lead to a coordination number at lead of eight, contrasting with the coordination of just one trithia-macrocycle to the lead center in the triflate species reported here. The Pb–S bond distances are also significantly shorter in [Pb([9]aneS_3_)][OTf]_2_ compared to those in [Pb([9]aneS_3_)_2_][ClO_4_]_2_ (2.7831(9)–2.8622(8) vs 3.083–3.139 Å, respectively), reflecting the lower coordination number in the former.

Comparing the three [M([9]aneS_3_))][OTf]_2_ (M = Ge, Sn, Pb) complexes, the M–S bond distances increase as the group 14 element gets heavier (Table 1), consistent with the increase in the ionic radii. Further, due to the constraints of the nine-membered macrocyclic ring, this increase in *d*(M–S) causes a significant decrease in the S–M–S bond angles, from ca. 83° for Ge to ca. 77° for Sn and ca. 75° for Pb. This also causes a marked increase in the distance from the centroid of the S_3_ plane (centroid defined by S1–S2–S3) to the metal, reflecting a mismatch between the nine-membered trithia-macrocycle and the larger metal ions.

**Table 1:** Selected Crystallographically Determined Geometric Parameters for [M([9]aneS_3_)][OTf]_2_ (M = Ge, Sn, Pb), with esds in Parentheses

[M([9]aneS_3_)][OTf]_2_	M = Ge (**1**)	M = Sn (**4**)	M = Pb (**7**)
*d*(M–S)/Å	2.4854(7)	2.7301(5)	2.7831(9)
	2.5080(7)	2.6996(6)	2.8279(10)
	2.5450(7)	2.7789(6)	2.8622(8)
*d*(M···O)/Å	2.6949(19)	2.5479(18)	2.732(3)
	2.5784(19)	2.7352(19)	2.610(2)
	2.8286(19)	2.819(2)	2.782(3)
∠(S–M–S)/deg	84.38(2)	76.409(17)	74.16(3)
	83.76(2)	78.250(18)	76.20(2)
	82.77(2)	77.905(17)	76.13(3)
*d*(centroid S1–S2–S3 to M)/Å	1.604	1.891	1.996

#### [12]aneS_4_ Complexes of Ge(II), Sn(II), and Pb(II)

**Figure 3:**
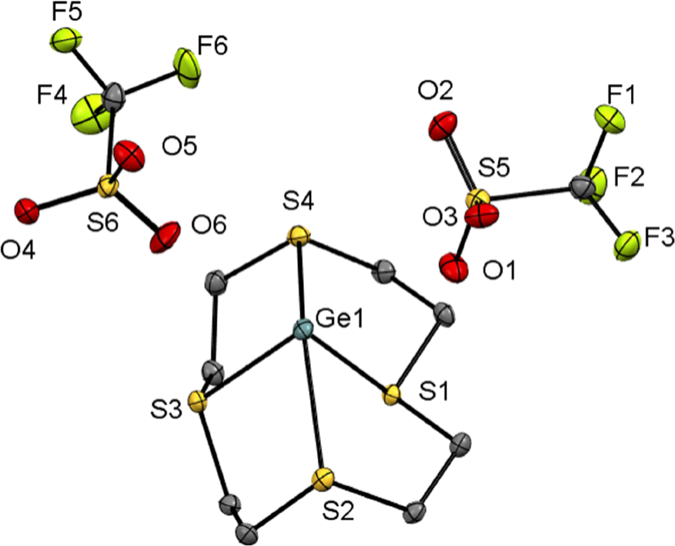
Structure of [Ge([12]aneS_4_)][OTf]_2_ (**2**) showing the atom-labeling scheme. Ellipsoids are drawn at the 50% probability level, and H atoms are omitted for clarity. Selected bond lengths (Å) and angles (deg) are as follows: Ge1–S1 = 2.4446(4), Ge1–S2 = 2.7338(5), Ge1–S3 = 2.5050(4), Ge1–S4 = 2.7717(5), Ge1···O1 = 2.8018(14), Ge1···O6 = 2.8002(14), S1–Ge1–S2 = 75.098(14), S2–Ge1–S3 = 79.714(14), S3–Ge1–S4 = 80.724(14), S4–Ge1–S1 = 74.685(14), S1–Ge1–S3 = 83.465(14), S2–Ge1–S4 = 145.525(14).

The coordination of the divalent group 14 triflates was also explored with the larger, tetrathia macrocycle, [12]aneS_4_, to establish whether endocyclic coordination would also prevail. The complexes [M([12]aneS_4_)][OTf]_2_ were synthesized as in Scheme 1 and were all crystallized by layering a MeCN/CH_2_Cl_2_ solution of the complex with hexane. The crystal structures show that in [Ge([12]aneS_4_)][OTf]_2_ (**2**), the thia-macrocycle does indeed bind in an endocyclic κ^4^-coordination mode (Figure 3) with two long Ge–S bonds (2.7338(5) and 2.7717(5) Å) and two short bonds (2.5050(4) and 2.4446(4) Å); the sulfur atoms are bound in a *syn*,*syn*,*syn*,*anti* fashion (relative orientations of the S-based lone pairs). The closest Ge···O contacts from two nearby triflates are 2.8018(14) and 2.8002(14) Å; however, the S–O bonds lengths within each triflate are not statistically different, suggesting that an ion-separated (dication and discrete anions) description is more appropriate.

The tetraaza-macrocyclic Ge(II) dications, [Ge(Me_4_[12]aneN_4_)]^2+^^27^ and [Ge(Me_4_[14]aneN_4_)]^2+^^12^ (Me_4_[12]aneN_4_ = 1,4,7,10-tetramethyl-1,4,7,10-tetraazacyclododecane; Me_4_[14]aneN_4_ = 1,4,8,11-tetramethyl-1,4,8,11-tetraazacyclotetradecane), also have the macrocycle coordinating in an endocyclic κ^4^-fashion, with the Me_4_[14]aneN_4_ complex having a similar pattern of two short and two long Ge–N bonds as observed for the Ge–S bonds in [Ge([12]aneS_4_)][OTf]_2_ reported here, whereas in the smaller ring Me_4_[12]aneN_4_ complex, all the Ge–N bonds are of a similar length. It is likely that the smaller effective binding cavity for the 12-membered aza-macrocycle, by virtue of the C–N bonds being shorter than the C–S bonds in the corresponding tetrathioether, leads to more optimal Ge–N bonds in the former. The 12-membered crown ether reacts with Ge(II) to form the dicationic [Ge(12-crown-4)_2_]^2+^ ion in which the eight-coordinate germanium(II) center is sandwiched between two crown ethers.^12,13^

The endocyclic structure observed here for [Ge([12]aneS_4_)][OTf]_2_ (**2**) also contrasts with the structures reported previously for both [GeCl_2_([14]aneS_4_)] and [GeBr_2_([16]aneS_4_)] ([14]aneS_4_ = 1,4,8,11-tetrathiacyclotetradecane; [16]aneS_4_ = 1,5,9,13-tetrathiacyclohexadecane), which have the macrocycle in an exocyclic coordination mode, bridging GeX_2_ units and κ^1^-bound to each Ge center. The former adopts a 2D sheet structure and the latter a 1D polymer. Comparing the Ge–S bond lengths in these species, the two shortest bond lengths in [Ge([12]aneS_4_)][OTf]_2_ are about 0.3 Å shorter than *d*(Ge–S) in each of the GeX_2_ polymers, while the two longer Ge–S bonds are more comparable to *d*(Ge–S) in the GeX_2_ complexes. This is consistent with the pattern found in the [9]aneS_3_ complexes (vide supra), again demonstrating the increased Lewis acidity of germanium(II) center in the OTf species compared to the halide complexes.^22^

**Figure 4:**
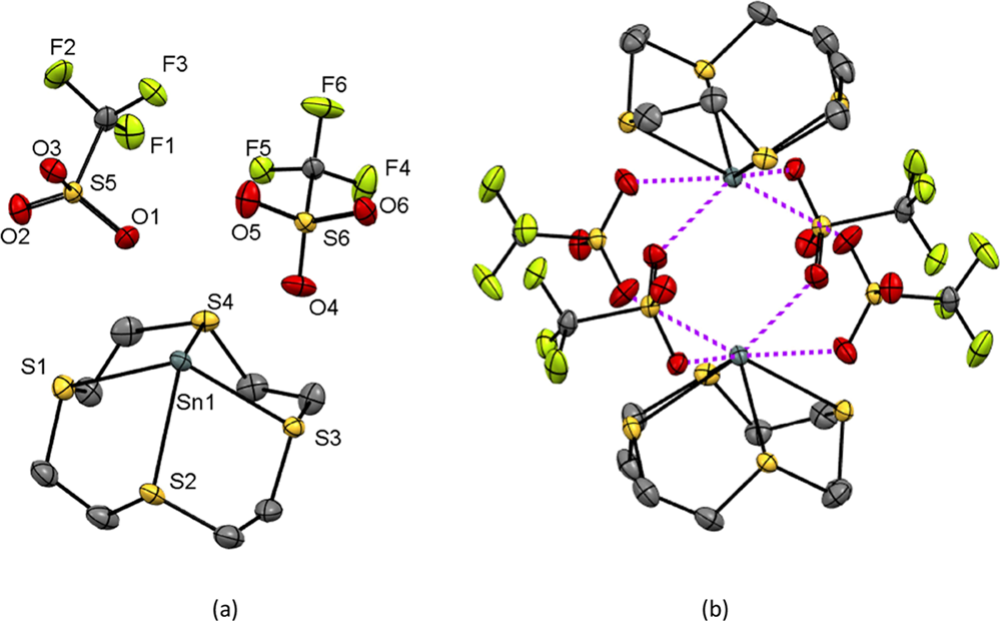
(a) Structure of [Sn([12]aneS_4_)][OTf]_2_ (**5**) showing the atom-labeling scheme. Ellipsoids are drawn at the 50% probability level, and H atoms are omitted for clarity. Selected bond lengths (Å) and angles (deg) are as follows: Sn1–S1 = 2.9053(9), Sn1–S2 = 2.8176(8), Sn1–S3 = 2.8160(8), Sn1–S4 = 2.8675(8), Sn1···O1 = 2.887(2), Sn1···O3 = 3.024(3), Sn1···O4 = 2.876(3), Sn1···O5 = 2.902(3), S1–Sn1–S2 = 72.30(3), S2–Sn1–S3 = 73.41(2), S3–Sn1–S4 = 72.41(2), S4–Sn1–S1 = 72.65(3), S1–Sn1–S3 = 115.72(2), S2–Sn1–S4 = 113.91(3); (b) weakly associated dimeric structure of [Sn([12]aneS_4_)][OTf]_2_ in the solid state.

For [Sn([12]aneS_4_)][OTf]_2_ (**5**) (Figure 4a), the macrocycle is also bound in an endocyclic κ^4^-coordination mode, but in this case, the S atoms are bound in an all-*syn* fashion (i.e., the lone pairs on all four S atoms are mutually *syn*) and the Sn–S bond distances fall within a narrow range (2.8160(8)–2.9053(9) Å). There are further long contacts to four weakly bridging triflates (*d*(Sn···O) = 2.876(3)–3.024(3) Å), giving rise to a weakly associated dimer structure (Figure 4b), with an eight-coordinate distorted square-antiprismatic geometry at tin(II).

**Figure 5:**
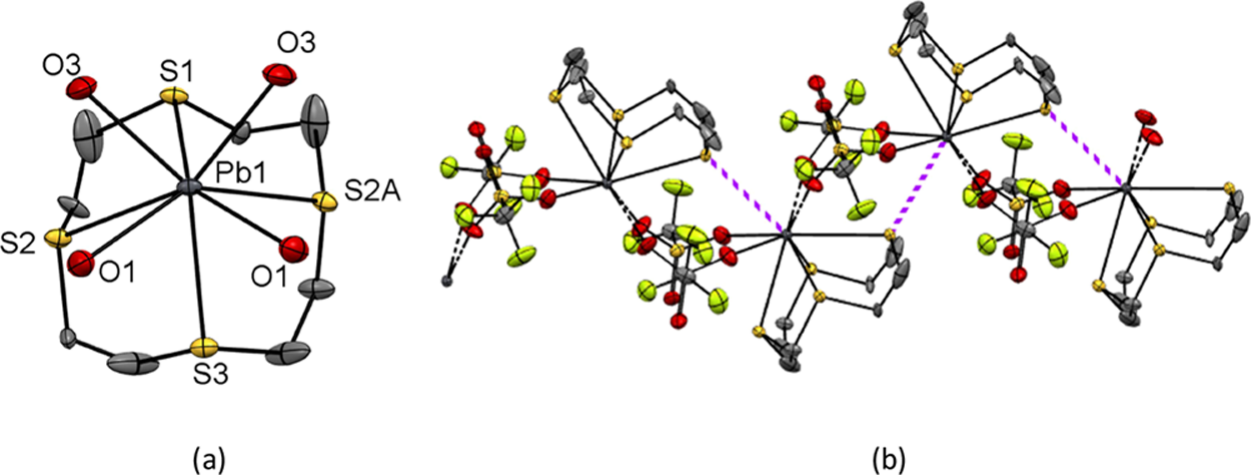
(a) Structure of [Pb([12]aneS_4_)][OTf]_2_ (**8**) showing the atom-labeling scheme. Ellipsoids are drawn at the 50% probability level, and H atoms and the MeCN solvate molecule are omitted for clarity; there is 1:1 disorder in the macrocycle, so only one component is shown. Selected bond lengths (Å) and angles (deg) are as follows: Pb1–S1 = 3.080(3), Pb1–S2 = 3.070(3), Pb1–S2A = 2.995(3) Pb1–S3 = 3.0315(18), Pb1···O1 = 2.688(5), Pb1···O3 = 2.657(4), S1–Pb1–S2 = 67.84(8), S2–Pb1–S3 = 67.85(6), S3–Pb1–S2A = 71.41(6), S2A–Pb1–S1 = 68.87(9) S1–Pb1–S3 = 106.17(6), S2–Pb1–S2A = 106.65(10); (b) extended structure of [Pb([12]aneS_4_)][OTf]_2_ with the long bridging Pb–S···Pb interactions shown as purple dashed lines.

Similarly, [Pb([12]aneS_4_)][OTf]_2_ (**8**) has the macrocycle bound in an endocyclic κ^4^-coordination mode with four similar Pb–S distances. However, here there is an additional weak contact to a thioether S atom from a neighboring unit (Pb···S = 3.377(3) Å), along with four triflates bridging between adjacent lead centers, giving rise to a 1D polymer (Figure 5), with a nine-coordinate geometry at lead (S_5_O_4_ donor set). Moreover, the Pb–O(triflate) bonds are much shorter than the Pb–S bonds, contrasting with the Ge and Sn analogues, where the opposite trend is observed.

Comparing the three [12]aneS_4_ homologues, the change in coordination behavior suggests a weakening of the M–S(thioether) interactions and a strengthening of the M–O(triflate) interactions as the group is descended (Table 2).

**Table 2:** Selected Geometric Parameters for [M([12]aneS_4_)][OTf]_2_ (M = Ge, Sn, Pb)

[M([12]aneS_4_)][OTf]_2_	M = Ge (**2**)	M = Sn (**5**)	M = Pb (**8**)
*d*(M–S)/Å	2.4446(4)	2.8160(8)	3.0315(18)
	2.5050(4)	2.8176(8)	3.070(3)
	2.7338(5)	2.8675(8)	2.995(3)
	2.7717(5)	2.9053(9)	3.080(3)
			3.377(3) (bridging)
*d*(M···O)/Å	2.8002(14)	2.876(3)	2.657(4)
	2.8018(14)	2.887(2)	2.688(5)
		2.902(3)	
		3.024(3)	

#### [24]aneS_8_ Complexes of Ge(II), Sn(II), and Pb(II)

To test the effect on the coordination geometries and donor sets and to probe whether homoleptic S_8_-coordination might be possible (cf. the [Ge(12-crown-4)_2_]^2+^ cation^12,13^), the complexes of the larger octathia-macrocycle, [24]aneS_8_, were also prepared (Scheme 1) and isolated in good yields as white powdered solids.

**Figure 6:**
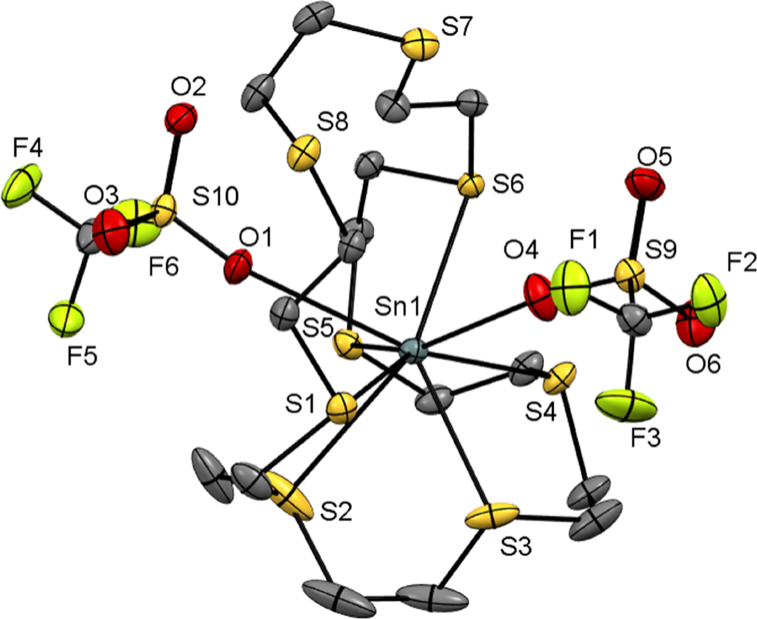
. Structure of [Sn([24]aneS_8_)(OTf)_2_] (**6**) showing the atom-labeling scheme; there are two molecules in the asymmetric unit, but only one is shown. Ellipsoids are drawn at the 50% probability level, and H atoms are omitted for clarity. Selected bond lengths (Å) and angles (deg) are as follows: Sn1–S1 = 3.2327(7), Sn1–S2 = 2.9623(7), Sn1–S3 = 2.9025(7), Sn1–S4 = 2.8530(7), Sn1–S5 = 2.9925(6), Sn1–S6 = 3.0594(6) Sn1–O1 = 2.9873(19), Sn1–O4 = 2.539(2).

In the case of [Sn([24]aneS_8_)][OTf]_2_ (**6**), single crystals were grown by layering a CH_2_Cl_2_/MeCN solution of the complex with hexane. The crystal structure reveals (Figure 6) that the macrocycle is bound in a κ^6^-coordination mode through six sequential (adjacent) S-donor atoms (S1–S6), with the two remaining sulfur atoms (S7 and S8) uncoordinated. The coordination environment is completed by one short and one long contact to nearby triflates (2.539(2) and 2.9873(19) Å), suggesting that one triflate is coordinated, giving a monocationic salt, [Sn(OTf)([24]aneS_8_)][OTf]; this description is supported by the distribution of S–O bond lengths within the OTf groups. The Sn–S bond lengths are longer compared to those in the smaller ring thia-macrocycles discussed above, in this case ranging from 2.8530(7) to 3.2327(7) Å, suggesting a rather poor size match between the macrocycle and the Sn(II) center.

**Figure 7:**
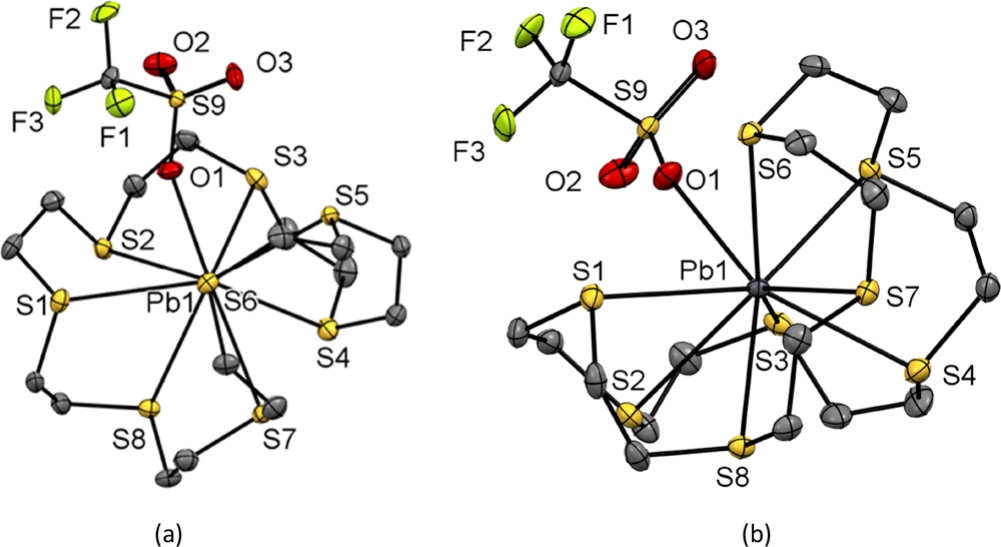
Two views of the structure of the cation in Pb([24]aneS_8_)(OTf)][OTf] (**9**) showing the atom-labeling scheme (there are two molecules in the asymmetric unit, but only one is shown). Ellipsoids are drawn at the 50% probability level, and H atoms, the MeCN solvate, and the noncoordinated triflate are omitted for clarity. Selected bond lengths (Å) and angles (deg) are as follows: Pb1–S1 = 3.1320(9), Pb1–S2 = 3.2627(9), Pb1–S3 = 3.0889(10), Pb1–S4 = 3.0674(10), Pb1–S5 = 2.9665(9), Pb1–S6 = 3.0620(9), Pb1–S7 = 3.1320(9), Pb1–S8 = 3.1770(9), Pb1–O1 = 2.645(3).

In contrast, for [Pb([24]aneS_8_)(OTf)][OTf] (**9**), the crystal structure (Figure 7a,b) reveals that the macrocycle is coordinated via all eight S-donor atoms, i.e., in an endocyclic κ^8^-mode, with one triflate also coordinated, to give a nine-coordinate lead(II) monocation. Five of the S atoms lie in a pentagonal plane with the lead atom sitting 0.76 Å below this plane. The remaining three sulfur atoms coordinate in a pyramidal fashion on the opposite face of the lead(II) center. The Pb–S distances span 2.9665(9)–3.2627(9) Å and are much longer than the distances seen for the complex [Pb([9]aneS_3_)][OTf]_2_ (**3**) and more similar to those observed in [Pb([12]aneS_4_)][OTf]_2_ (**6**). Considering the additional coordinated OTf ligand, the geometry at Pb(II) is best described as a monocapped square antiprism, with atoms S2, S3, S4, and S8 forming the bottom face, S1, S5, S7, and O1 forming the top face, and atom S6 in the capping position, as illustrated in Figure 7a,b. This can be compared to previously reported dinuclear [Pb_2_([24]aneS_8_)][ClO_4_]_4_ complex,^20b^ containing two lead(II) centers. Although the crystal structure of this species is not known, the analogous complex with the larger [28]aneS_8_ has been structurally characterized, confirming two lead centers bound within the macrocycle, bridged by the ClO_4_^–^ anions.

It is extremely rare for [24]aneS_8_ to adopt κ^8^-coordination to a single metal center, the only other structurally characterized example being [Na([24]aneS_8_)][BAr^F^].^8b^

#### NMR and MS Characterization

The bulk products were also characterized by a combination of microanalysis, IR spectroscopy, as well as solution ^1^H, ^13^C{^1^H}, ^19^F{^1^H}, and ^119^Sn{^1^H} NMR spectroscopy, where appropriate. The ESI^+^ MS of all of the nine complexes each show clusters of peaks with the expected isotopic pattern corresponding to [M(macrocycle)(OTf)]^+^, and for all except [Ge([24]aneS_8_)]^2+^, they also show clusters of peaks associated with the free dications, [M(macrocycle)]^2+^. The solution NMR spectra of complexes containing [9]aneS_3_ and [12]aneS_4_ were recorded in CD_3_CN as their solubility in (the more weakly coordinating) CD_2_Cl_2_ was poor; however, the [24]aneS_8_ complexes were sufficiently soluble to allow CD_2_Cl_2_ to be used as the NMR solvent. The ^1^H and ^13^C{^1^H} NMR spectra for each of the complexes show a singlet resonance from the macrocycle, and in all cases, the chemical shift is significantly to high frequency from the macrocycle itself, providing evidence for retention of the [M(macrocycle)]^2+^ coordination in solution. This contrasts with the behavior observed for [GeCl_2_([9]aneS_3_)], where the NMR spectra in CD_2_Cl_2_ are unchanged from [9]aneS_3_ itself, indicating that it is extensively dissociated in solution.

For all of the complexes reported here, the ^19^F{^1^H} NMR spectra show a single sharp resonance very close to −79.3 ppm (in MeCN) or −78.6 ppm (in CH_2_Cl_2_), which correspond to discrete ionic triflate in solution, indicating that the interactions between the triflate and the metal centers seen in the solid-state crystal structures are lost in solution. For the tin complexes, ^119^Sn{^1^H} NMR spectra show singlet resonances at −737 ppm for [Sn([9]aneS_3_][OTf]_2_ (**2**), −903 ppm for [Sn([12]aneS_4_)][OTf]_2_ (**5**), and −1079 ppm for [Sn([24]aneS_8_)][OTf]_2_ (**8**). We were unable to observe a ^207^Pb NMR resonance for the Pb(II) complexes, possibly due to ligand exchange processes in solution.

#### DFT Calculations

The electronic structures of the complexes reported in this work were also investigated using DFT calculations as described above.

**Figure 8:**
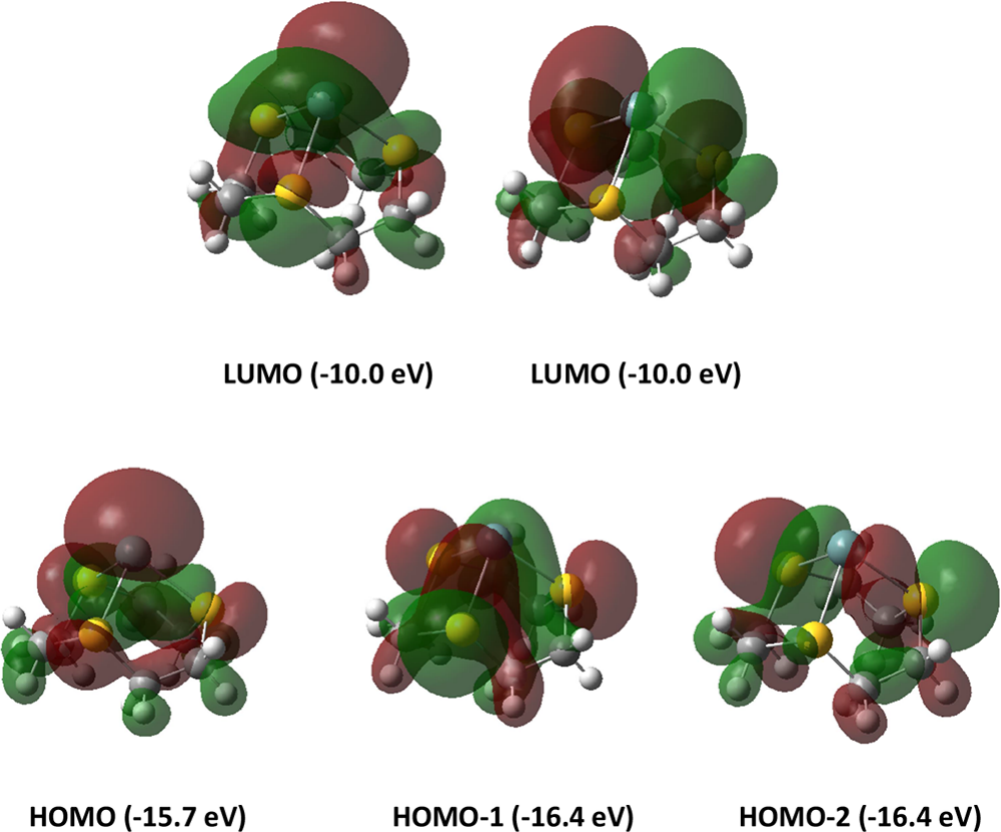
Representations of the frontier orbitals of [Ge([9]aneS_3_)]^2+^.

For [M([9]aneS_3_]^2+^ (M = Ge, Sn, Pb), the HOMO orbital is mainly a M s–p “lone pair” on the group 14 atom (Figure 8). In all cases, the HOMO is directional, having a partial percentage M p_*z*_ character of 6.64, 4.22, and 2.58 for Ge, Sn, and Pb, respectively, consistent with the expected trend down the group. HOMO–1 and HOMO–2 correspond to degenerate bonding orbitals formed from the interaction of the sulfur lone pairs and the p_*x*_ and p_*y*_ orbitals on the metal center. The LUMO and LUMO+1 orbitals are also degenerate and correspond to empty p_*x*_ and p_*y*_ orbitals on the group 14 center. The positive charge on the group 14 center also increases from Ge to Sn to Pb (+0.65*e*, +1.06*e*, +1.13*e*, respectively), consistent with reduced covalent interactions with the thioether ligand.

For [Ge([12]aneS_4_)]^2+^, both the HOMO and HOMO–1 are related to the “lone pair” on germanium, whereas HOMO–2 and HOMO–3 are related to the bonding orbitals between the sulfur atoms and the germanium center. HOMO–2 (−15.28 eV) is higher in energy and is related to the axial sulfurs, while HOMO–3 (−15.63 eV) is related to the equatorial sulfurs. The HOMO for [Sn([12]aneS_4_)]^2+^ is centered on the tin, with HOMO–1 being based on the sulfur atoms and HOMO–2 and HOMO–3 being degenerate orbitals based on the sulfur “lone pairs” interacting with the empty p_*x*_ and p_*y*_ orbitals on tin. In both cases, the lone pair on the metal center is directional. Compared to the analogous complexes with [9]aneS_3_, the charges on the metal center are slightly smaller. For [Ge([12]aneS_4_)]^2+^, the charge is not spread equally among the sulfurs in the ligand, with S3 taking on most of the positive charge (+0.36) and S1 taking on the least (+0.29), while S2/S4 each carry a charge of +0.34. However, in the more symmetrical [Sn([12]aneS_4_)]^2+^, each sulfur takes on an equal amount of positive charge (+0.27).

**Figure 9:**
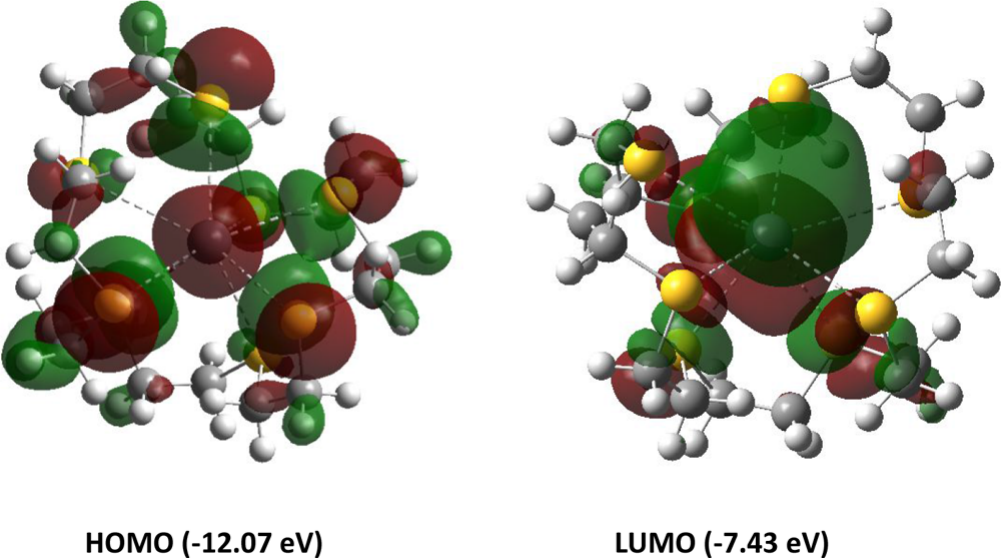
Representations of the frontier orbitals calculated for [Pb([24]aneS_8_)]^2+^.

To better understand the electronic structure of [Pb([24]aneS_8_)]^2+^, DFT calculations were also performed on this dication. The HOMO (Figure 9) is based on lone pairs on the sulfur atoms as well as an s-type lone pair (99.5% s-character) on the lead center. The LUMO is a p_*z*_-based orbital on lead which is orthogonal to the plane of the pentagon. This is consistent with a triflate group interacting with the Pb(II) center as observed in the experimentally determined crystal structure for [Pb([24]aneS_8_)(OTf)][OTf].

## Conclusions

A systematic study of the coordination chemistry of M(II) triflates (M = Ge, Sn, Pb) with three thia-macrocycles, [9]aneS_3_, [12]aneS_4_, and [24]aneS_8_, has been undertaken, and crystal structures were determined for eight of the complexes, all of which are monometallic species with a 1:1 M:macrocycle ratio, irrespective of the macrocycle denticity. The X-ray data also show that changing the anion from a halide to the more weakly coordinating triflate induces endocyclic coordination in all cases, with significant strengthening of the M–S interactions evidenced by the contraction of the M–S bond lengths, and increases the number of thioether donor atoms coordinated. This leads to the complexes displaying a wide range of coordination numbers, from three to nine.

The strength of the cation–anion interactions varies with both macrocycle and the group 14 center. In some cases, the triflate anion is clearly coordinated to the metal center, whereas in others, only very weak M···O_3_SCF_3_ interactions are present. DFT calculations reveal that the nature of the HOMO on the metal center (M s–p) also varies across the series, with [Pb([24]aneS_8_)]^2+^ having an s-type lone pair, whereas [Pb([9]aneS_3_)]^2+^ has a mixture of s- and p-character. Further, for the homologous series, [M([9]aneS_3_)]^2+^ (M = Ge, Sn, Pb), the p-character increases up the group. These results point to the possibility that some of the divalent group 14 thia-macrocyclic complexes may be capable of both donor and acceptor behavior, which will be the focus of our future work in this area.

## Supporting Information Available

Crystallographic parameters for the crystal structures reported; full details of the computational work; and original IR, ^1^H, ^13^C{^1^H}, ^19^F{^1^H}, and ^119^Sn{^1^H} NMR spectra for the reported complexes (PDF)
X-ray crystallographic parameters of complexes (XYZ)

## Supplementary Material

ic2c03613_si_001.pdf

ic2c03613_si_002.xyz
