# Supplementary material for: Structural Diversity in Divalent Group 14 Triflate Complexes Involving Endocyclic Thia-Macrocyclic Coordination
Source: Inorg Chem. 2023 Jan 5;62(2):853–62. doi: 10.1021/acs.inorgchem.2c03613 (PMC9846692; doi:10.1021/acs.inorgchem.2c03613)
Supplement: Supplementary file 1 — ic2c03613_si_001.pdf [file ic2c03613_si_001.pdf]

Supporting Information for

Structural diversity in divalent group 14 triflate complexes involving *endocyclic* thia-macrocyclic coordination

Rhys P. King, Julie M. Herniman, William Levason and Gillian Reid*

School of Chemistry, University of Southampton, Southampton SO17 1BJ, UK. Email: G.Reid@soton.ac.uk

Table S1 X-ray crystallographic parameters[a]

Complex	$[Ge([9]aneS_3)][OTf]_2$ **(1)**	$[Sn([9]aneS_3)][OTf]_2$ **(4)**	$[Pb([9]aneS_3)][OTf]_2$ **(7)**	$[Ge([9]aneS_3)][OTf]_2\cdot MeCN$ (**1**·MeCN)
Formula	$C_8H_{12}F_6GeO_6S_5$	$C_8H_{12}F_6O_6S_5Sn$	$C_8H_{12}F_6O_6PbS_5$	$C_{10}H_{12}F_6GeNO_6S_5$
M	551.07	597.17	685.67	589.10
Crystal system	Monoclinic	Monoclinic	Monoclinic	Triclinic
Space group (no.)	$P2_1/c$ (14)	$P2_1/c$ (14)	$P2_1/c$ (14)	$P\bar{1}$ (2)
a /Å	11.7461(4)	11.6221(3)	11.6163(4)	10.3807(3)
b /Å	10.7825(3)	10.9594(3)	10.8809(3)	10.3989(2)
c /Å	15.0742(5)	15.2267(3)	15.4150(5)	11.3624(3)
α /°	90	90	90	71.191(2)
β /°	109.258(4)	109.412(2)	109.207(4)	64.344(3)
γ /°	90	90	90	89.259(2)
U /Å^3	1902.35(11)	1829.19(8)	1839.94(11)	1034.98(5)
Z	4	4	4	2
μ(Mo-K$_\alpha$) /mm^{-1}	2.362	2.050	2.475	1.890
F(000)	1096	1168	1296	586
Total number reflns	14417	19164	24801	28943
R_{int}	0.035	0.030	0.067	0.042
Unique reflns	5208	5923	5399	6244
No. of params, restraints	235, 0	235, 0	229, 0	265, 0
GOF	1.047	1.088	1.062	1.075
R_1, wR_2 [$I > 2\sigma(I)$][b]	0.039, 0.084	0.034, 0.070	0.034, 0.072	0.033, 0.079
R_1, wR_2 (all data)	0.052, 0.089	0.040, 0.073	0.043, 0.075	0.042, 0.082

[a] common items: T = 100 K; wavelength (Mo-K$_\alpha$) = 0.71073 Å; θ(max) = 27.5°;

[b] $R_1 = \Sigma||F_o| - |F_c||/\Sigma|F_o|$; $wR_2 = [\Sigma w(F_o^2 - F_c^2)^2 / \Sigma wF_o^4]^{1/2}$

Table S1 (continued)

Complex	$[Ge([12]aneS_4)][OTf]_2$ **(4)**	$[Sn([12]aneS_4)][OTf]_2\cdot 2MeCN$ (**5**·2MeCN)	$[Pb([12]aneS_4)][OTf]_2\cdot MeCN$ (**8**·MeCN)
Formula	$C_{10}H_{16}F_6GeO_6S_6$	$C_{14}H_{22}F_6N_2O_6S_6Sn$	$C_{12}F_6NO_2PbS_6$
M	611.18	739.38	767.68
Crystal system	Triclinic	Monoclinic	Orthorhombic
Space group (no.)	$P\bar{1}$ (2)	$P2_1/c$ (14)	Pnma (62)
a /Å	8.1431(2)	12.3149(2)	10.2174(4)
b /Å	11.2847(3)	15.0799(2)	16.3506(10)
c /Å	12.4536(4)	15.4727(3)	14.3326(6)
α /°	68.646(3)	90	90
β /°	81.022(2)	110.852(2)	90
γ /°	74.790(2)	90	90
U /Å^3	1026.16(5)	2685.20	2394.4(2)
Z	2	4	4
μ(Mo-K_α) /mm^{-1}	1.978	1.493	7.643
F(000)	612	1472	1436
Total number reflns	19105	27380	22797
R_{int}	0.030	0.035	0.079
Unique reflns	4606	8048	3789
No. of params, restraints	262, 0	318, 0	184, 0
GOF	1.065	1.028	1.023
R_1, wR_2 [$I > 2\sigma(I)$]^b	0.025, 0.067	0.048, 0.116	0.046, 0.098
R_1, wR_2 (all data)	0.026, 0.067	0.056, 0.123	0.071, 0.107

[a] common items: T = 100 K; wavelength (Mo-K_α) = 0.71073 Å; θ(max) = 27.5°;

[b] $R_1 = \Sigma||F_o| - |F_c||/\Sigma|F_o|$; $wR_2 = [\Sigma w(F_o^2 - F_c^2)^2/\Sigma wF_o^4]^{1/2}$

Table S1 (continued)

Complex	$[Sn([24]aneS_8)(OTf)][OTf]$ **(6)**	$[Pb([24]aneS_8)(OTf)][OTf]\cdot MeCN$ (**9**·MeCN)
Formula	$C_{36}H_{53}F_{12}O_{12}S_{20}Sn_2$	$C_{20}H_{35}F_6NO_6PbS_{10}$
M	1784.36	1027.28
Crystal system	Triclinic	Triclinic
Space group (no.)	$P\bar{1}$ (2)	$P\bar{1}$ (2)
a /Å	12.0184(2)	14.5533(2)
b /Å	14.5648(2)	14.7594(2)
c /Å	19.4918(3)	19.4079(3)
α /°	77.2080(10)	69.0880(10)
β /°	76.7400(10)	83.9860(10)
γ /°	89.2220(10)	64.109(2)
U /Å^3	3236.07(9)	3496.83(10)
Z	2	4
μ(Mo-K$_\alpha$) /mm^{-1}	1.503	5.490
F(000)	1786	2024
Total number reflns	87644	94969
R_{int}	0.035	0.053
Unique reflns	19109	21366
No. of params, restraints	801, 0	845, 0
GOF	1.066	1.054
R_1, wR_2 [$I > 2\sigma(I)$][b]	0.042, 0.096	0.038, 0.081
R_1, wR_2 (all data)	0.047, 0.100	0.048, 0.086

[a] common items: T = 100 K; wavelength (Mo-K$_\alpha$) = 0.71073 Å; θ(max) = 27.5°;

[b] $R_1 = \Sigma||F_o| - |F_c||/\Sigma|F_o|$; $wR_2 = [\Sigma w(F_o^2 - F_c^2)^2 / \Sigma wF_o^4]^{1/2}$

Figure S1 [Ge([9]aneS$_3$)][OTf]$_2$ **(1)**

S1.1 ^{1}H NMR spectrum of [Ge([9]aneS$_3$)][OTf]$_2$ (CD$_3$CN*, 298 K):

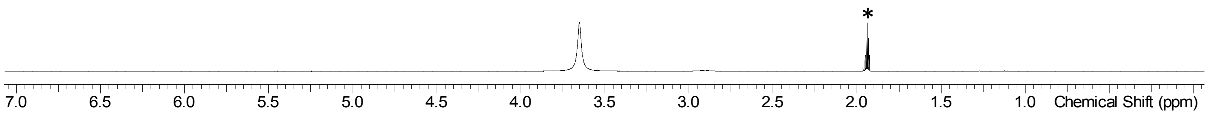

S1.2 ^{13}C{^{1}H} NMR spectrum of [Ge([9]aneS$_3$)][OTf]$_2$ (CD$_3$CN*, 298 K):

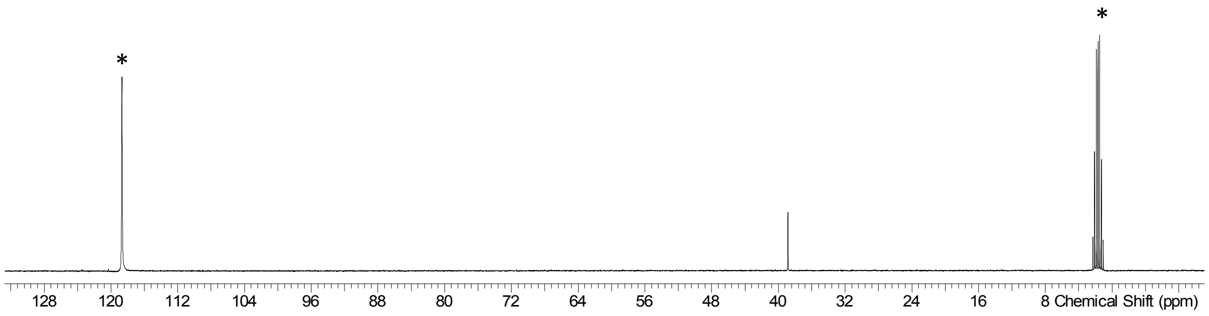

S1.3 ^{19}F{^{1}H} NMR spectrum of [Ge([9]aneS$_3$)][OTf]$_2$ (CD$_3$CN, 298 K):

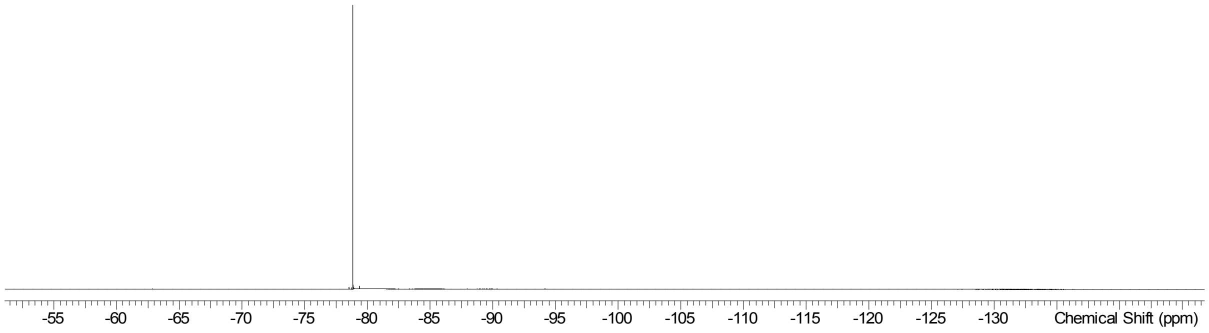

S1.4 IR spectrum of [Ge([9]aneS_3)][OTf]$_2$ (Nujol)

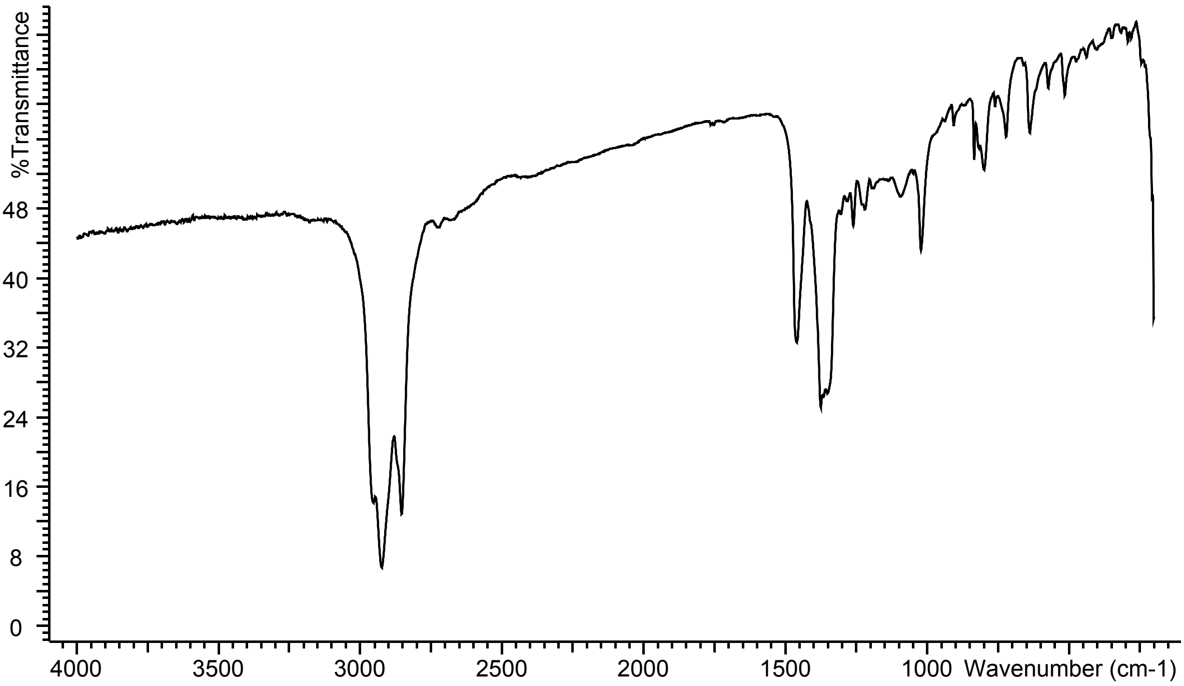

S1.5 HRMS (ESI$^+$, MeCN) of [Ge([9]aneS_3)][OTf]$_2$ top: experimental; bottom: simulated for [Ge([9]aneS_3)(OTf)]$^+$

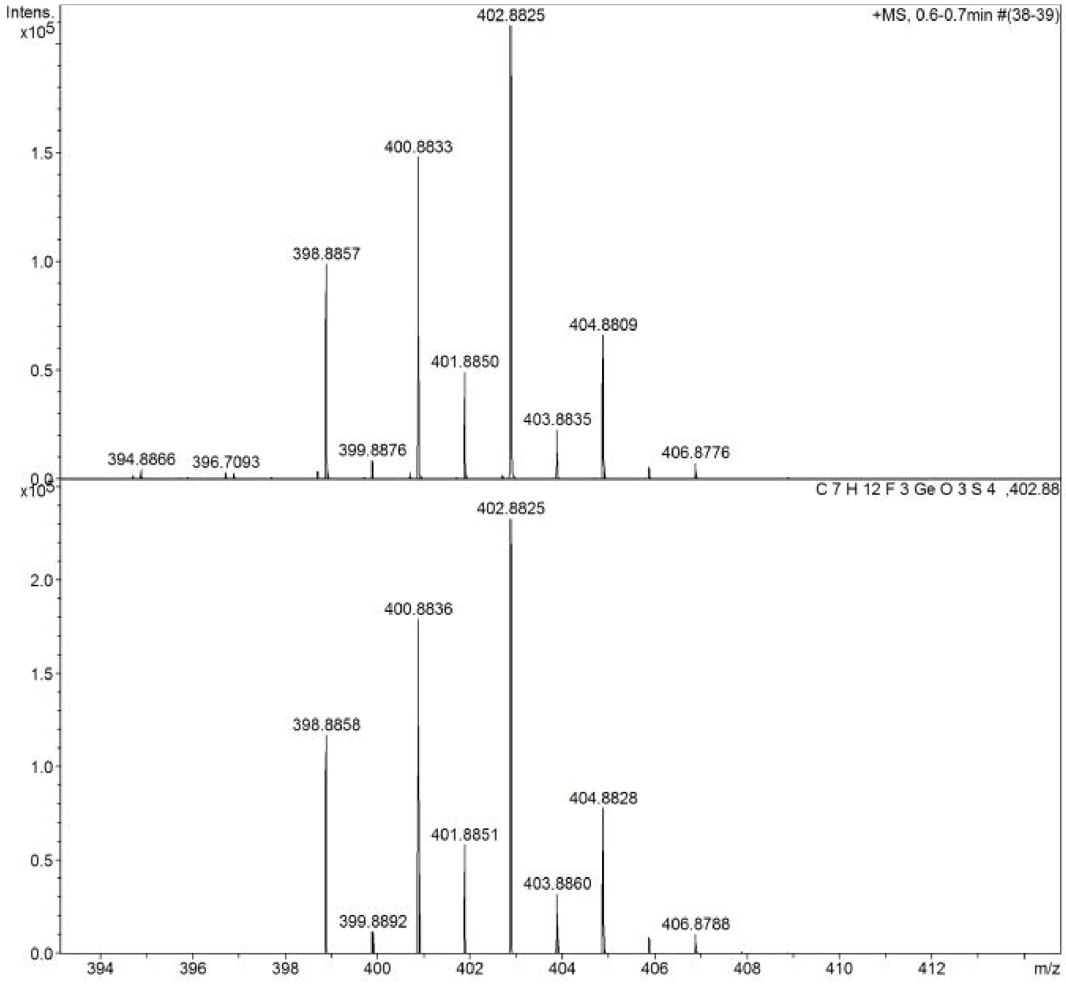

S1.6 HRMS (ESI^+, MeCN) of $[Ge([9]aneS_3)][OTf]_2$ top: experimental; bottom: simulated for $[Ge([9]aneS_3)]^{2+}$

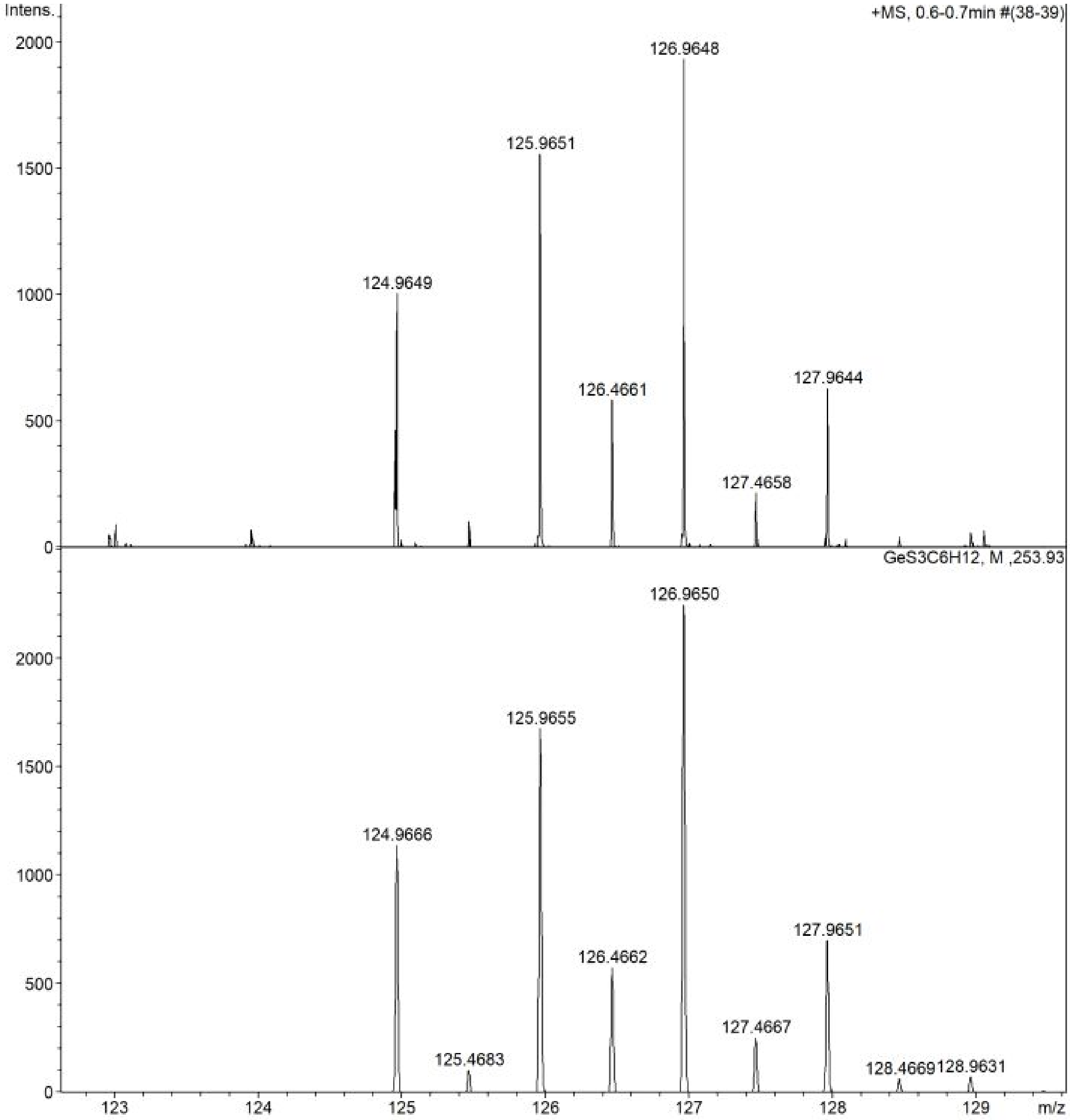

Figure S2 [Sn([9]aneS$_3$)][OTf]$_2$ **(2)**

S2.1 ^{1}H NMR spectrum of [Sn([9]aneS$_3$)][OTf]$_2$ (CD$_3$CN*, 298 K):

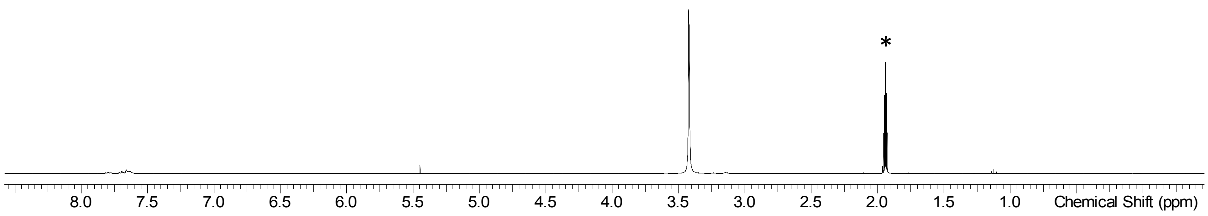

S2.2 ^{13}C{^{1}H} NMR spectrum of [Sn([9]aneS$_3$)][OTf]$_2$ (CD$_3$CN*, 298 K):

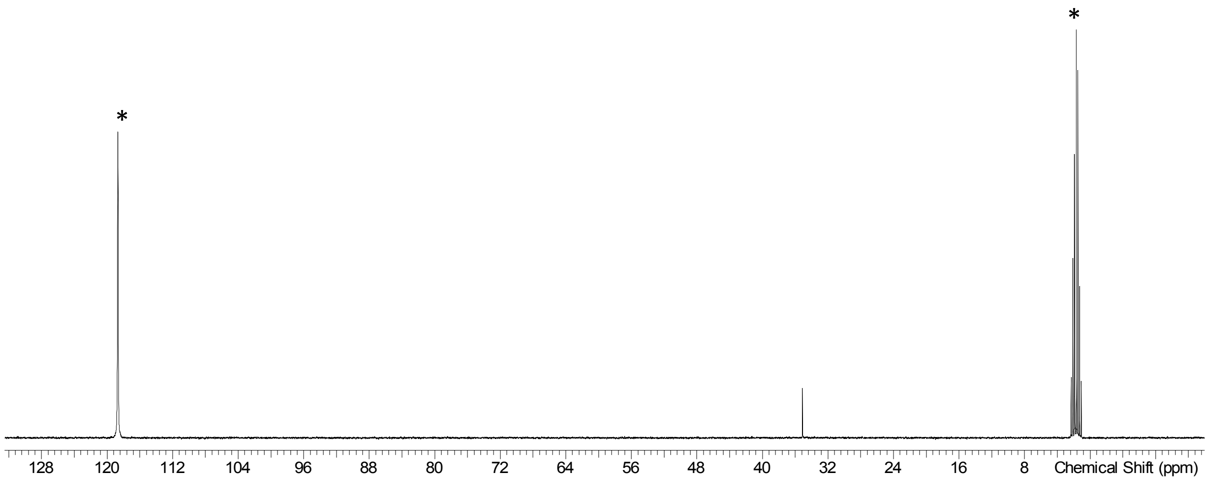

S2.3 ^{19}F{^{1}H} NMR spectrum of [Sn([9]aneS$_3$)][OTf]$_2$ (CD$_3$CN, 298 K):

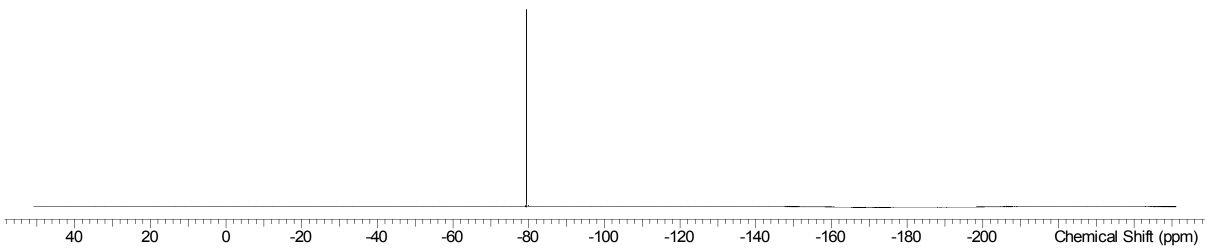

S2.4 $^{119}Sn\{^{1}H\}$ NMR spectrum of $[Sn([9]aneS_3)][OTf]_2$ (CD_3CN, 298 K):

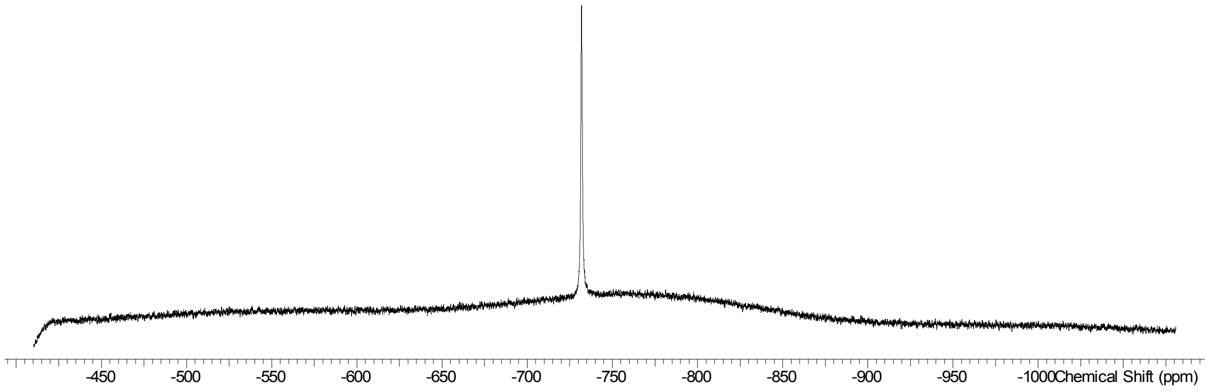

S2.5 IR spectrum of $[Sn([9]aneS_3)][OTf]_2$ (Nujol)

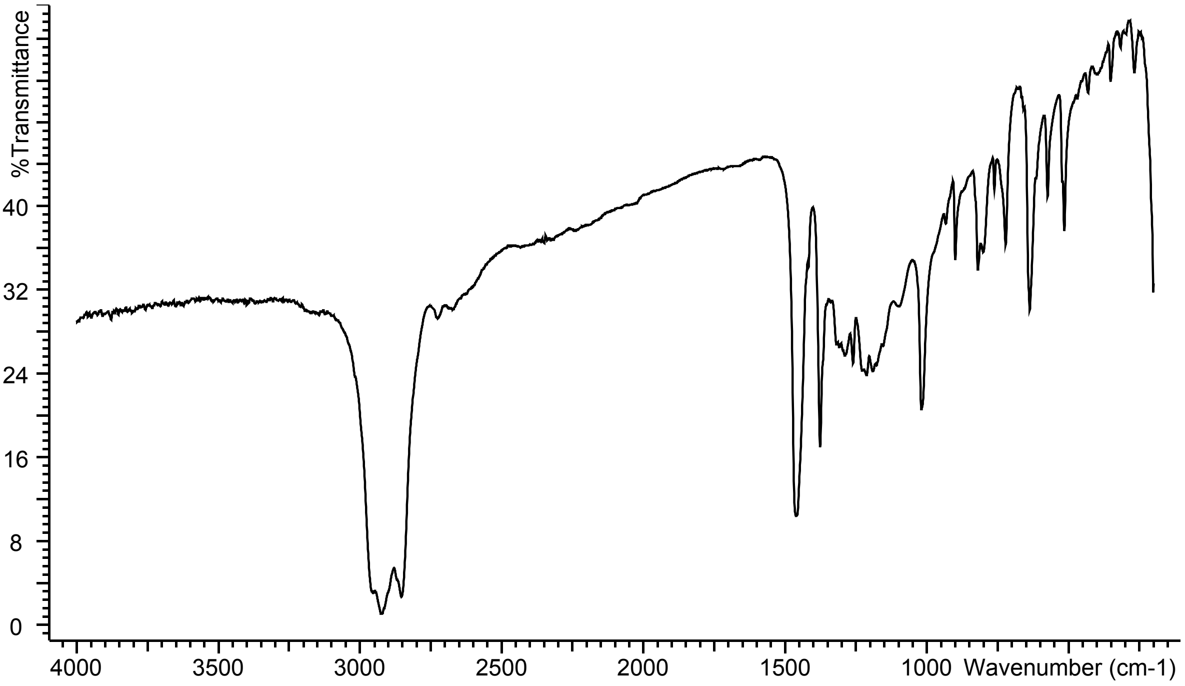

S2.6 HRMS (ESI$^+$, MeCN) top: experimental; bottom: simulated for [Sn([9]aneS$_3$)(OTf)]$^+$

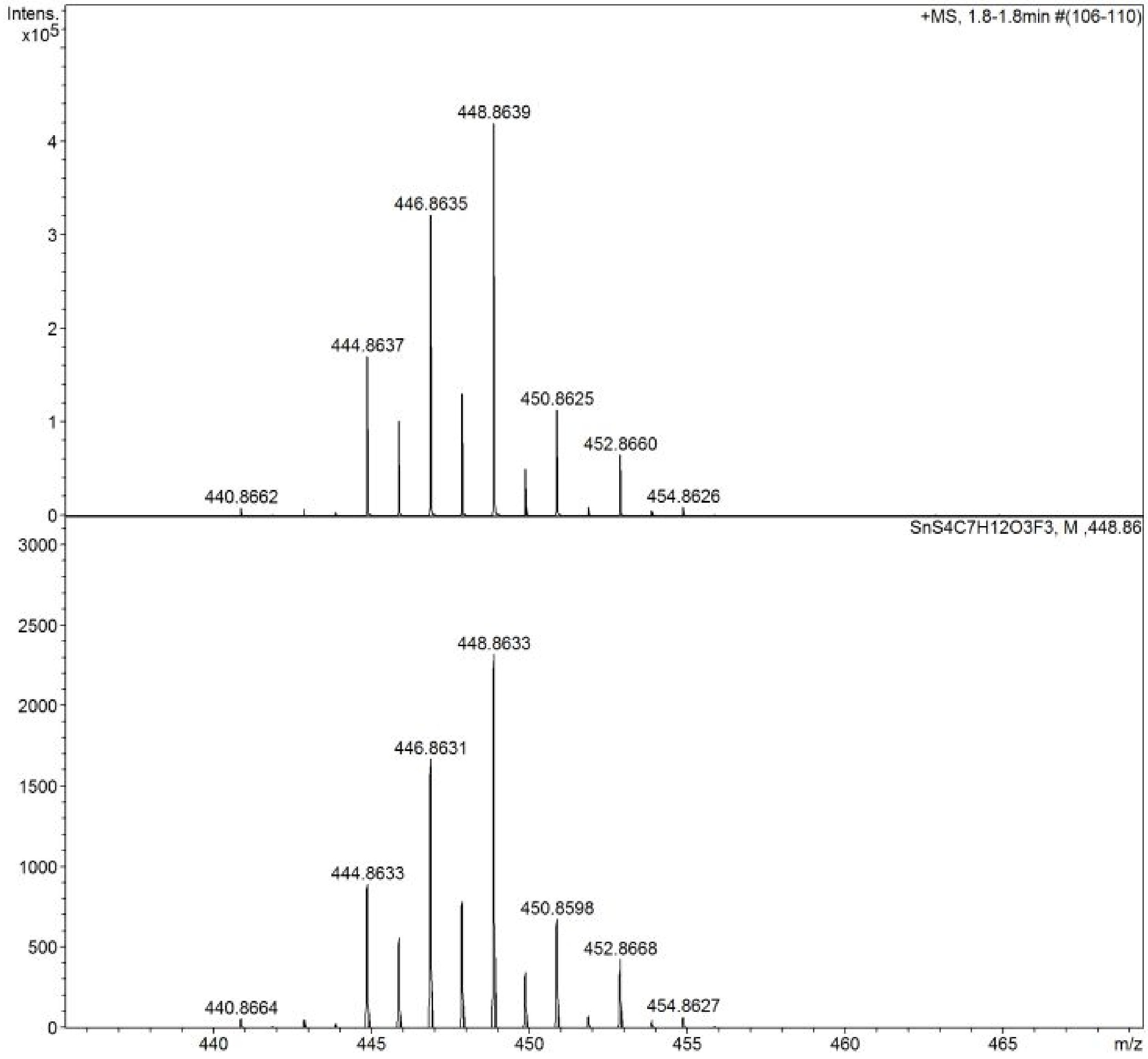

S2.7 HRMS (ESI+, MeCN) (top: experimental; bottom: simulated for $[Sn([9]aneS_3)]^{2+}$

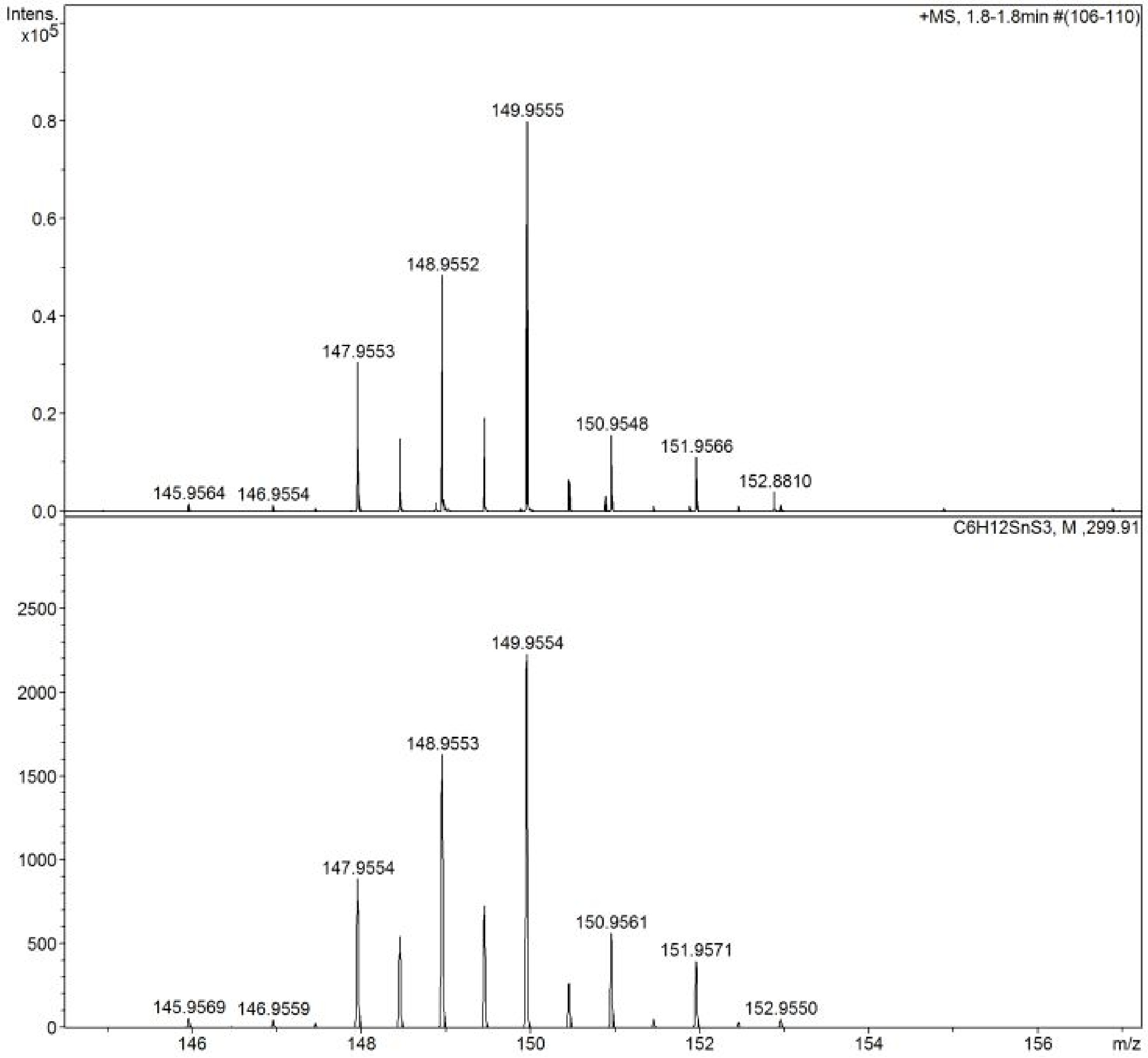

Figure S3 $[Pb([9]aneS_3)][OTf]_2$ **(3)**

S3.1 ^1H NMR spectrum of $[Pb([9]aneS_3)][OTf]_2$ (CD_3CN*, 298 K):

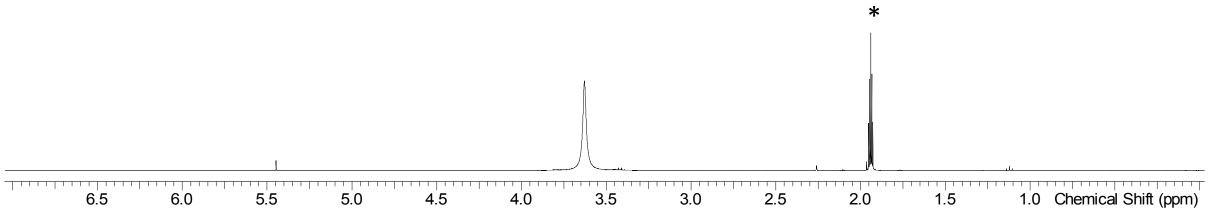

S3.2 $^{13}C\{^{1}H\}$ NMR spectrum of $[Pb([9]aneS_3)][OTf]_2$ (CD_3CN*, 298 K):

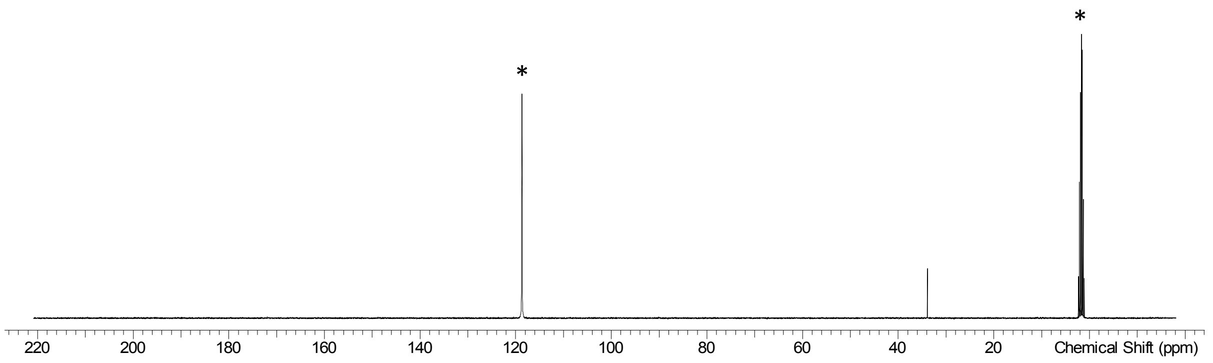

S3.3 $^{19}F\{^{1}H\}$ NMR spectrum of $[Pb([9]aneS_3)][OTf]_2$ (CD_3CN, 298 K):

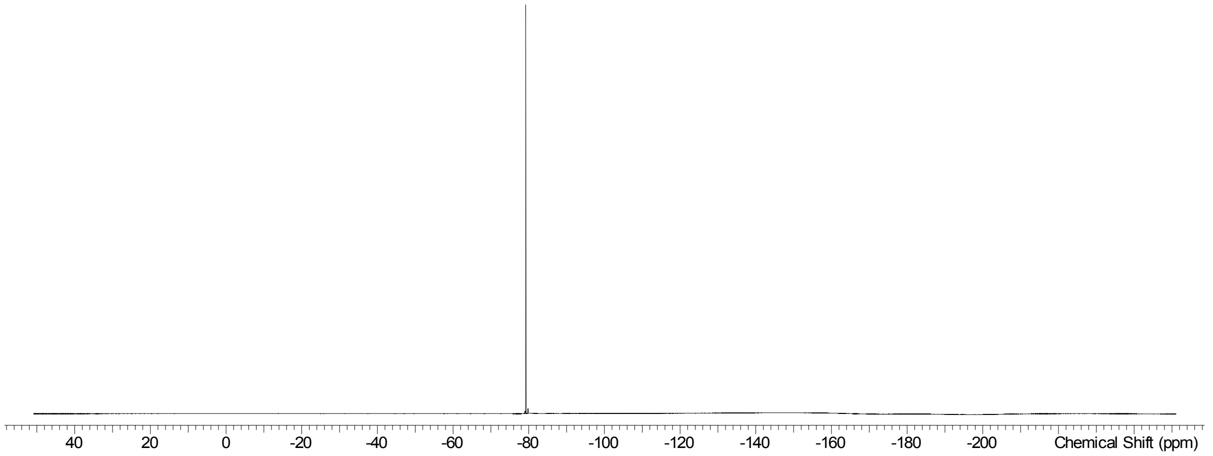

S3.4 IR spectrum of $[Pb([9]aneS_3)][OTf]_2$ (Nujol)

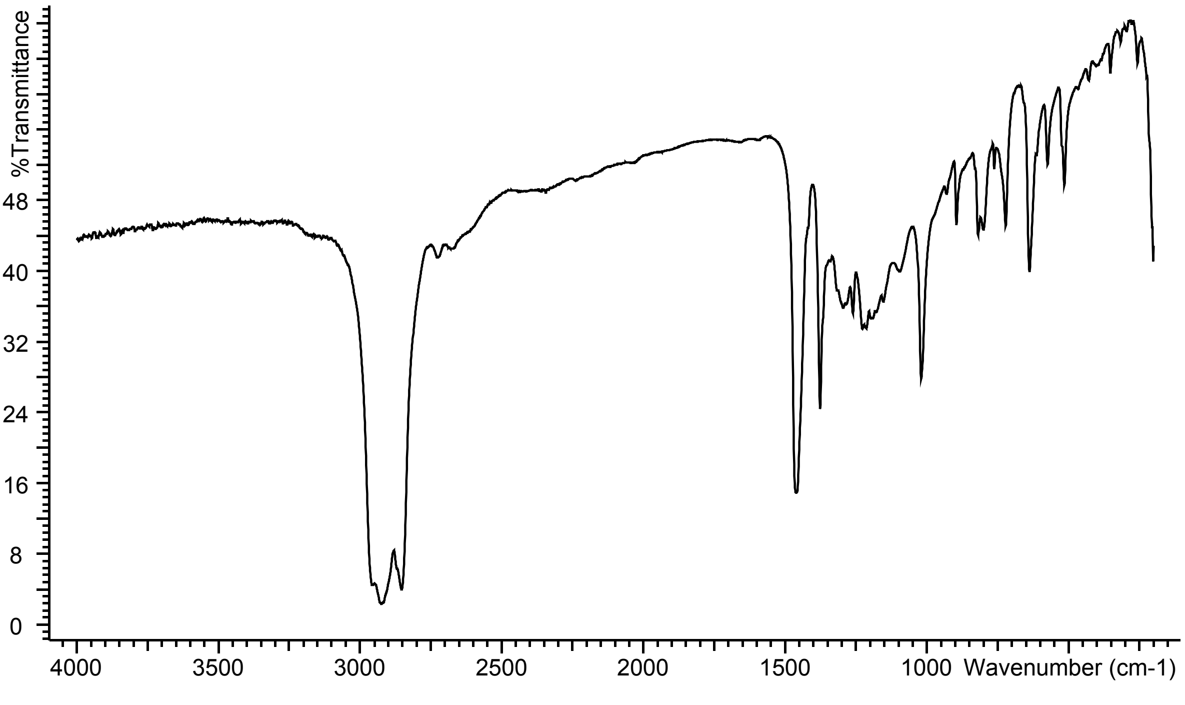

S3.5 HRMS (ESI$^+$, MeCN) (top: experimental; bottom: simulated for [Pb([9]aneS$_3$)(OTf)]$^+$

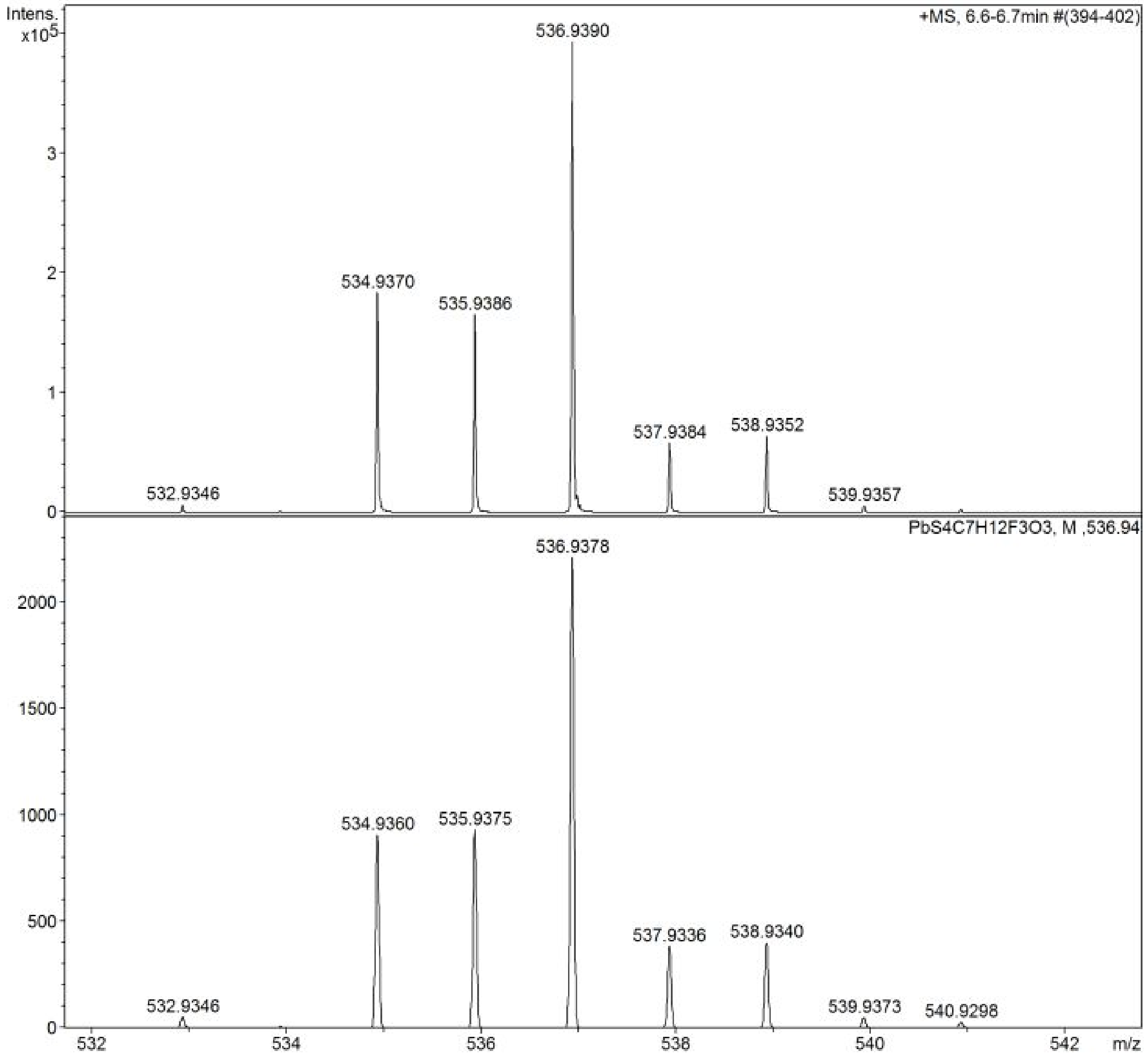

S3.6 HRMS (ESI$^+$, MeCN) (top: experimental; bottom: simulated for $[Pb([9]aneS_3)]^{2+}$

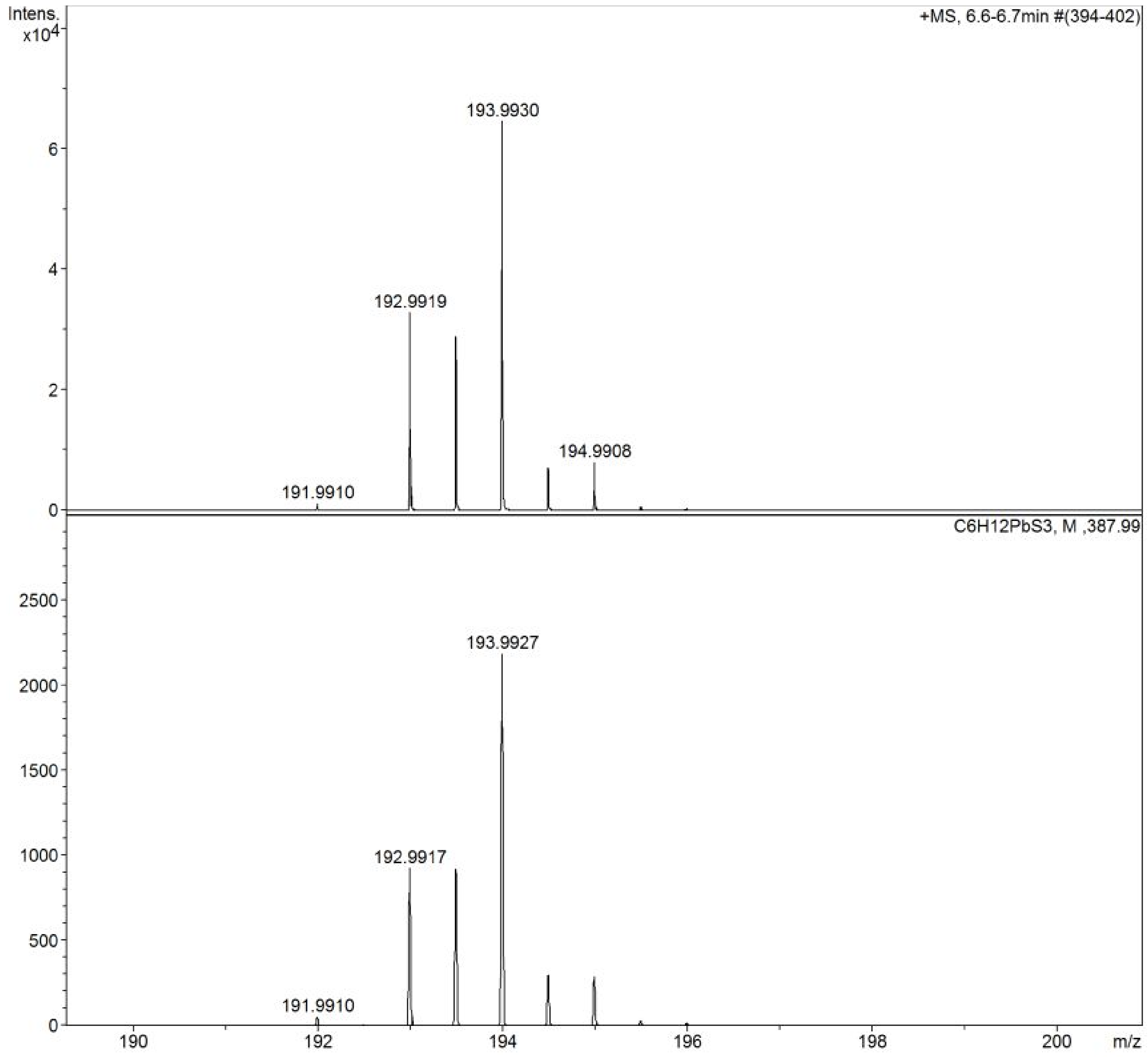

Figure S4 [Ge([12]aneS$_4$)][OTf]$_2$ **(4)**

S4.1 ^{1}H NMR spectrum of [Ge([12]aneS$_4$)][OTf]$_2$ (CD_3CN*, 298 K):

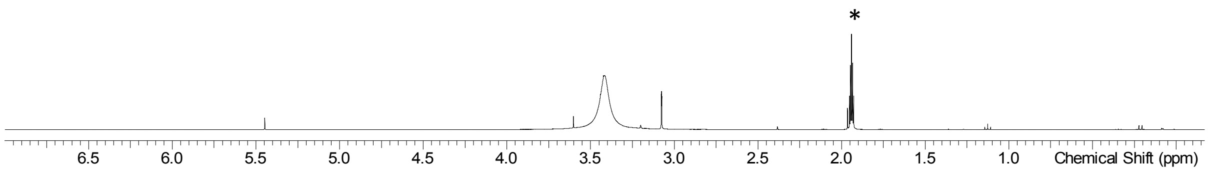

S4.2 ^{13}C{^{1}H} NMR spectrum of [Ge([12]aneS$_4$)][OTf]$_2$ (CD_3CN*, 298 K):

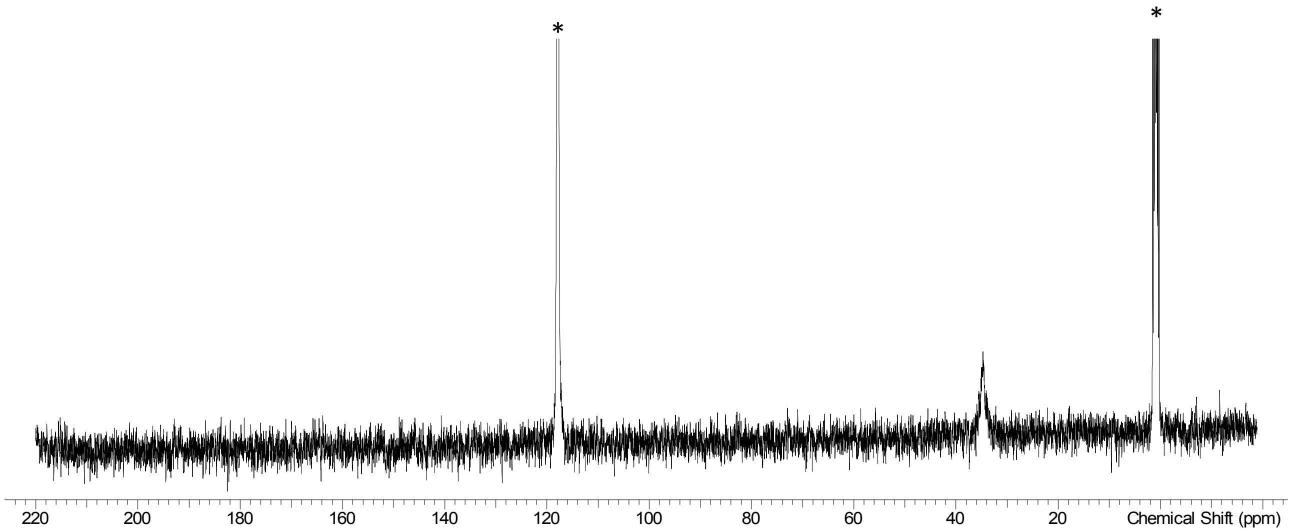

S4.3 ^{19}F{^{1}H} NMR spectrum of [Ge([12]aneS$_4$)][OTf]$_2$ (CD_3CN, 298 K):

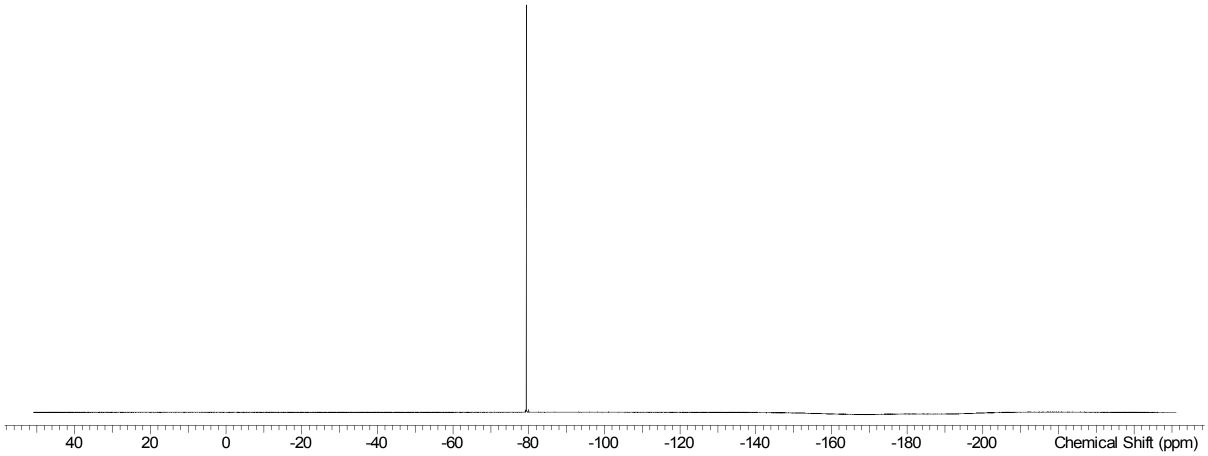

S4.4 IR spectrum of [Ge([12]aneS_4)][OTf]$_2$ (Nujol)

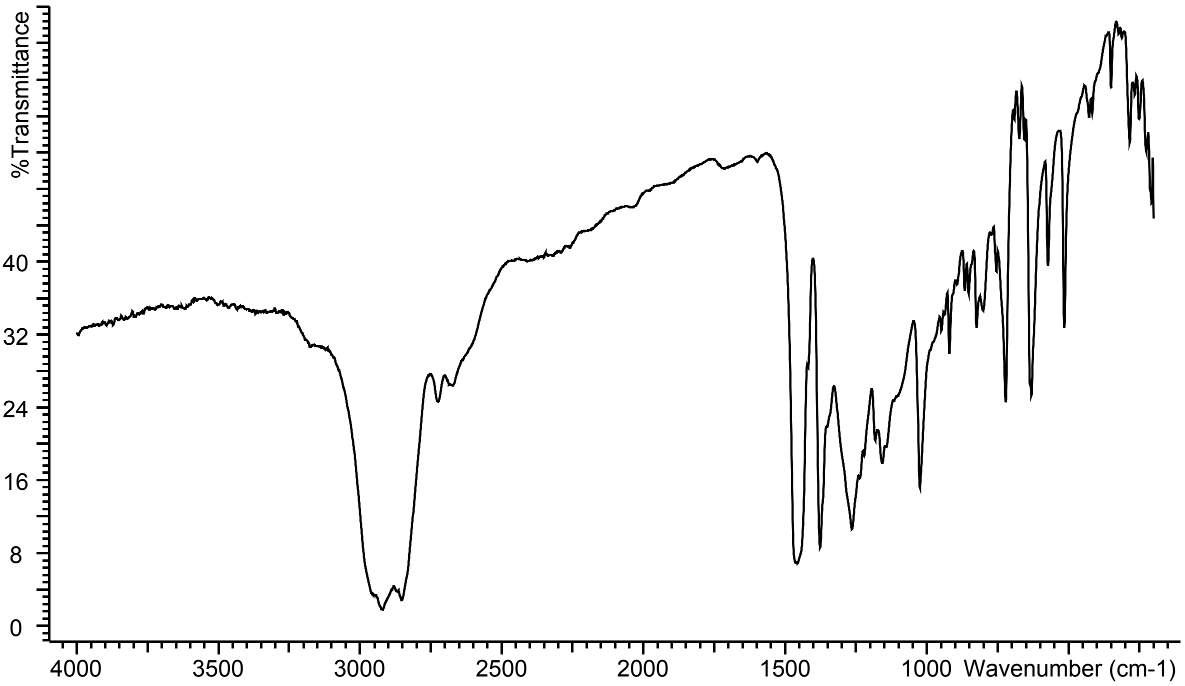

S4.5 HRMS (ESI$^+$, MeCN) top: experimental; bottom: simulated for $[Ge([12]aneS_4)(OTf)]^+$

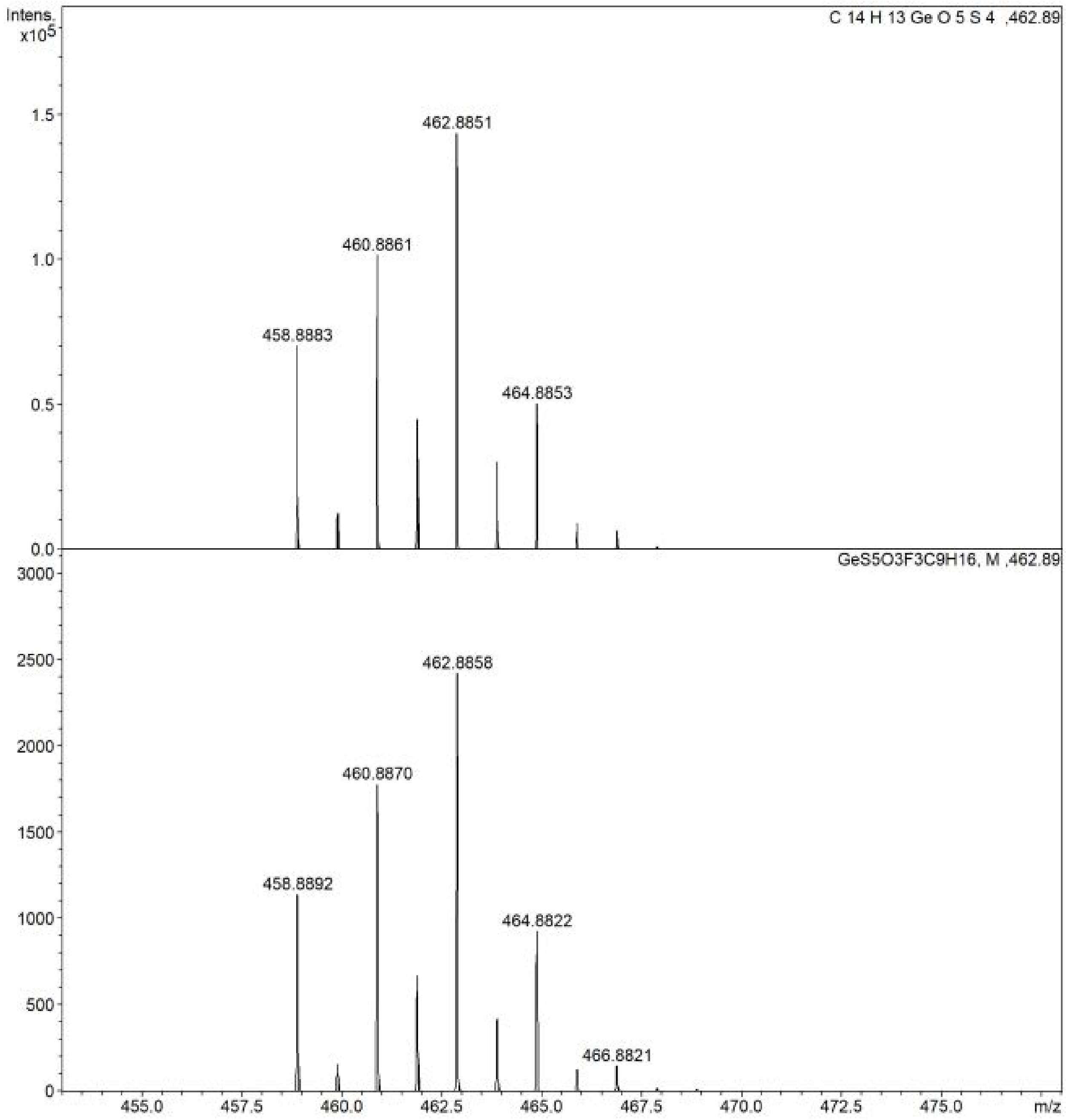

S4.6 HRMS (ESI$^+$, MeCN) top: experimental; bottom: simulated for $[Ge([12]aneS_4)]^{2+}$

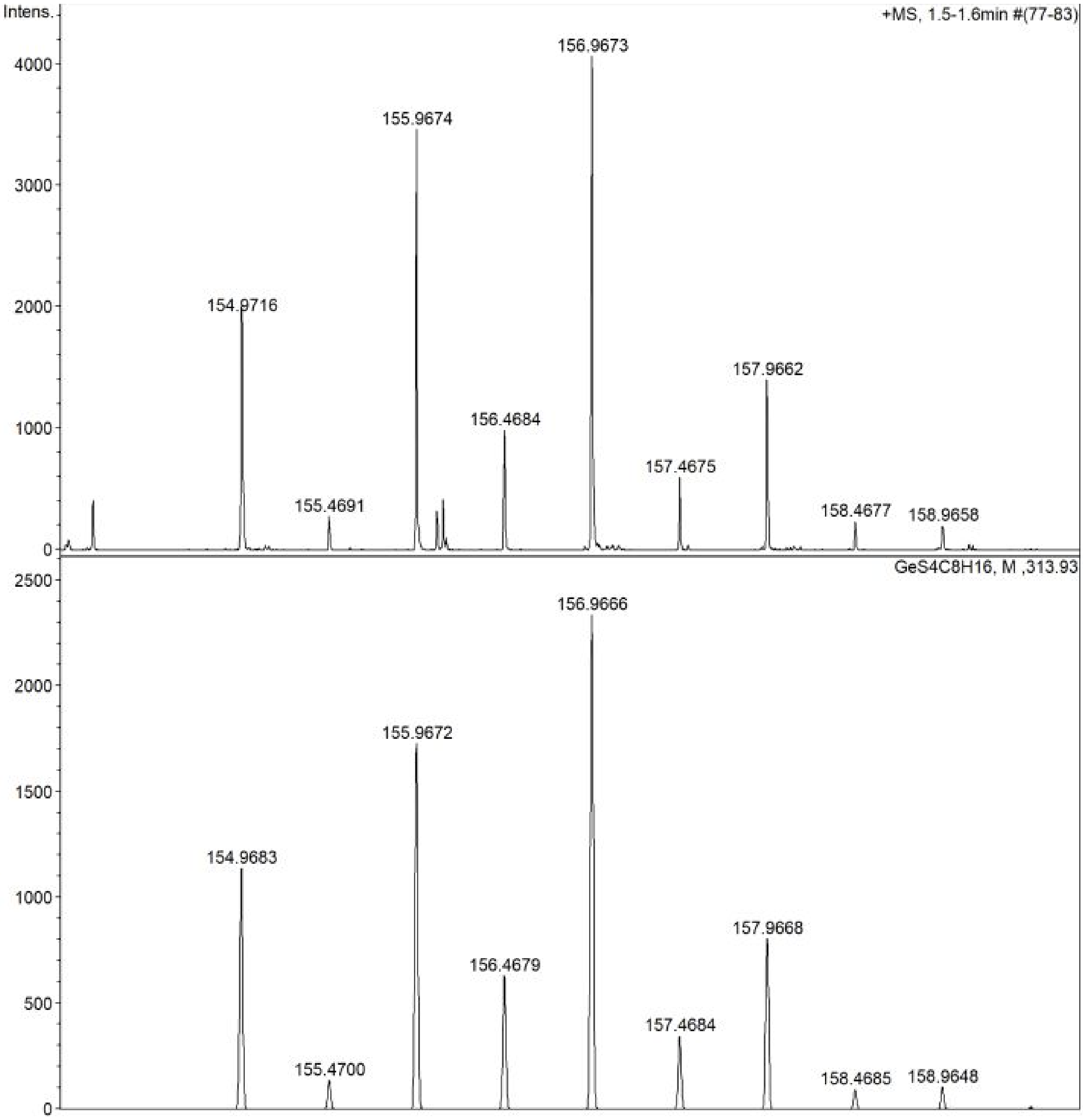

Figure S5 [Sn([12]aneS$_4$)][OTf]$_2$ **(5)**

S5.1 ^{1}H NMR spectrum of [Sn([12]aneS$_4$)][OTf]$_2$ (CD$_3$CN*, 298 K):

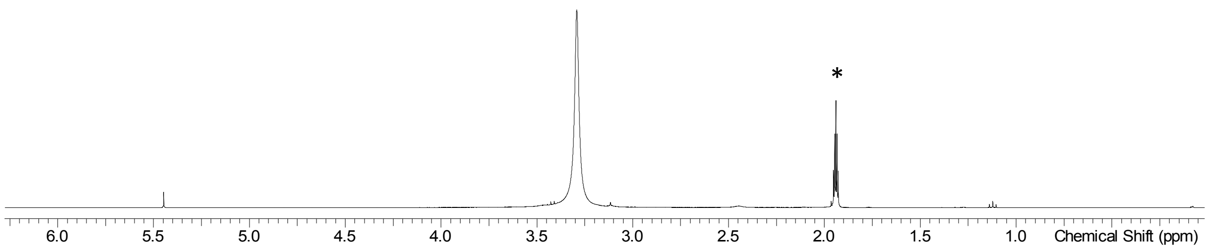

S5.2 ^{13}C{^{1}H} NMR spectrum of [Sn([12]aneS$_4$)][OTf]$_2$ (CD$_3$CN*, 298 K):

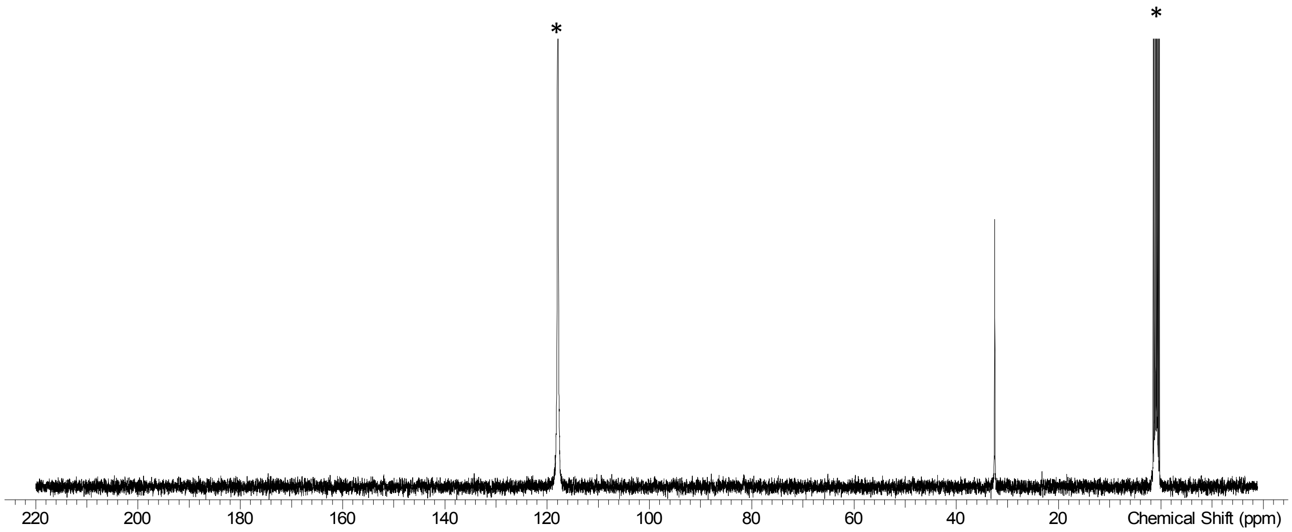

S5.3 ^{19}F{^{1}H} NMR spectrum of [Sn([12]aneS$_4$)][OTf]$_2$ (CD$_3$CN, 298 K):

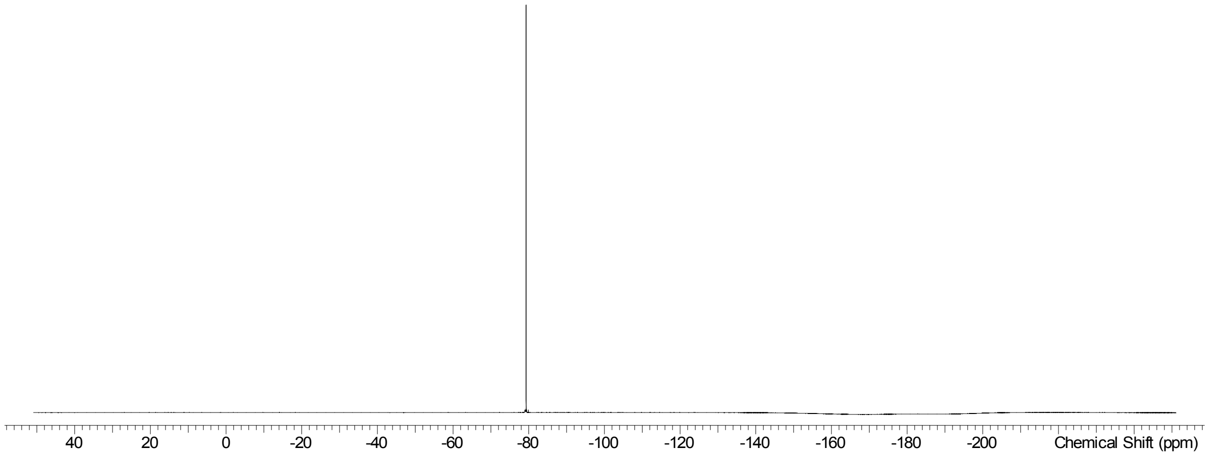

S5.4 $^{119}Sn\{^1H\}$ NMR spectrum of $[Sn([12]aneS_4)][OTf]_2$ (CD_3CN, 298 K):

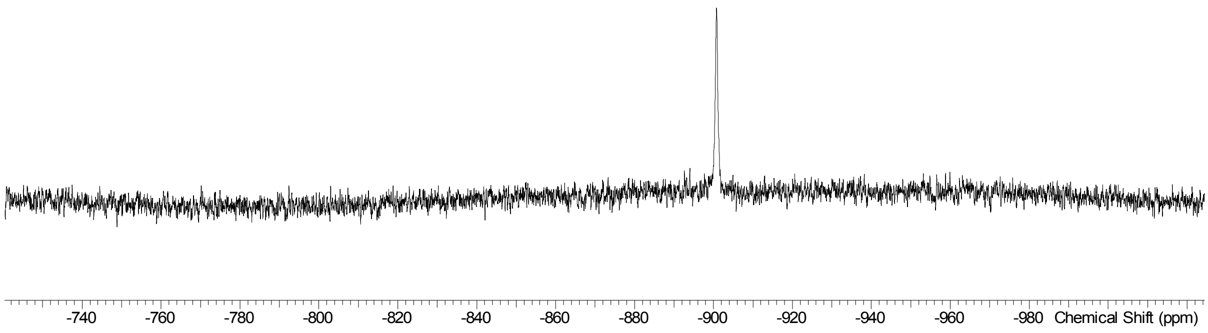

S5.5 IR spectrum of $[Sn([12]aneS_4)][OTf]_2$ (Nujol)

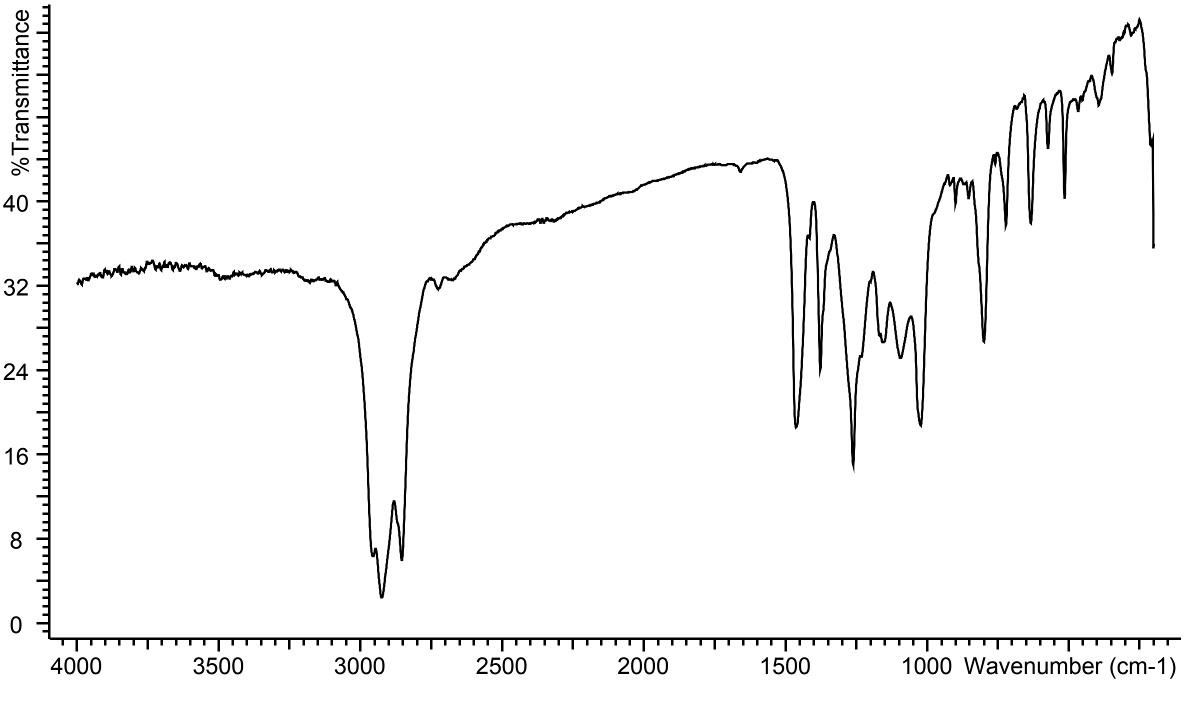

S5.6 HRMS (ESI$^+$, MeCN) (top: experimental; bottom: simulated for $[Sn([12]aneS_4)(OTf)]^+$

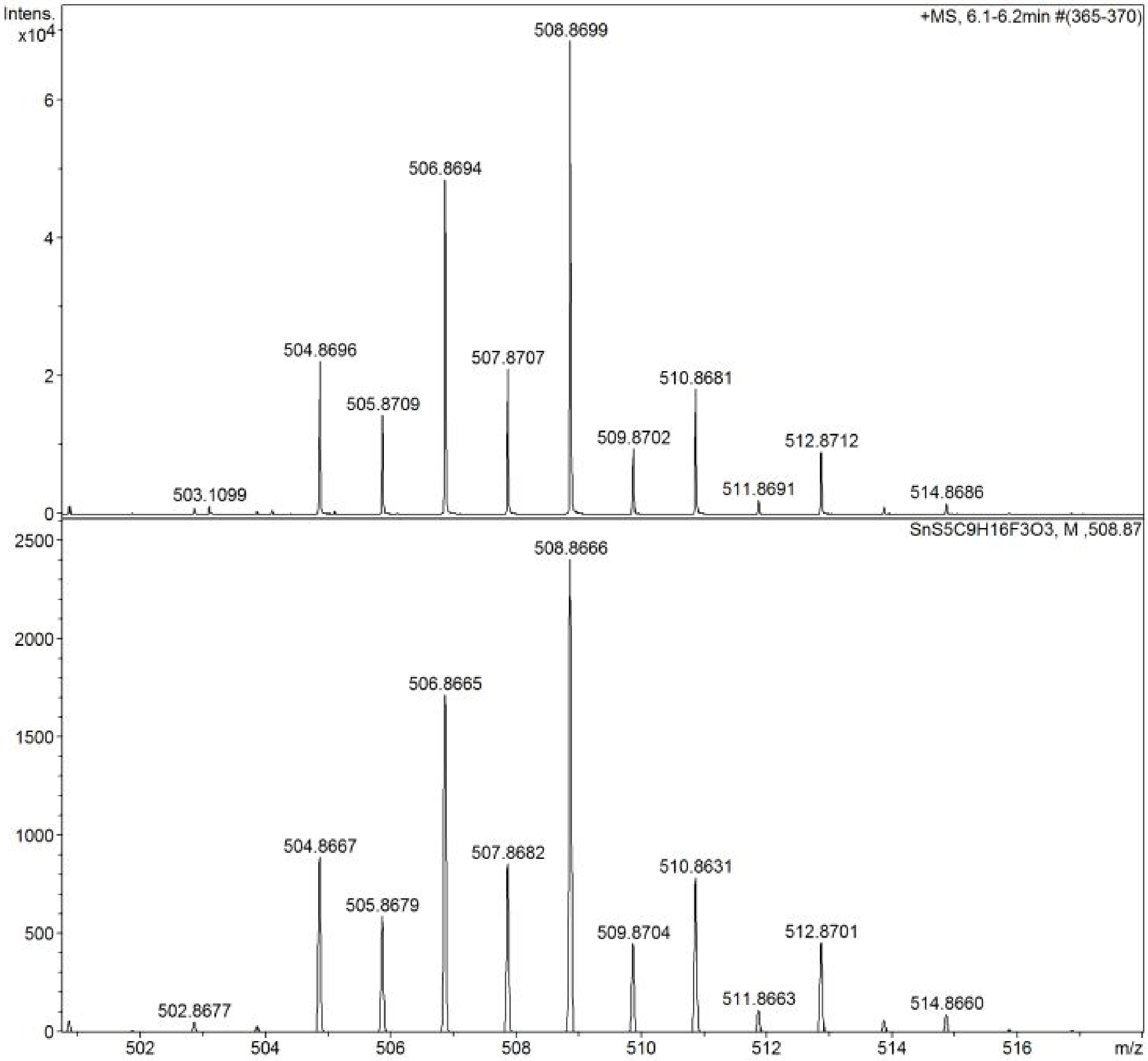

S5.7 HRMS (ESI$^+$, MeCN) (top: experimental; bottom: simulated for $[Sn([12]aneS_4)]^{2+}$

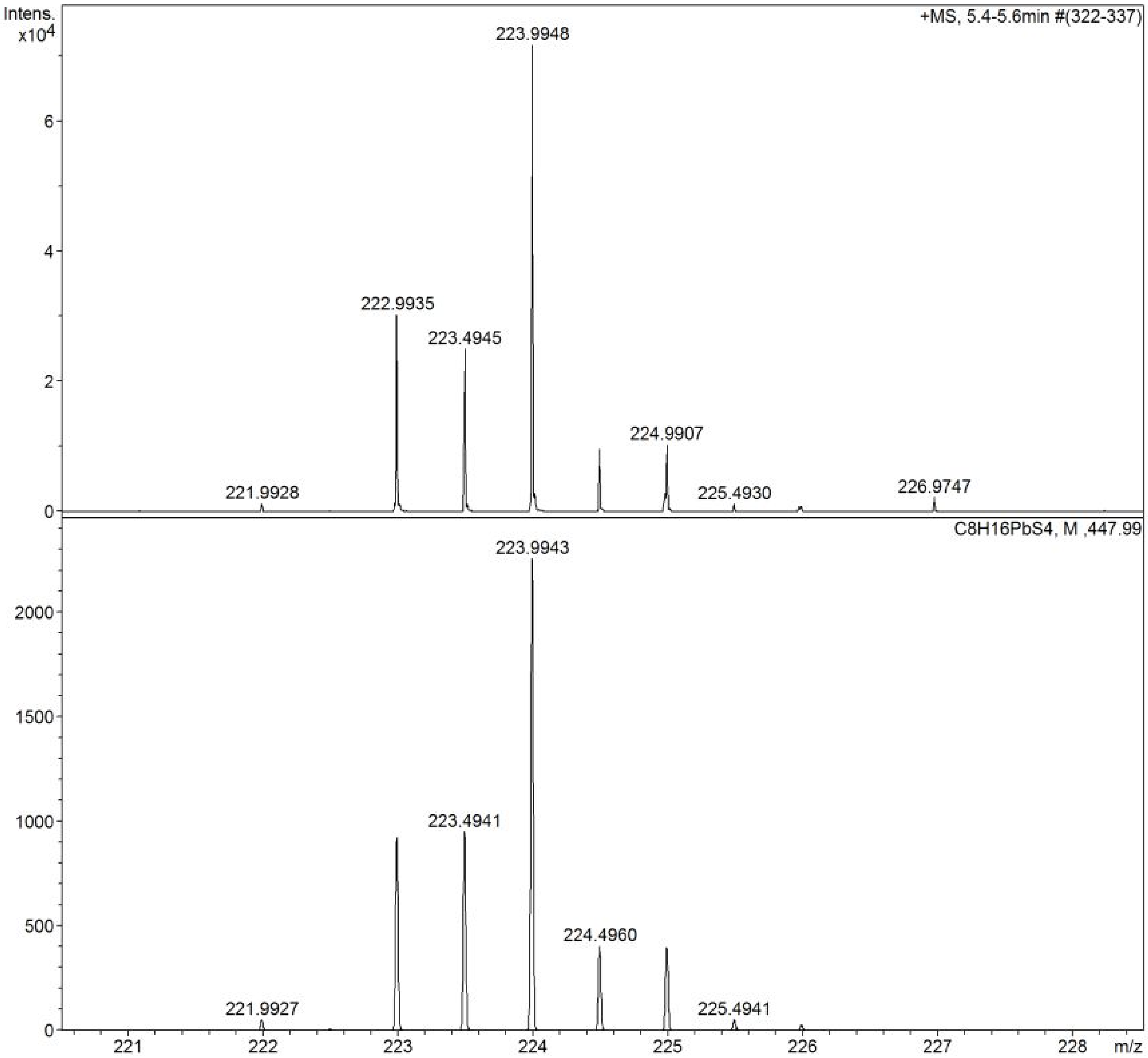

Figure S6 [Pb([12]aneS$_4$)][OTf]$_2$ **(6)**

S6.1 ^{1}H NMR spectrum of [Pb([12]aneS$_4$)][OTf]$_2$ (CD$_3$CN*, 298 K): CH$_2$Cl$_2$^

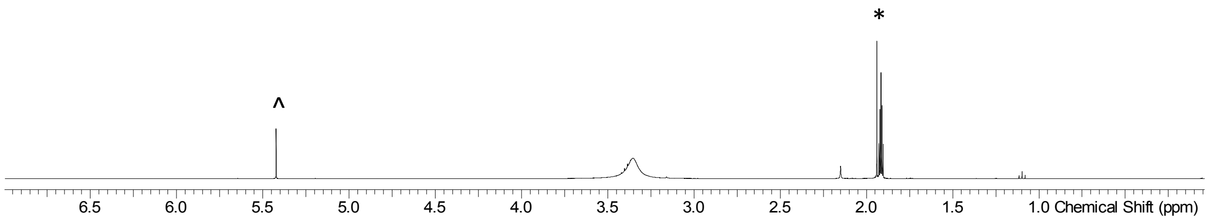

S6.2 ^{13}C{^{1}H} NMR spectrum of [Pb([12]aneS$_4$)][OTf]$_2$ (CD$_3$CN*, 298 K):

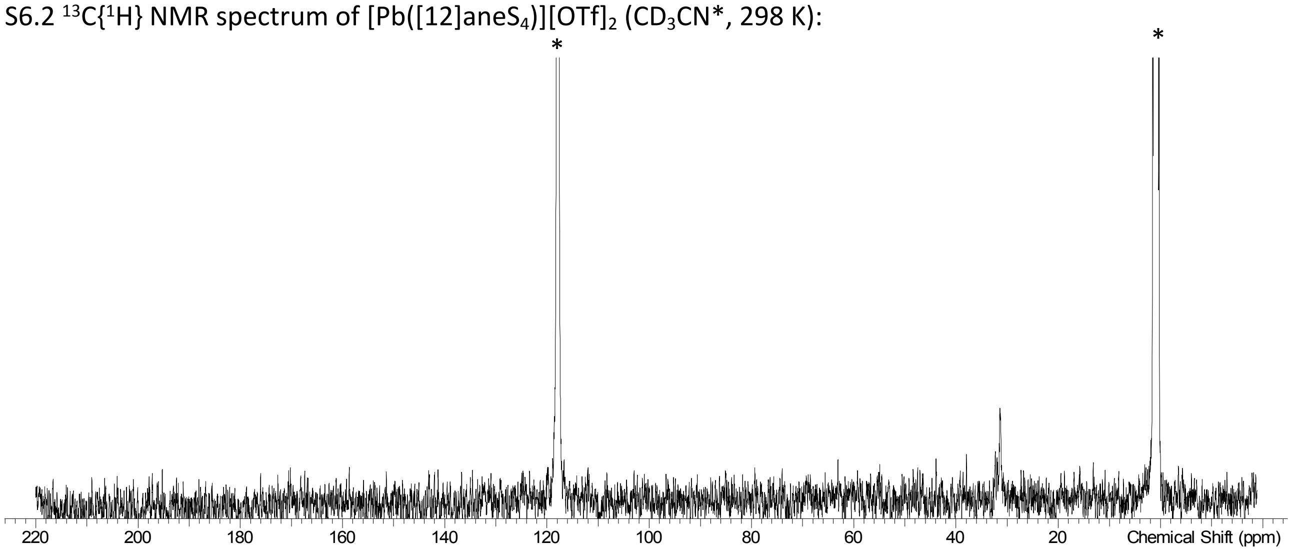

S6.3 ^{19}F{^{1}H} NMR spectrum of [Pb([12]aneS$_4$)][OTf]$_2$ (CD$_3$CN, 298 K):

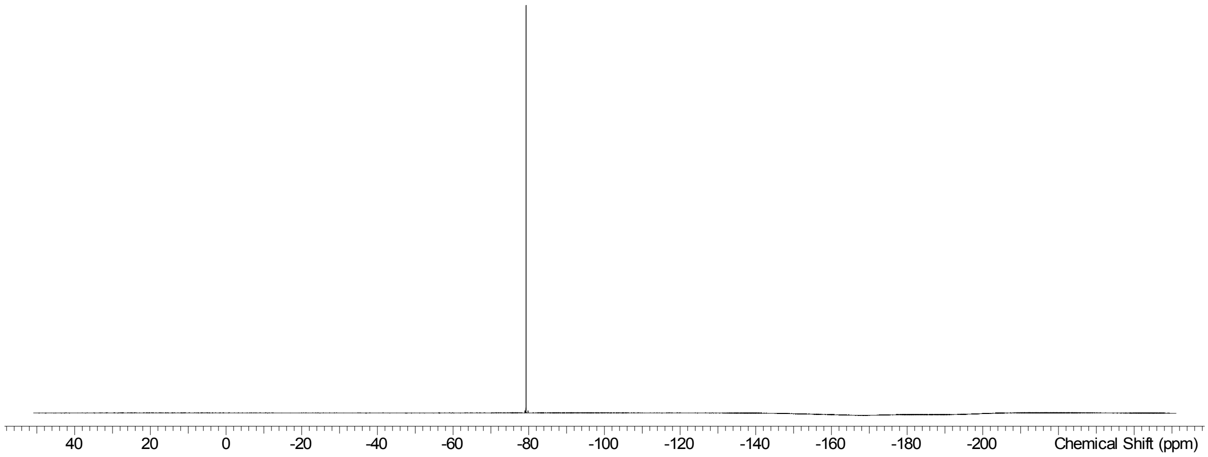

S6.4 IR spectrum of [Pb([12]aneS$_4$)][OTf]$_2$ (Nujol)

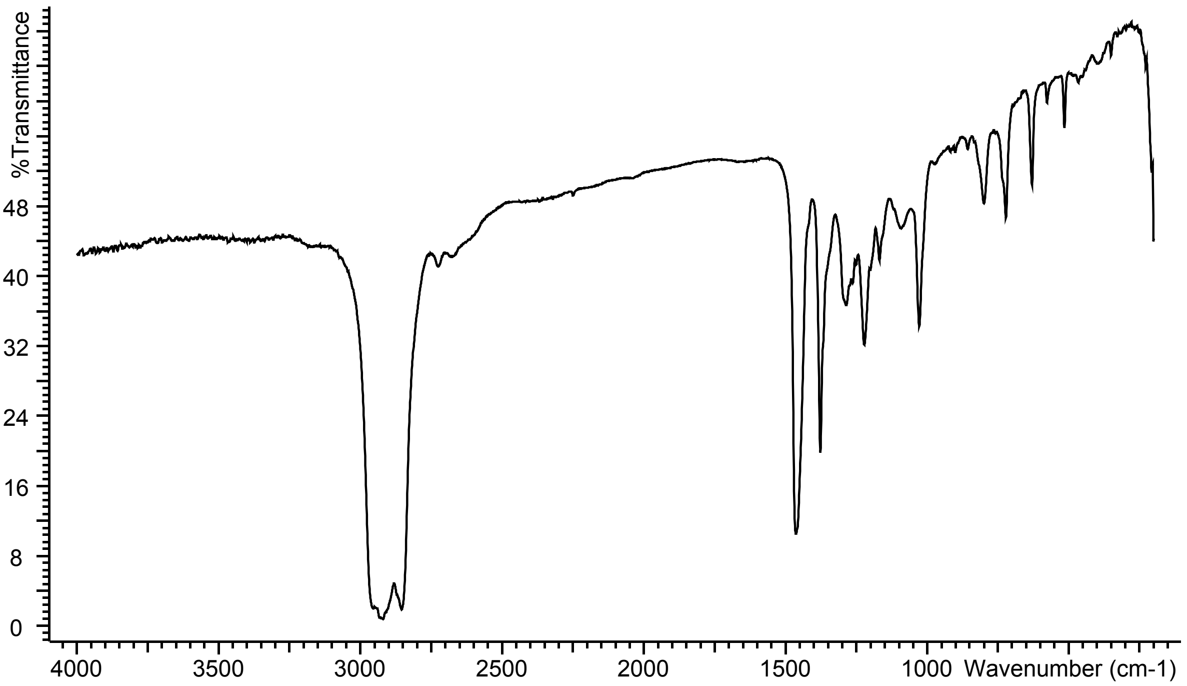

S6.5 HRMS (ESI$^+$, MeCN) (top: experimental; bottom: simulated for $[Pb([12]aneS_4)(OTf)]^+$

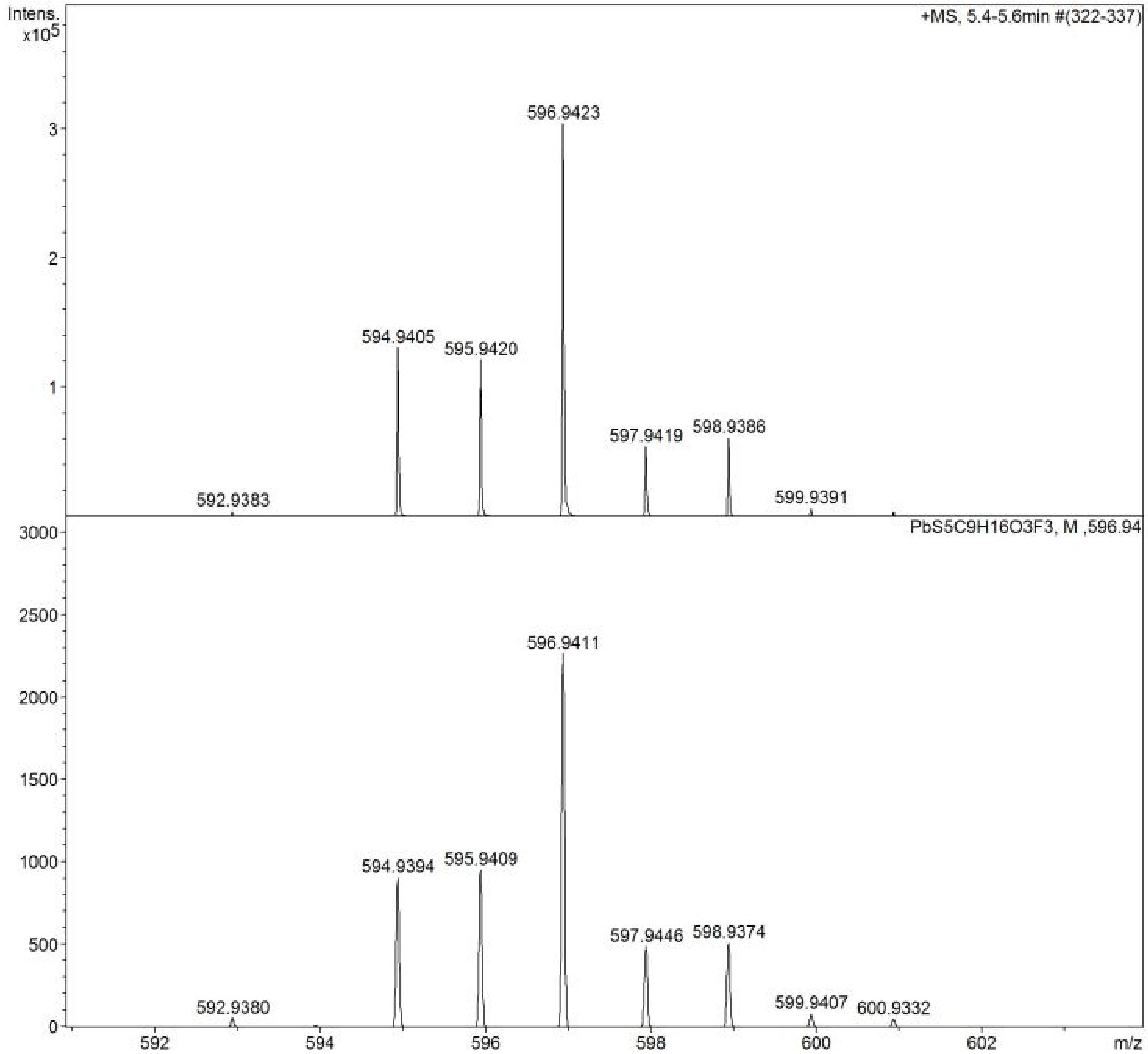

S6.6 HRMS (ESI^+, MeCN) (top: experimental; bottom: simulated for $[Pb([12]aneS_4)]^{2+}$

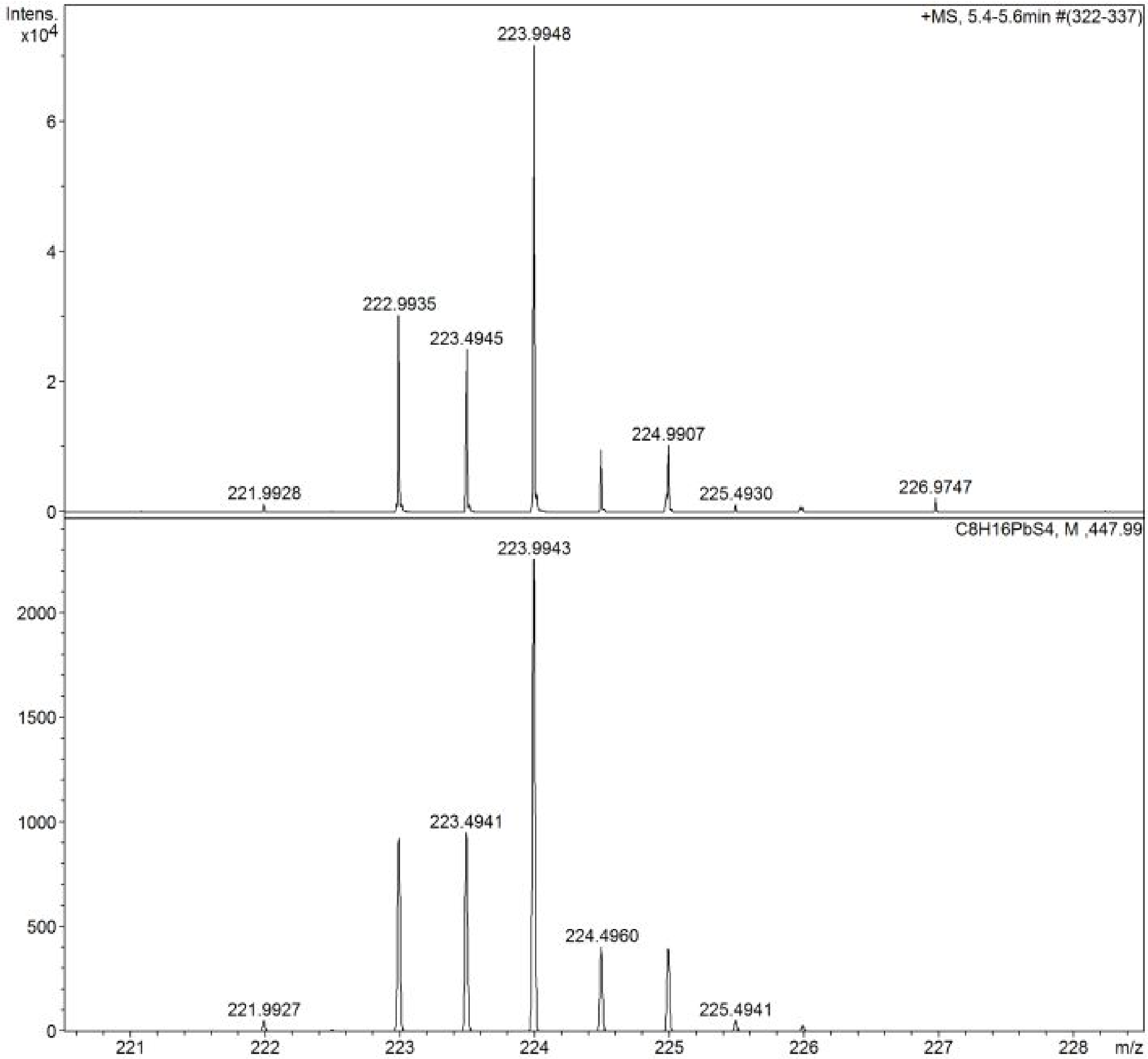

Figure S7 [Ge([24]aneS$_8$)][OTf]$_2$ **(7)**

S7.1 ^{1}H NMR spectrum of [Ge([24]aneS$_8$)][OTf]$_2$ (CD$_2$Cl$_2$^, 298 K): MeCN*

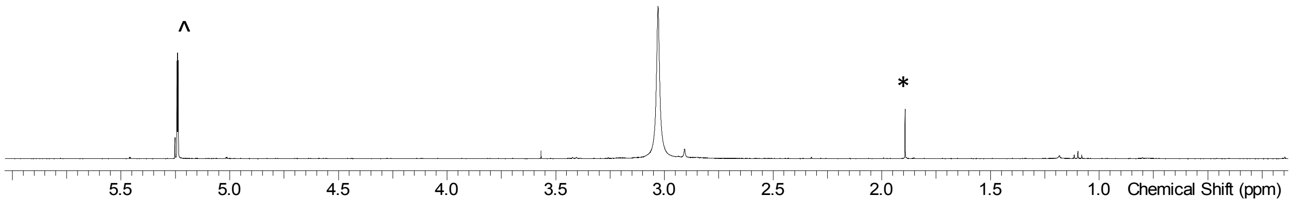

S7.2 ^{13}C{^{1}H} NMR spectrum of [Ge([24]aneS$_8$)][OTf]$_2$ (CD$_2$Cl$_2$^, 298 K):

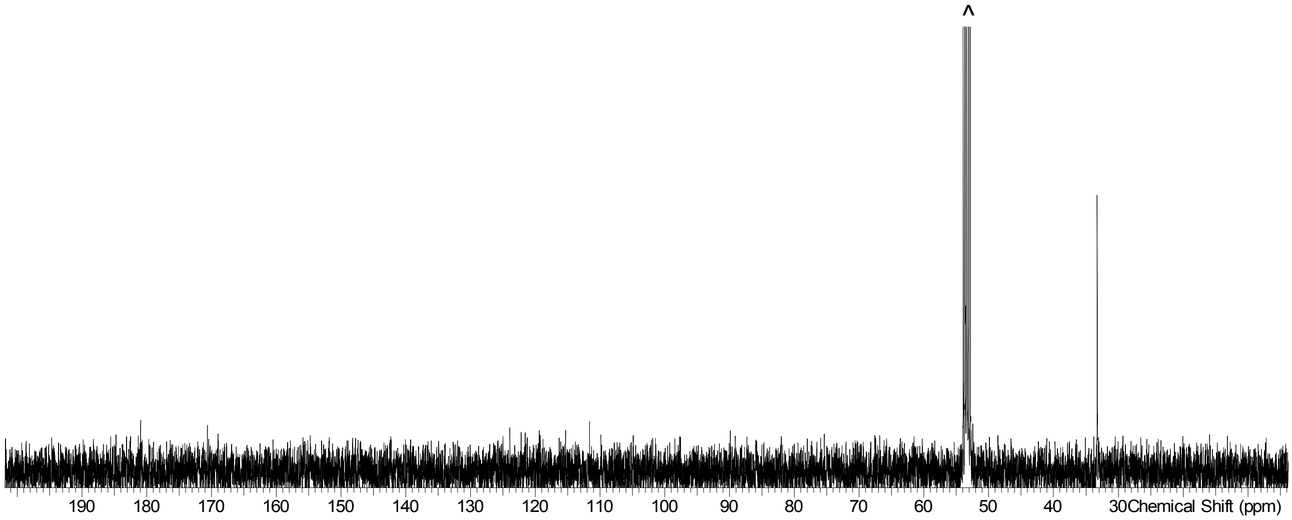

S7.3 ^{19}F{^{1}H} NMR spectrum of [Ge([24]aneS$_8$)][OTf]$_2$ (CD$_2$Cl$_2$, 298 K): broad feature = Teflon from the probe

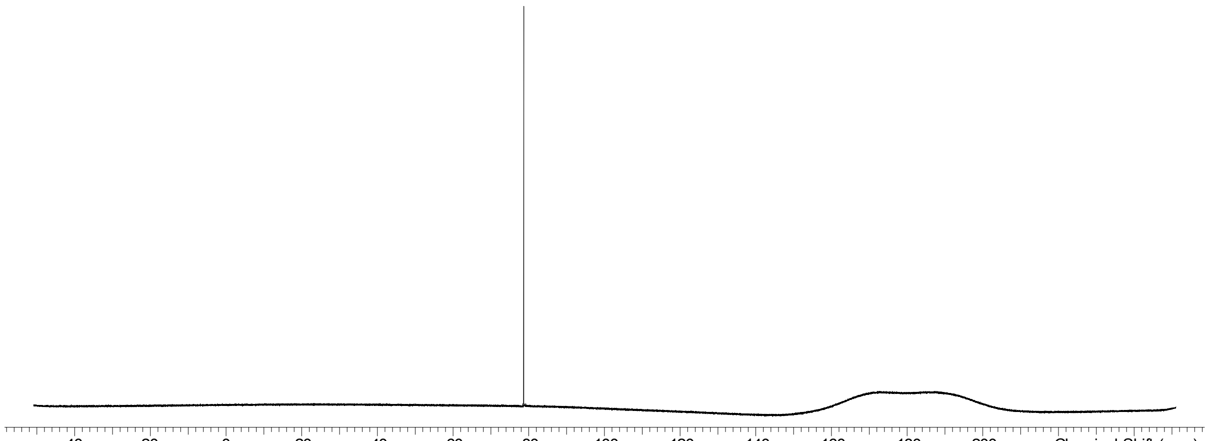

S7.4 HRMS (ESI$^+$, MeCN) (top: experimental; bottom: simulated for $[Ge([24]aneS_8)(OTf)]^+$

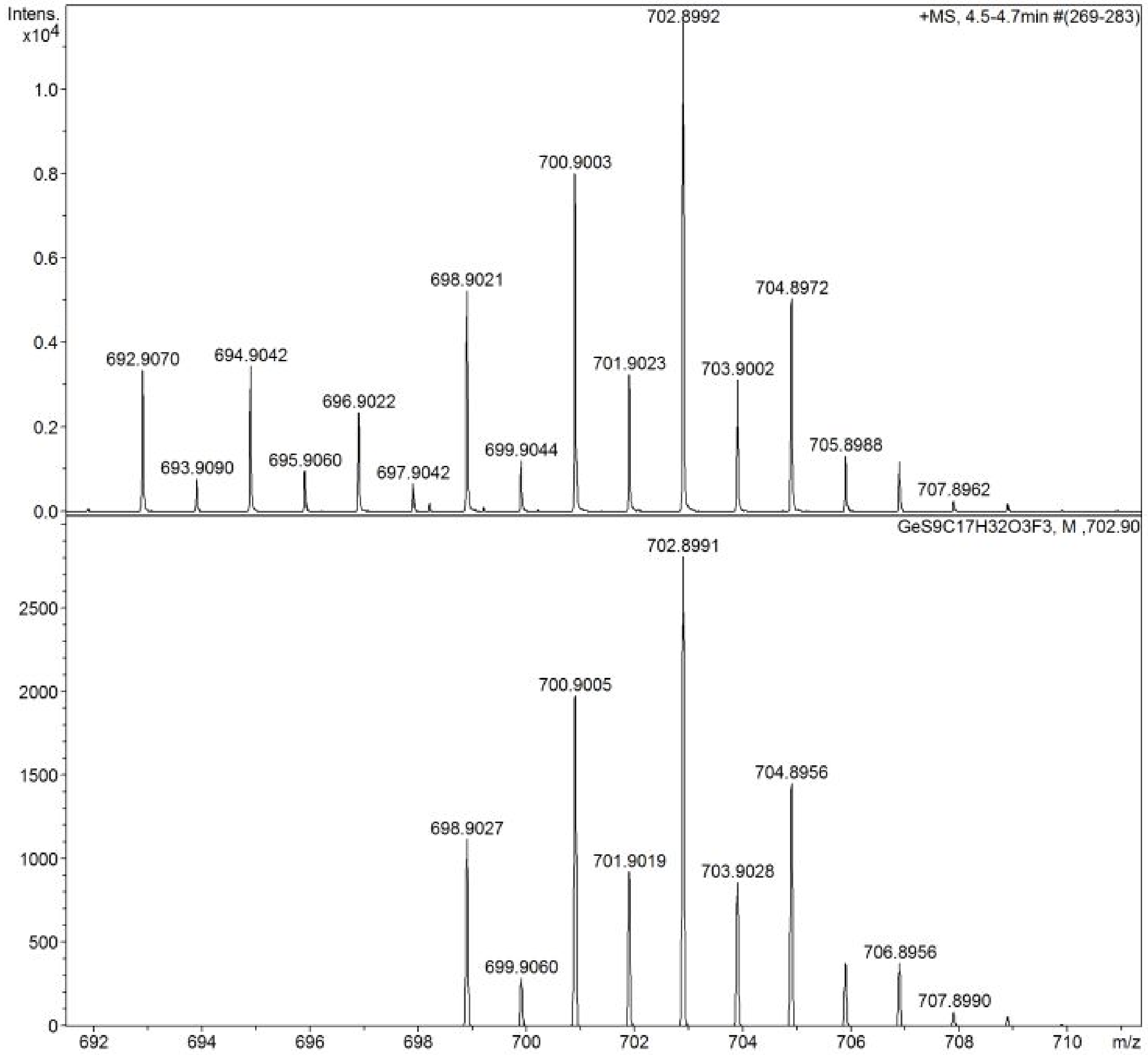

Figure S8 [Sn([24]aneS_8)][OTf]$_2$ **(8)**

S8.1 ^{1}H NMR spectrum of [Sn([24]aneS_8)][OTf]$_2$ (CD_2Cl_2^, 298 K): MeCN*

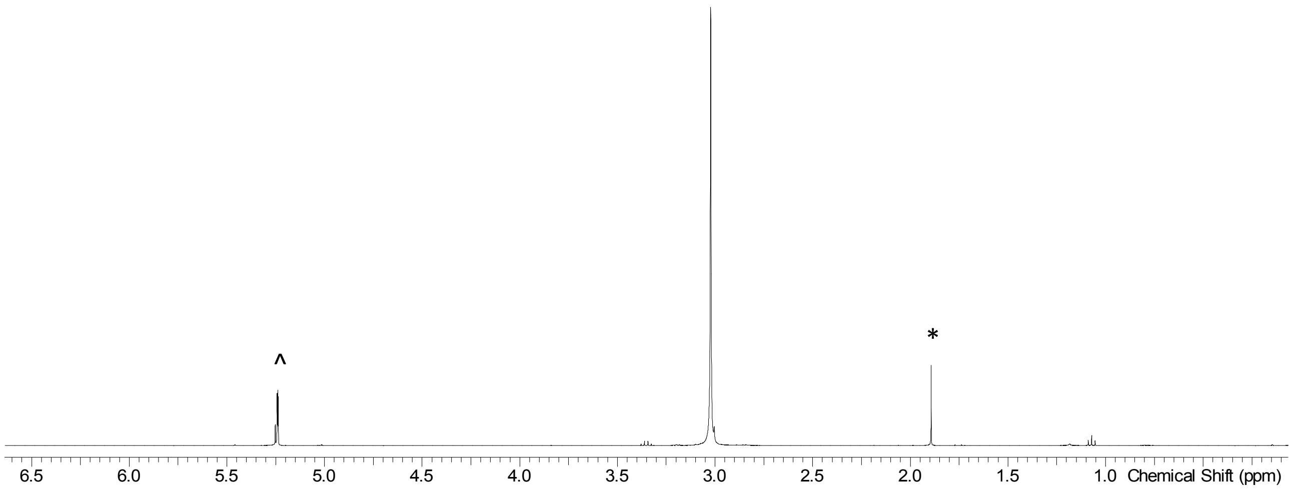

S8.2 ^{13}C{^{1}H} NMR spectrum of [Sn([24]aneS_8)][OTf]$_2$ (CD_2Cl_2^, 298 K):

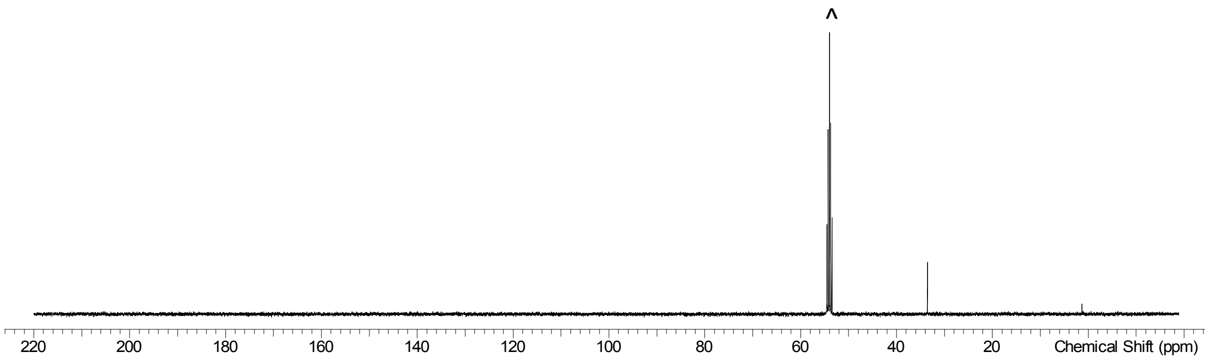

S8.3 ^{19}F{^{1}H} NMR spectrum of [Sn([24]aneS_8)][OTf]$_2$ (CD_2Cl_2, 298 K):

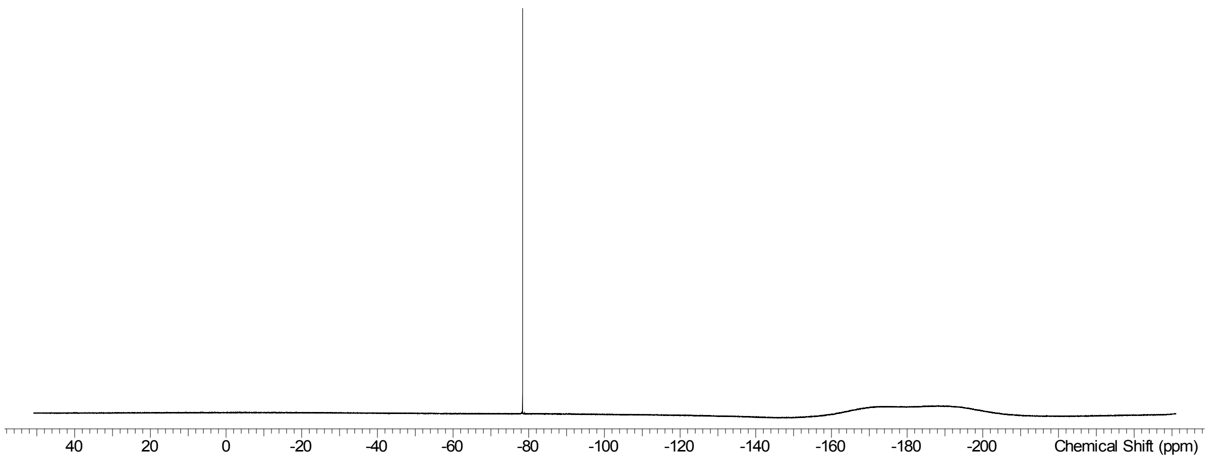

S8.4 $^{119}Sn\{^{1}H\}$ NMR spectrum of $[Sn([24]aneS_8)][OTf]_2$ (CD_2Cl_2, 298 K):

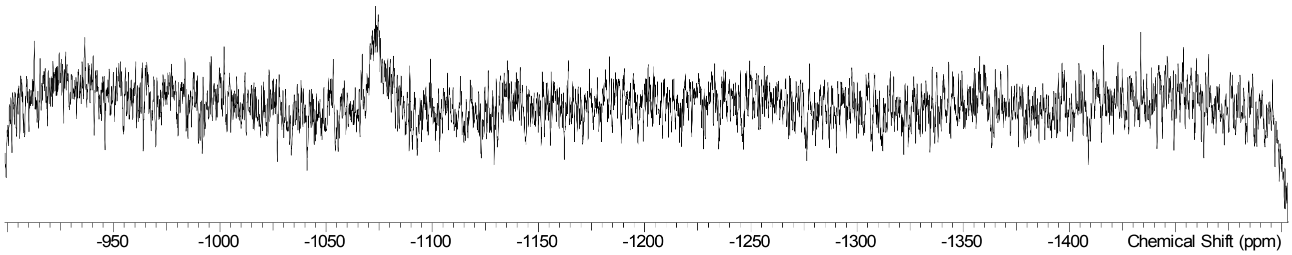

S8.5 IR spectrum of $[Sn([24]aneS_8)][OTf]_2$ (Nujol)

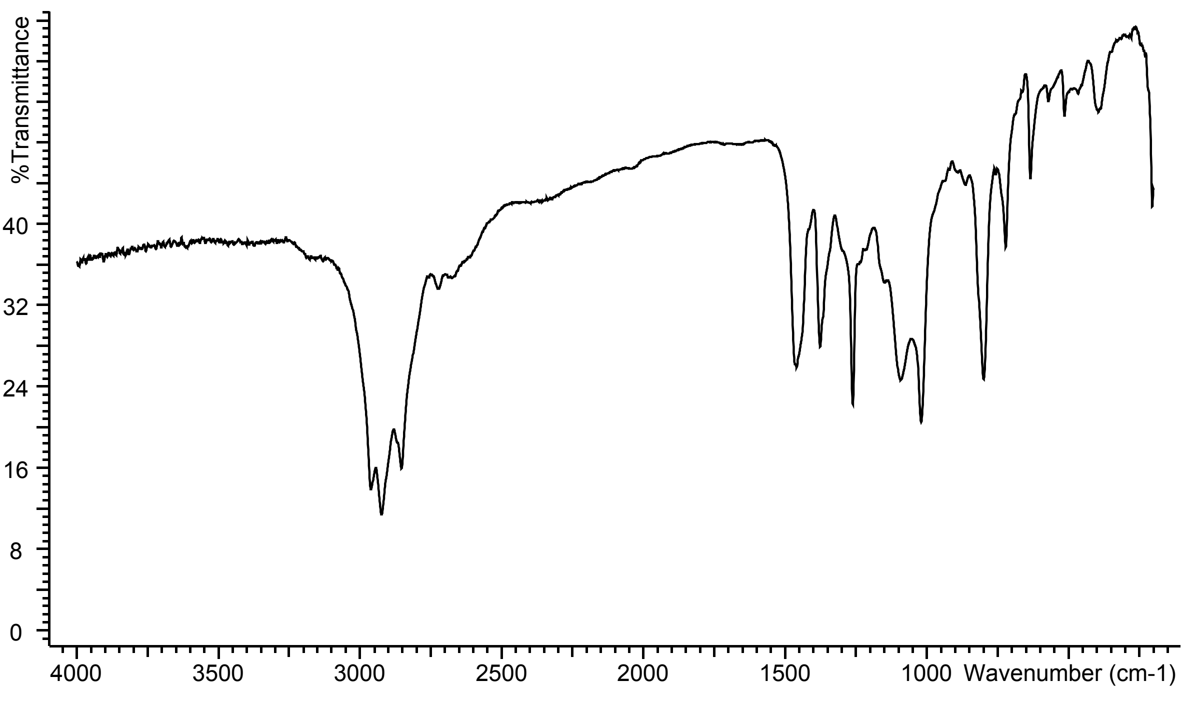

S8.6 HRMS (ESI^+, MeCN) (top: experimental; bottom: simulated for $[Sn([24]aneS_8)(OTf)]^+$

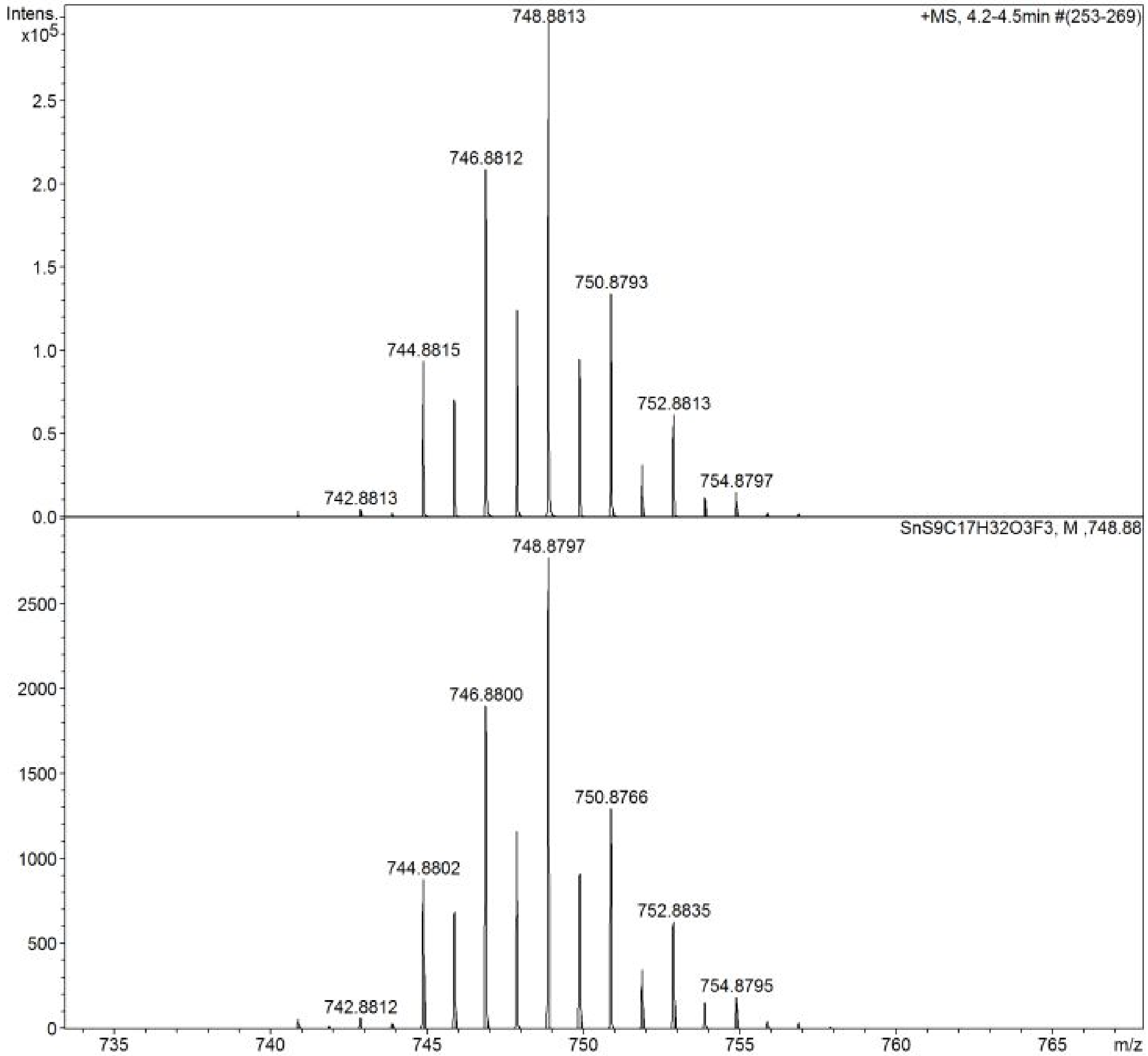

S8.7 HRMS (ESI+, MeCN) (top: experimental; bottom: simulated for $[Sn([24]aneS_8)]^{2+}$

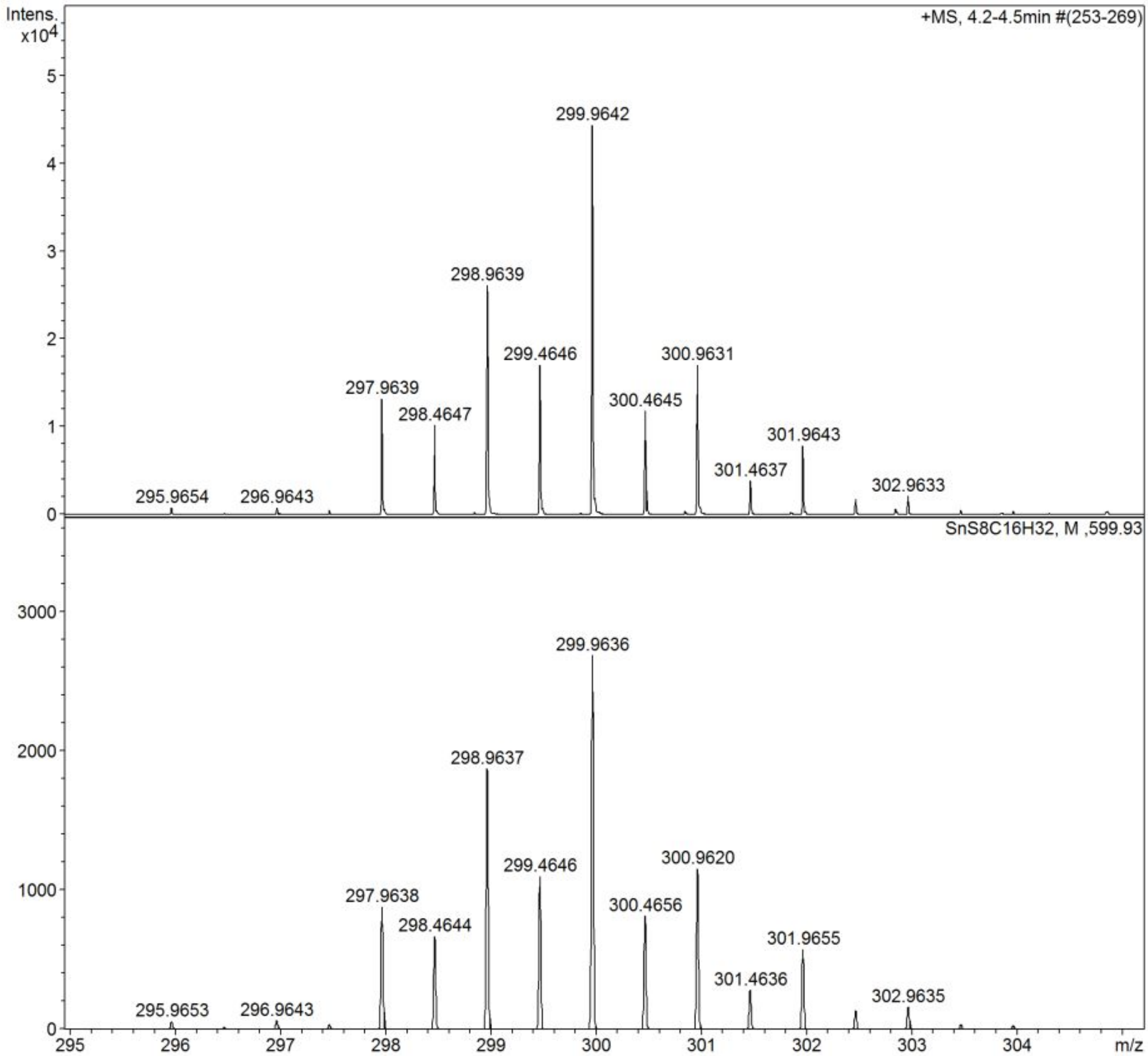

Figure S9 [Pb([24]aneS$_8$)][OTf]$_2$ **(9)**

S9.1 ^{1}H NMR spectrum of [Pb([24]aneS$_8$)][OTf]$_2$ (CD$_2$Cl$_2$^, 298 K): MeCN*

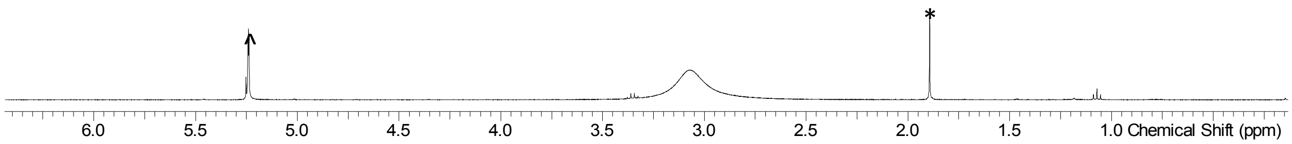

S9.2 ^{13}C{^{1}H} NMR spectrum of [Pb([24]aneS$_8$)][OTf]$_2$ (CD$_2$Cl$_2$^, 298 K):

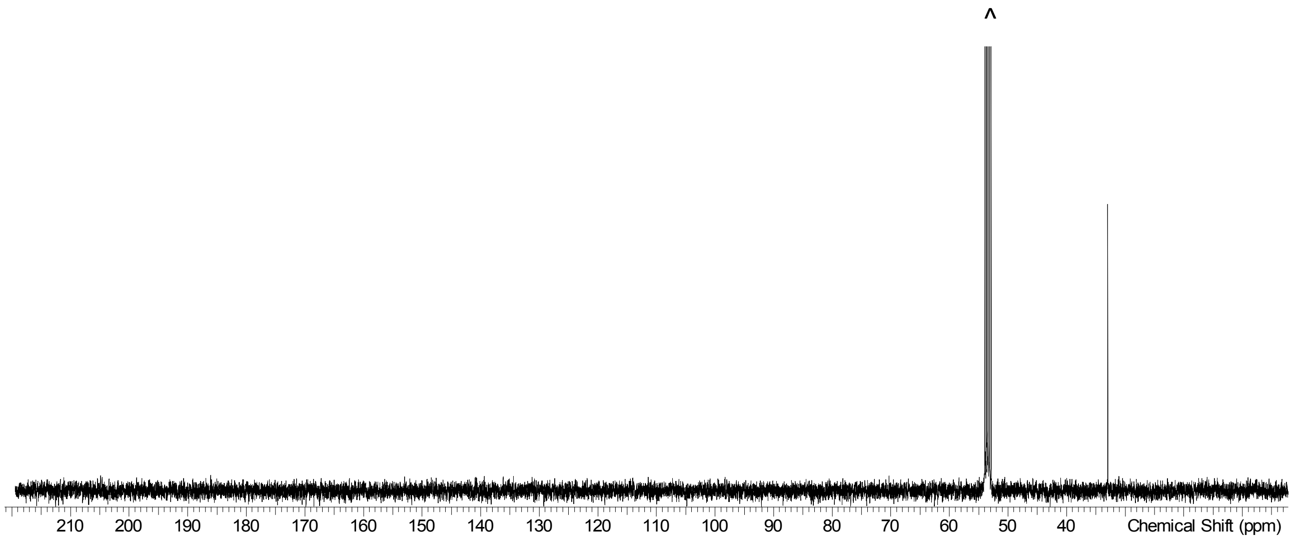

S9.3 ^{19}F{^{1}H} NMR spectrum of [Pb([24]aneS$_8$)][OTf]$_2$ (CD$_2$Cl$_2$, 298 K): the broad feature is from the Telfon in the probe

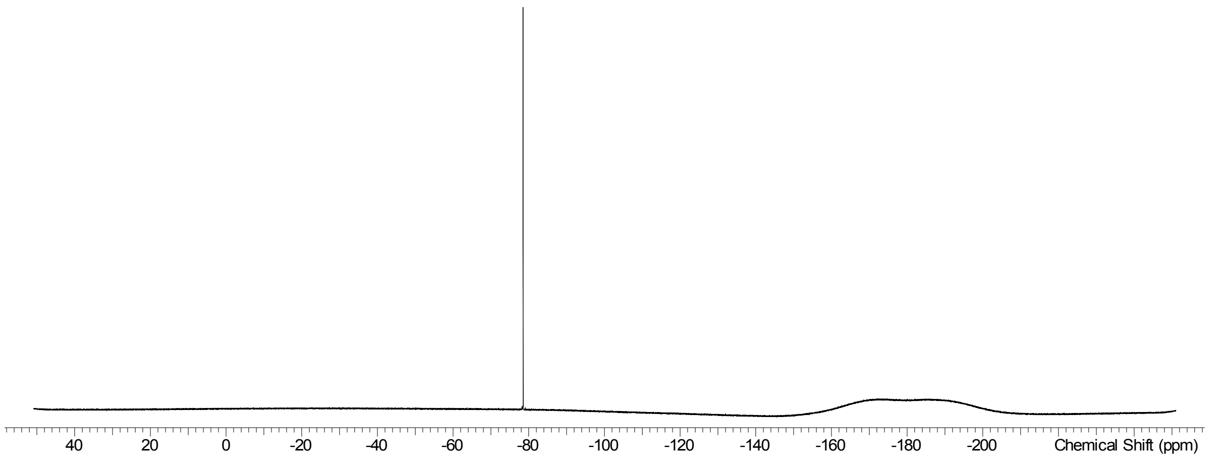

S9.4 HRMS (ESI$^+$, MeCN) (top: experimental; bottom: simulated for $[Pb([24]aneS_8)(OTf)]^+$

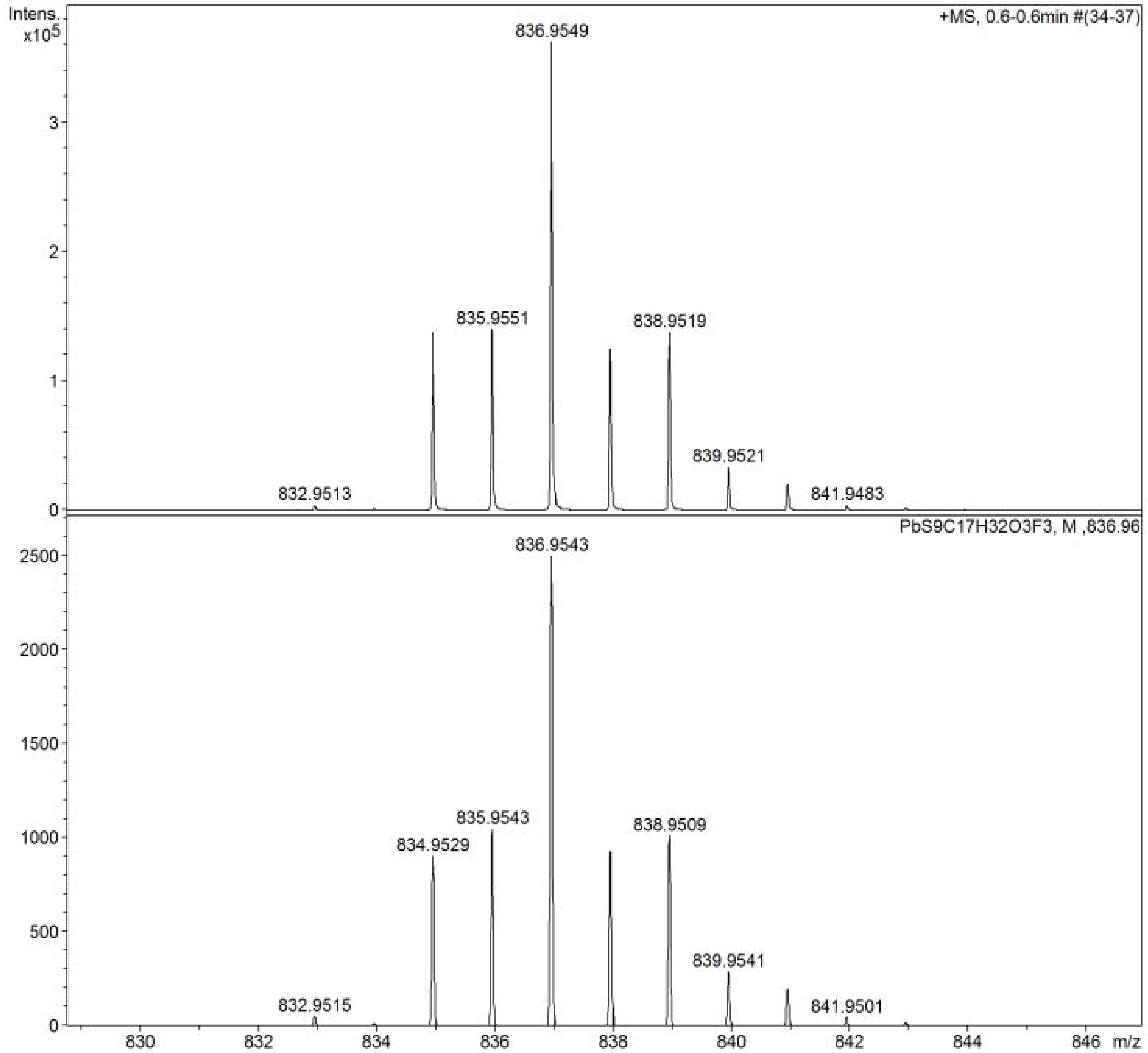

S9.5 HRMS (ESI^+, MeCN) (top: experimental; bottom: simulated for $[Pb([24]aneS_8)]^{2+}$

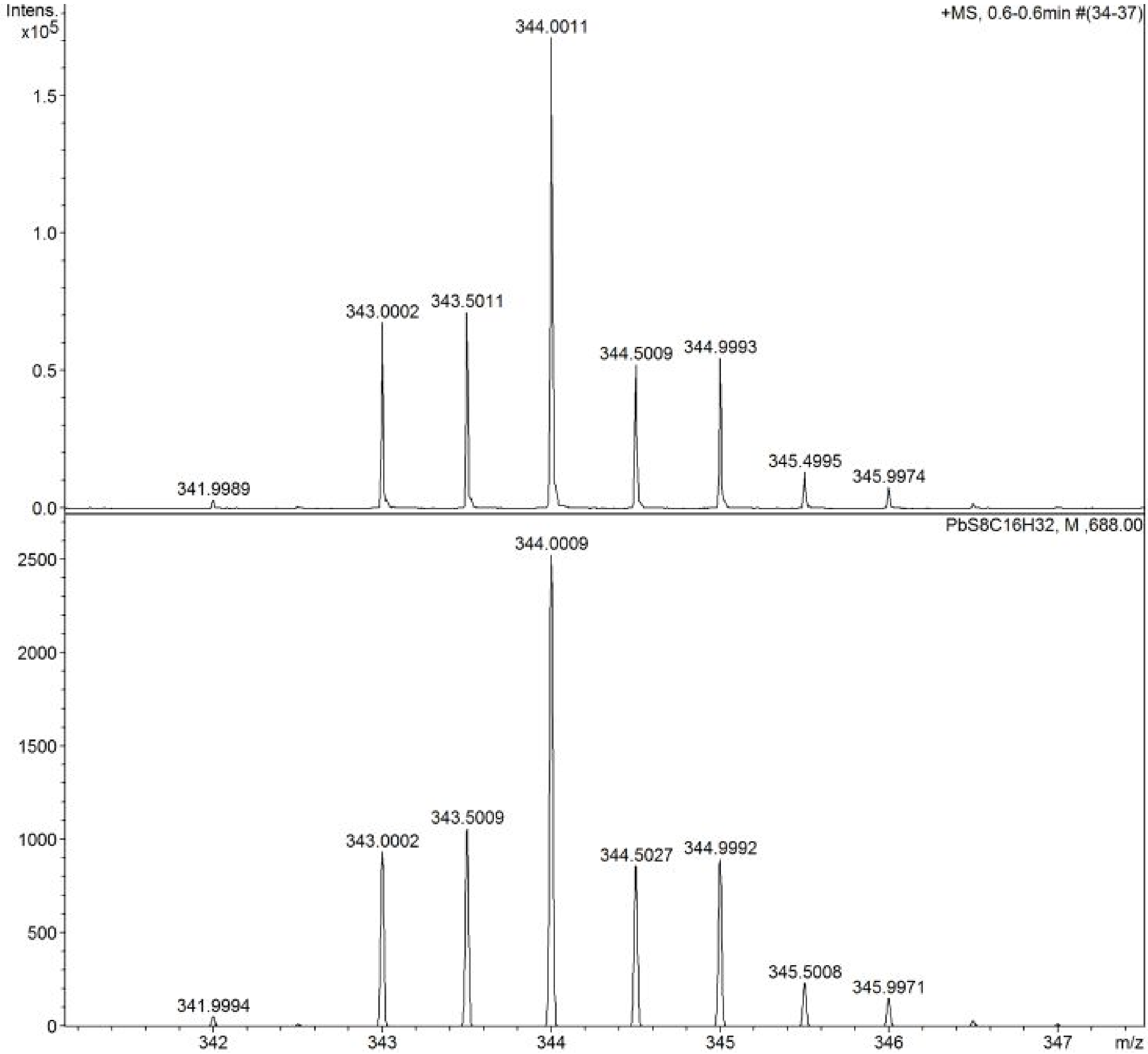

Figure S10 Representations of the frontier orbitals determined from DFT calculations

$[Ge([9]aneS_3)]^{2+}$

**HOMO (-15.74 eV)**	**LUMO (-10.04 eV)**
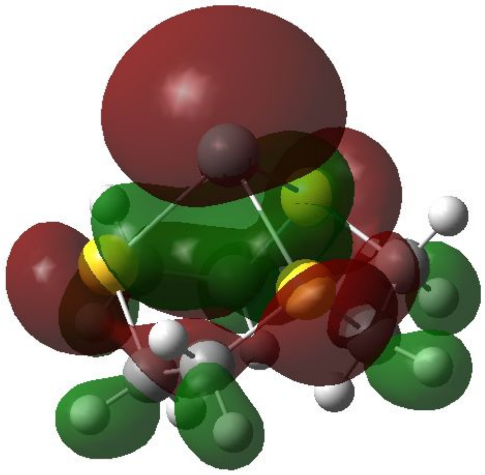	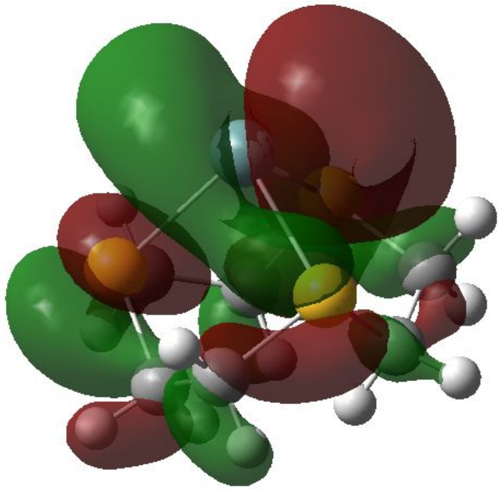
**HOMO-1 (-16.38 eV)**	**LUMO+1 (-10.04 eV)**
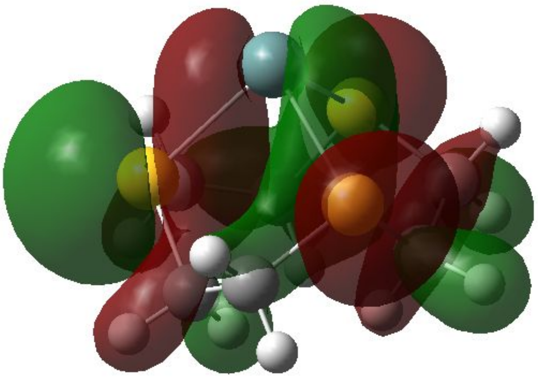	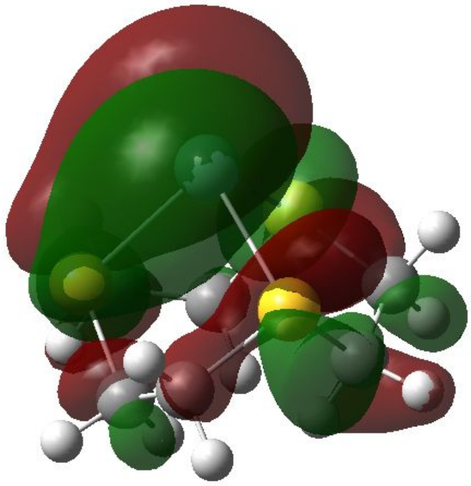
**HOMO-2 (-16.38 eV)**	**LUMO+2 (-9.14 eV)**
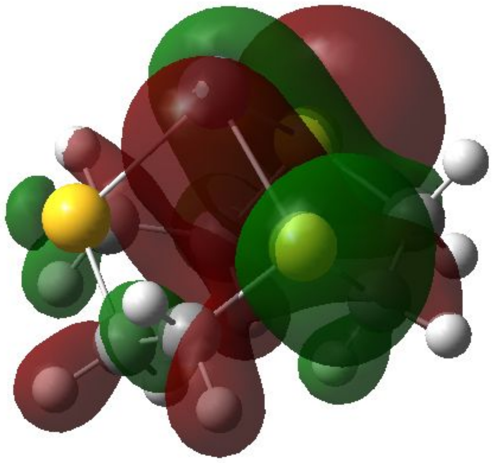	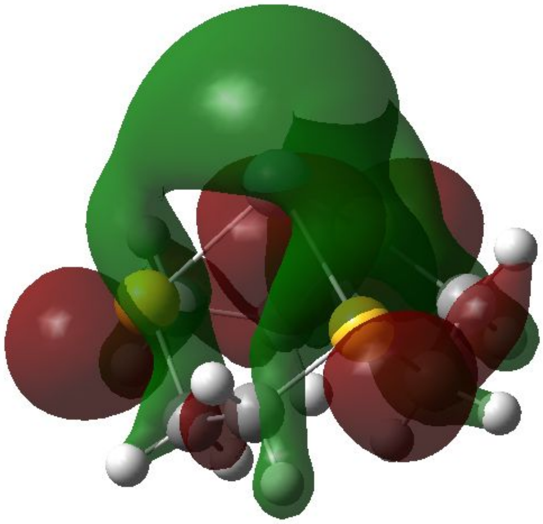

[Sn([9]aneS$_3$)]$^{2+}$

HOMO (-15.45 eV)	LUMO (-10.15 eV)
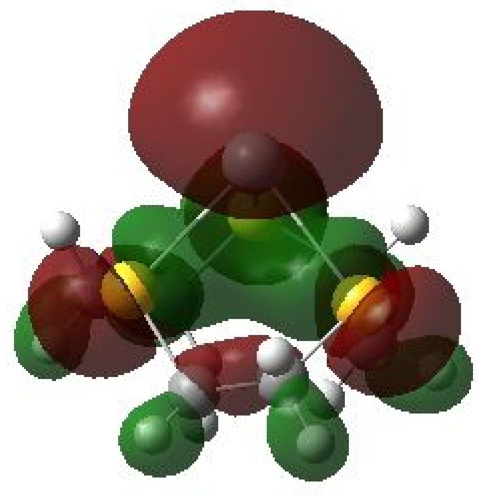	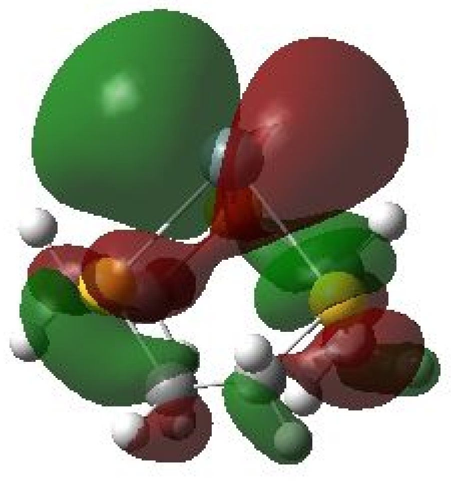
**HOMO-1 (-16.05 eV)**	**LUMO+1 (-10.15 eV)**
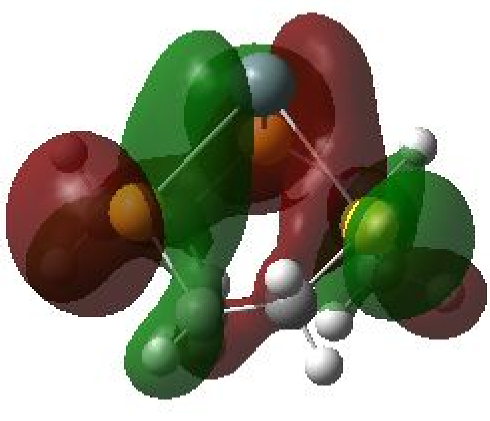	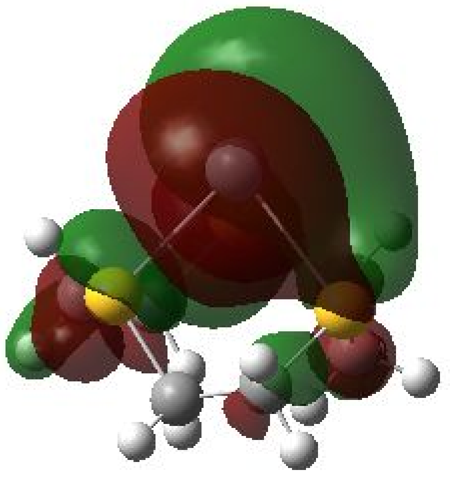
**HOMO-2 (-16.05 eV)**	**LUMO+2 (-8.87 eV)**
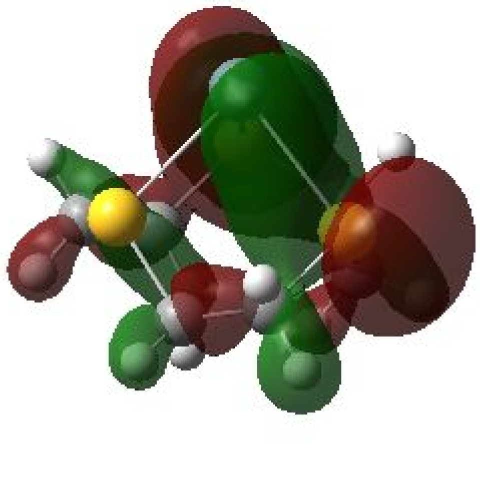	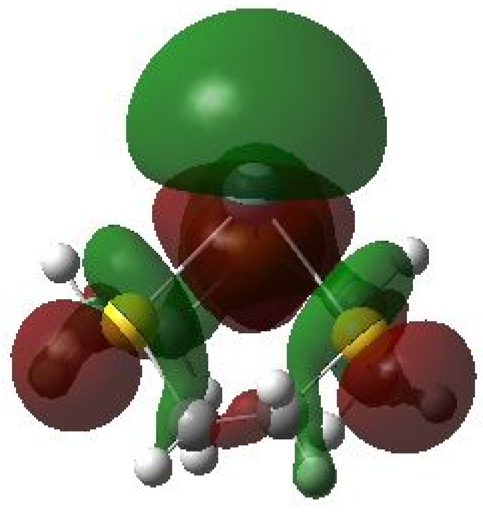

[Pb([9]aneS$_3$)]$^{2+}$

HOMO (-15.66 eV)	LUMO (-10.04 eV)
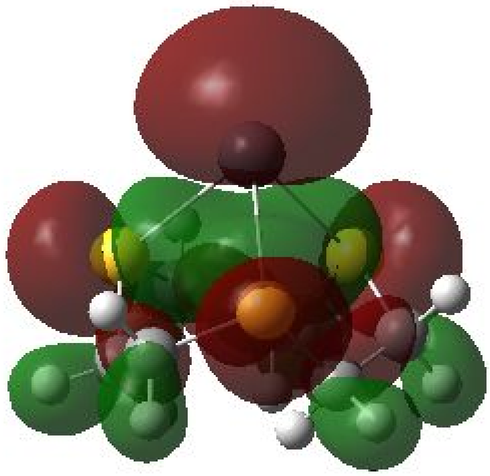	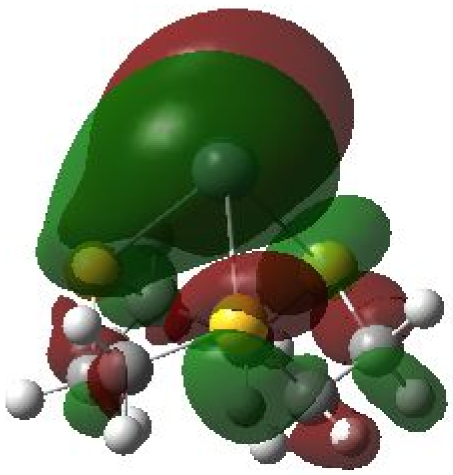
**HOMO-1 (-15.82 eV)**	**LUMO+1 (-10.04 eV)**
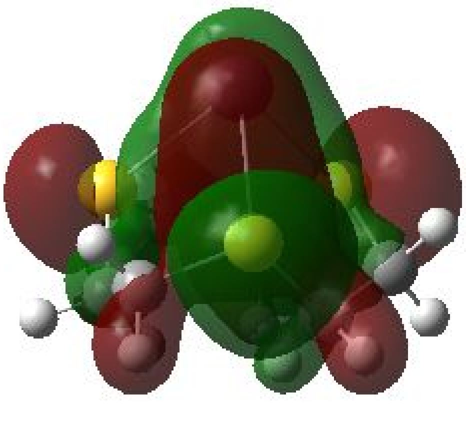	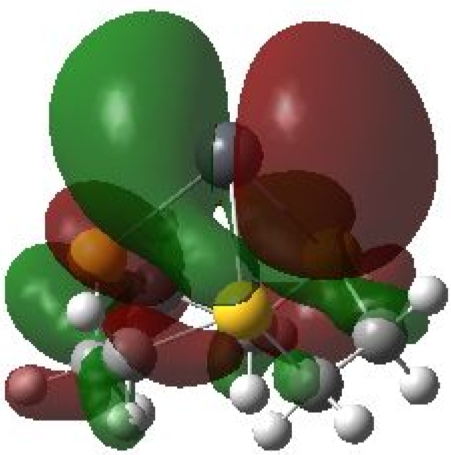
**HOMO-2 (-15.82 eV)**	**LUMO+2 (-8.90 eV)**
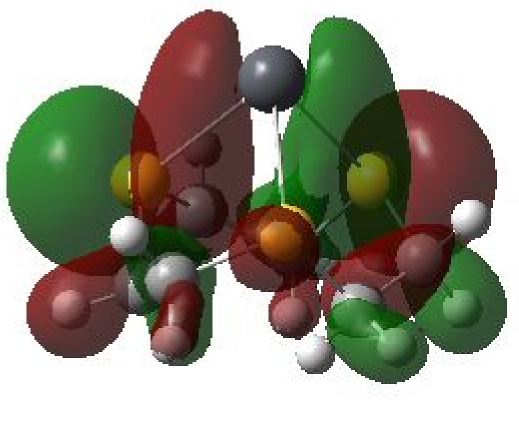	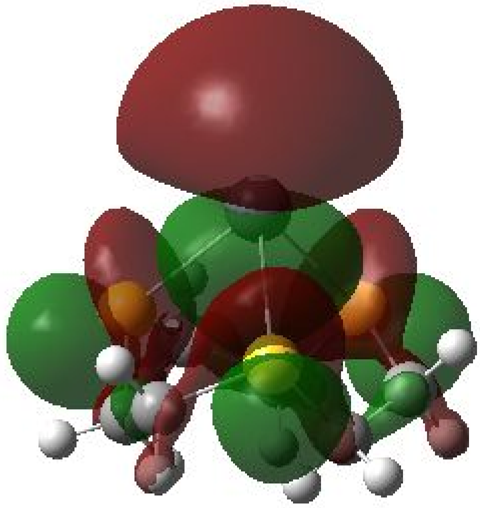

[Ge([12]aneS$_4$)]$^{2+}$

HOMO (-14.04 eV)	LUMO (-9.20 eV)
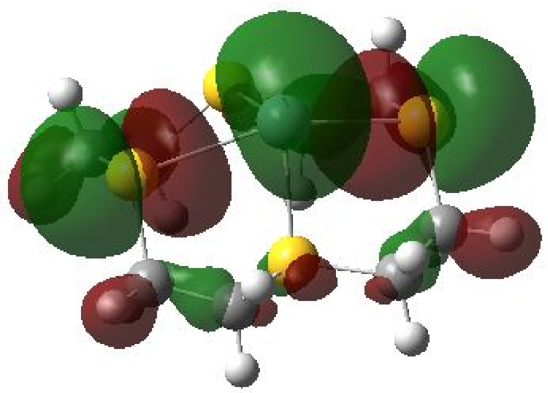	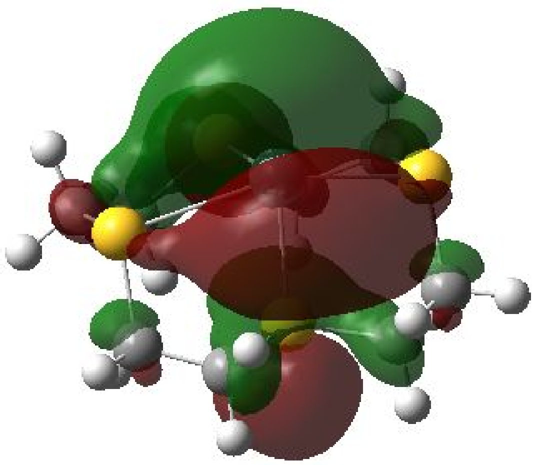
**HOMO-1 (-15.19 eV)**	**LUMO+1 (-8.54 eV)**
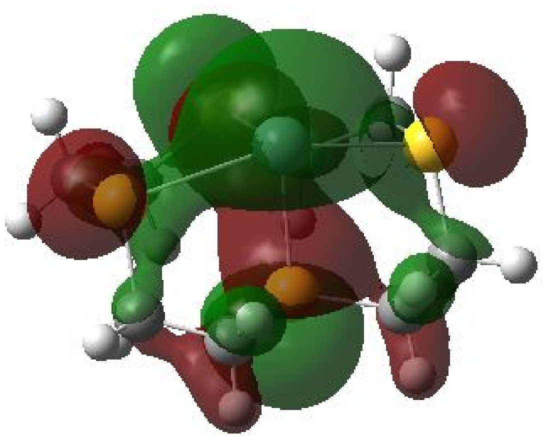	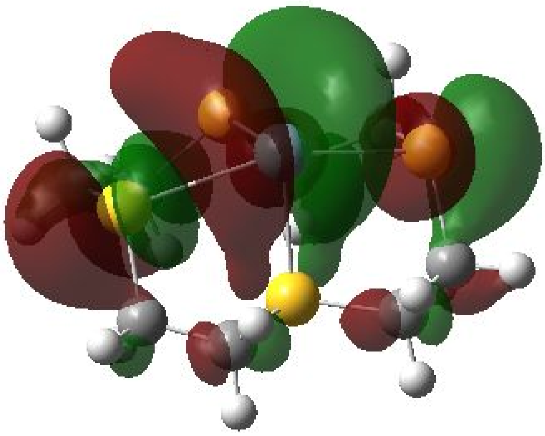
**HOMO-2 (-15.28 eV)**	**LUMO+2 (-8.38 eV)**
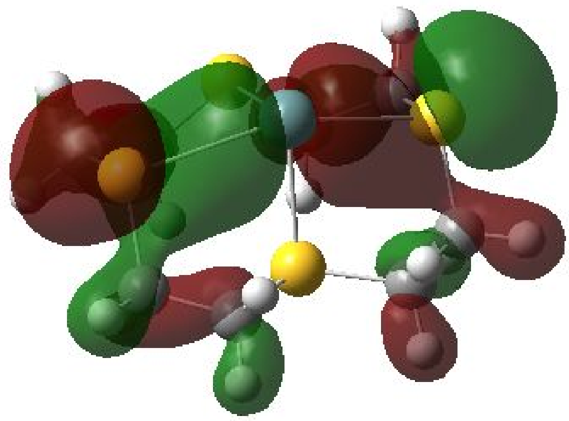	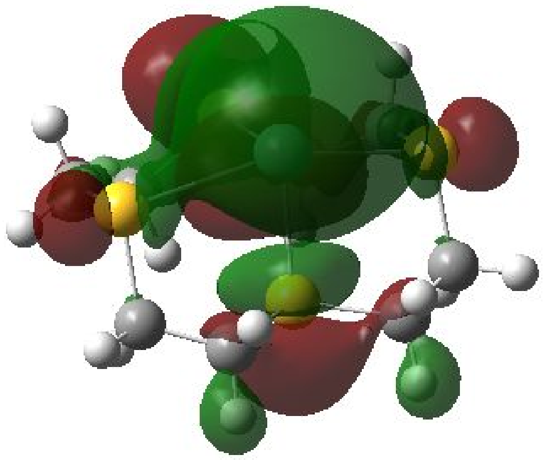
**HOMO-3 (-15.63 eV)**	**LUMO+3 (-7.85 eV)**

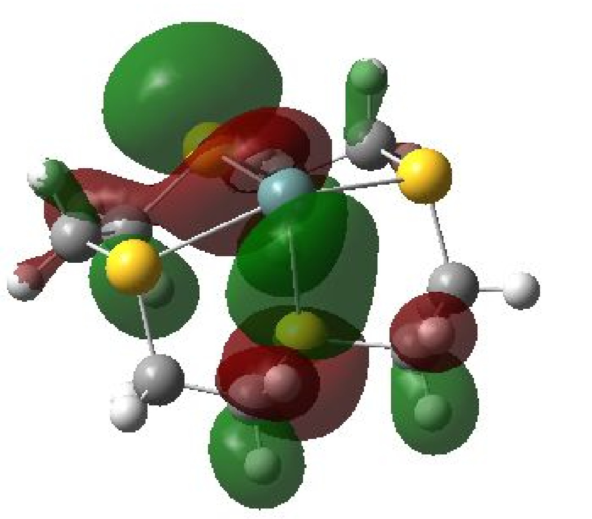	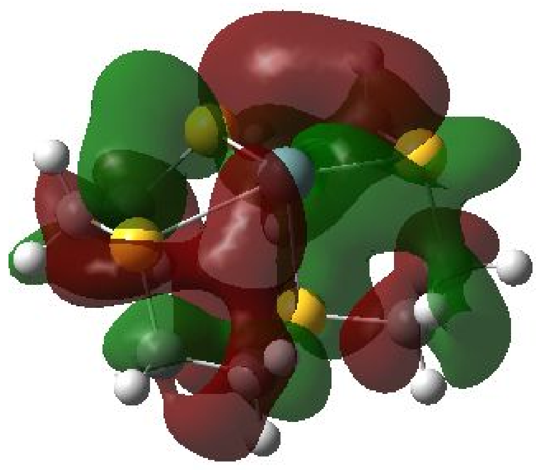

[Sn([12]aneS$_4$)]$^{2+}$

HOMO (-14.40 eV)	LUMO (-9.15 eV)
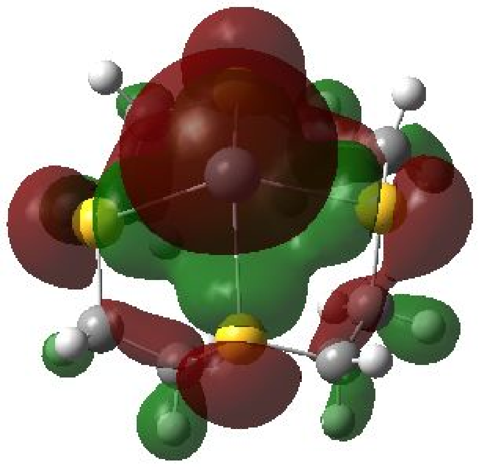	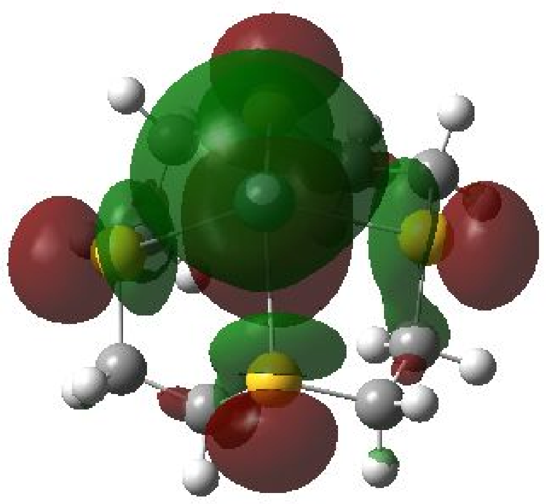
**HOMO-1 (-14.47 eV)**	**LUMO+1 (-8.78 eV)**

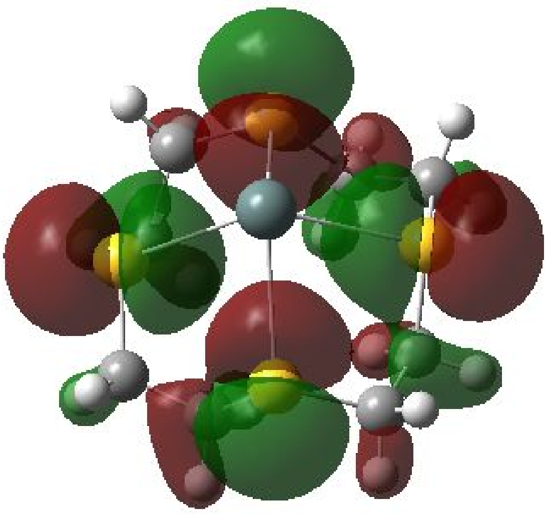	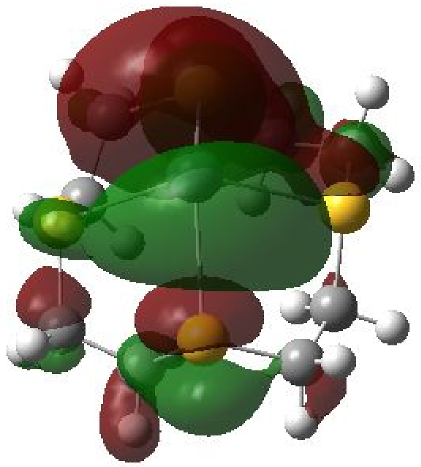
**HOMO-2 (-15.22 eV)**	**LUMO+2 (-8.78 eV)**
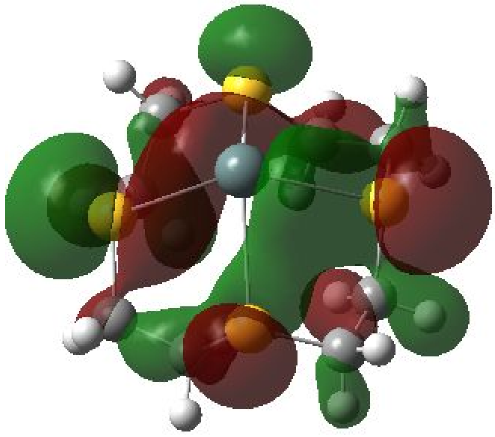	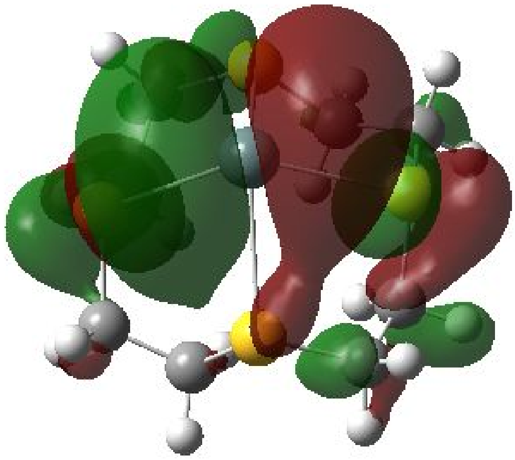
**HOMO-3 (-15.22 eV)**	**LUMO+3 (-9.05 eV)**
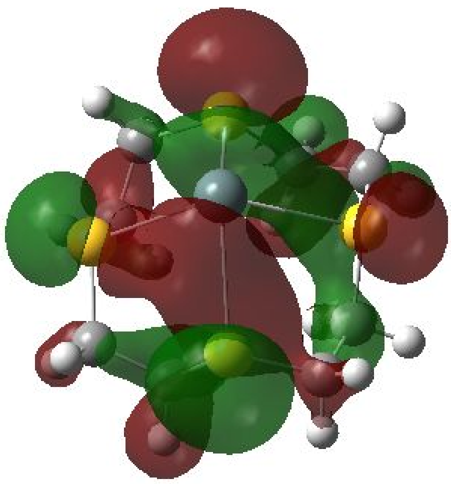	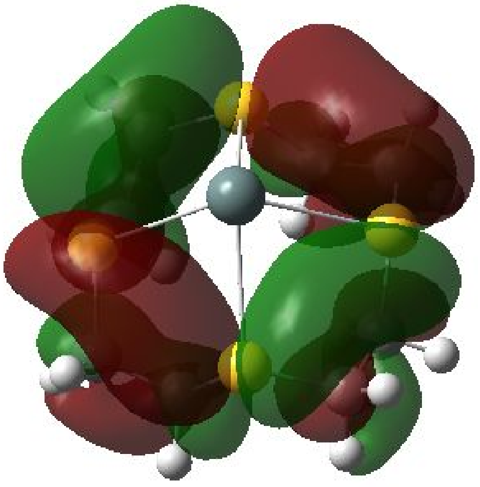

[Pb([12]aneS_4)]$^{2+}$

HOMO (-14.32 eV)	LUMO (-9.02 eV)
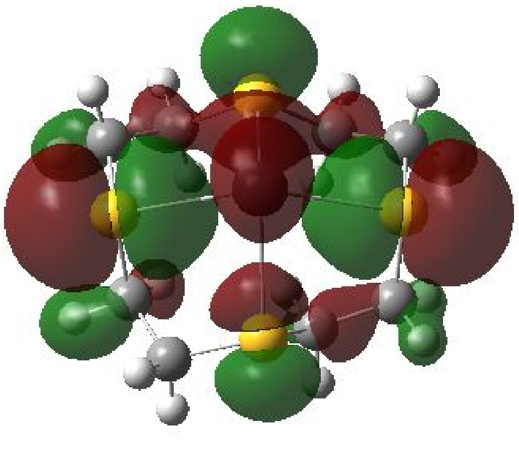	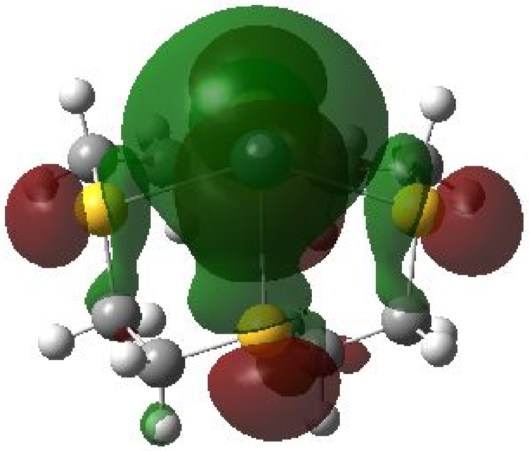
HOMO-1 (-14.80 eV)	LUMO+1 (-8.87 eV)
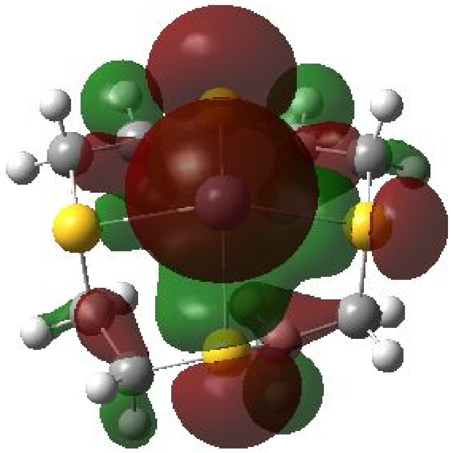	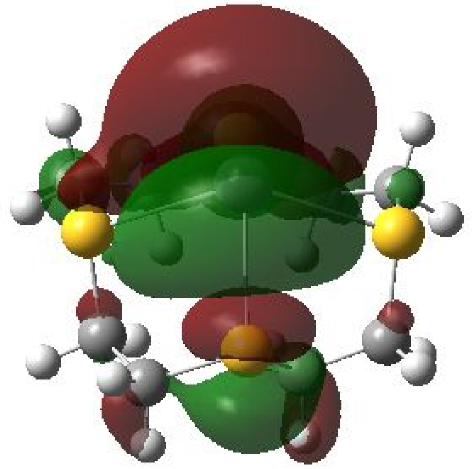
HOMO-2 (-15.02 eV)	LUMO+2 (-8.67 eV)
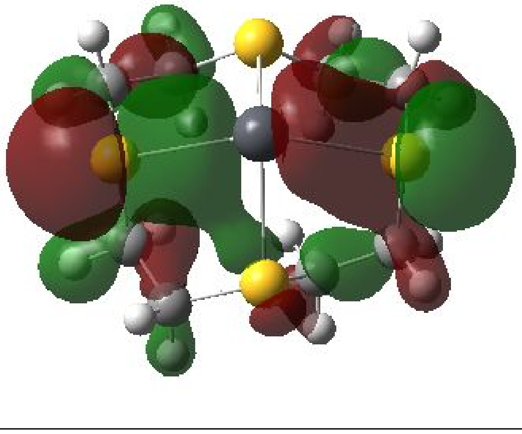	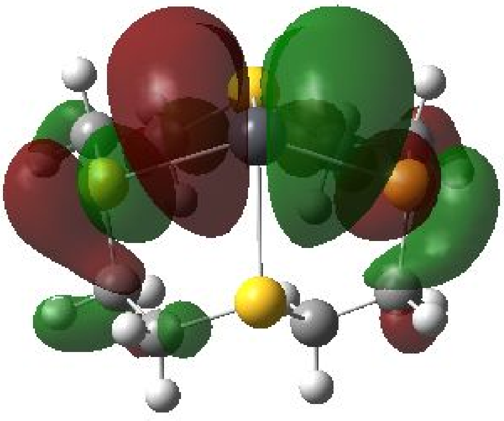
HOMO-3 (-15.06 eV)	LUMO+3 (-7.85 eV)

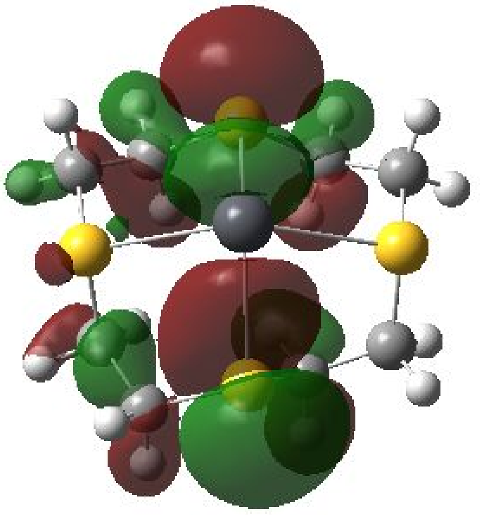	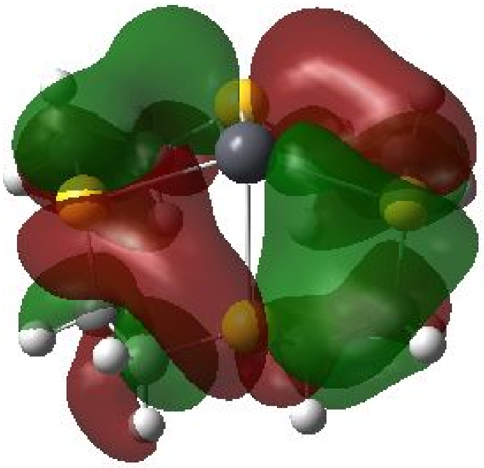

[Pb([24]aneS$_8$]$^{2+}$/[Pb([24]aneS$_8$)(OTf)]$^+$

**LUMO+2 (-6.54 eV)**	**LUMO+2 (-3.10 eV)**
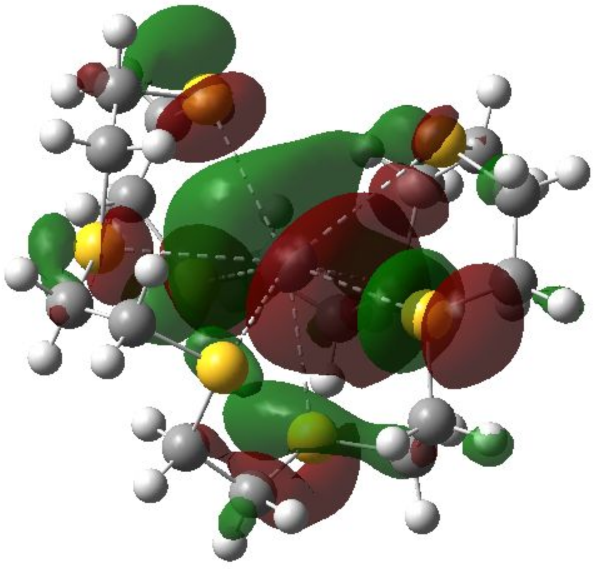	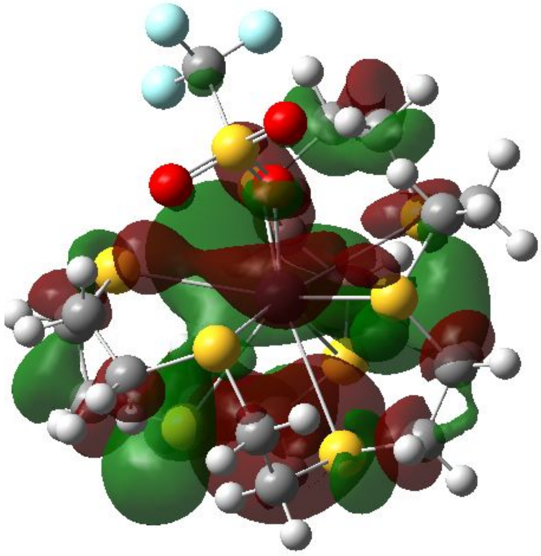
**LUMO+1 (-7.02 eV)**	**LUMO+1 (-3.33 eV)**

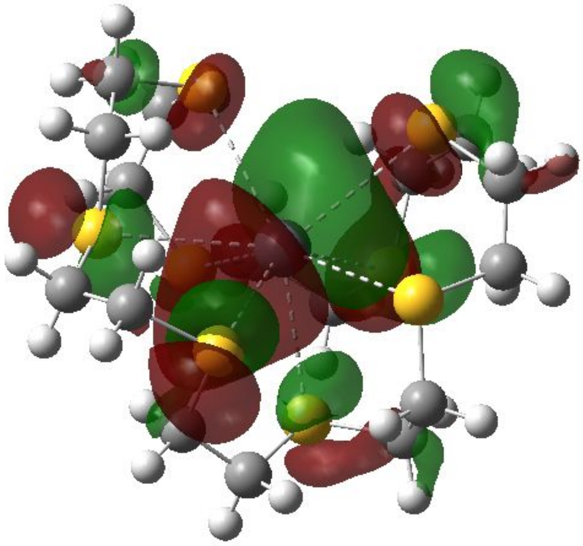	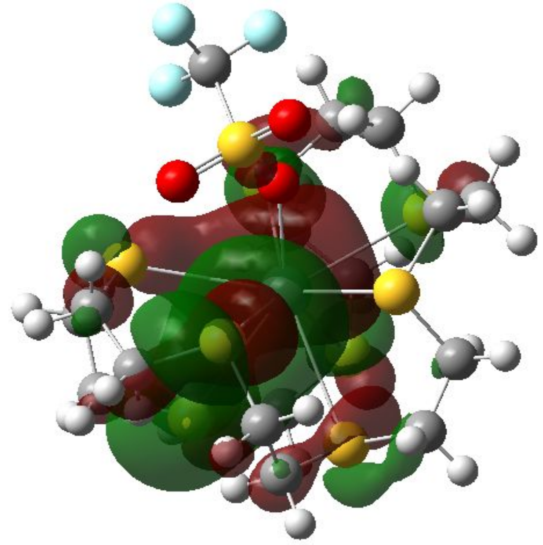
**LUMO (-7.43 eV)**	**LUMO (-3.57 eV)**
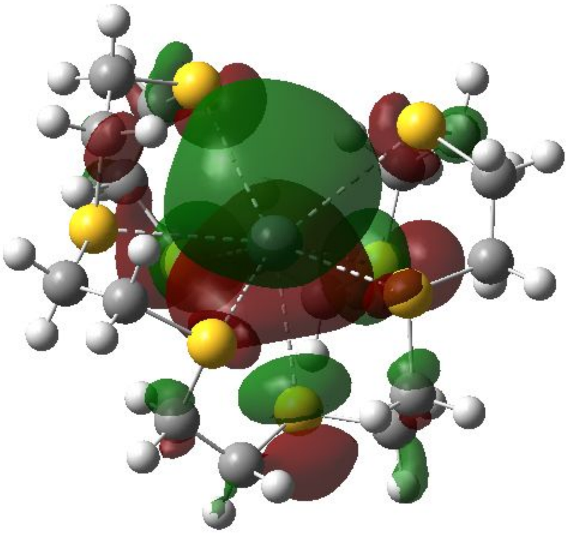	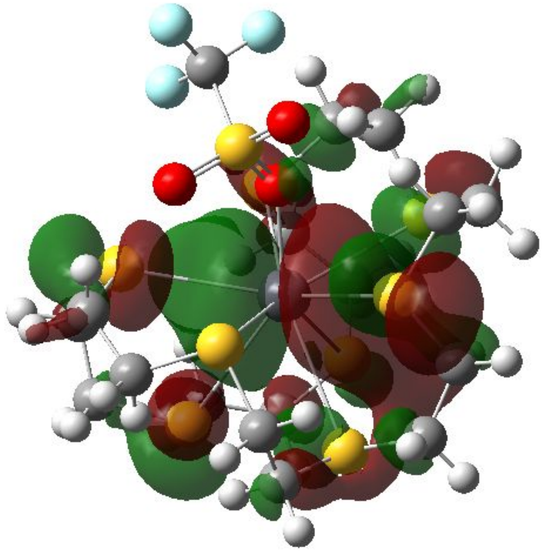
**HOMO (-12.07 eV)**	**HOMO (-8.99 eV)**
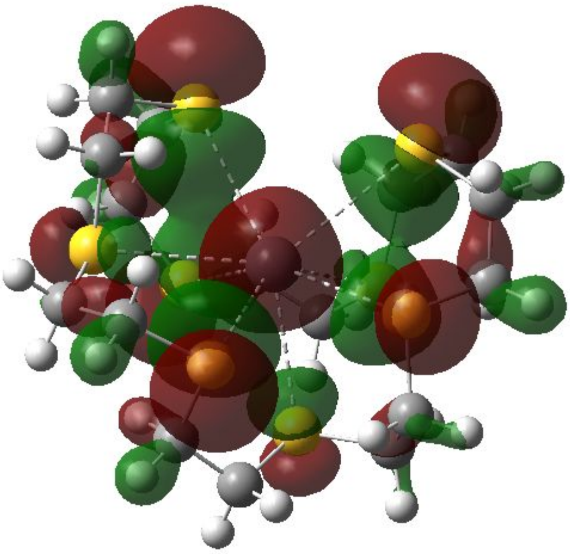	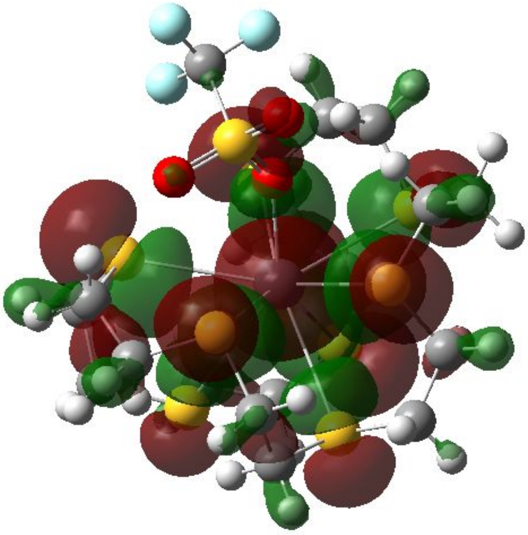
**HOMO-1 (-12.26 eV)**	**HOMO-1 (-9.42 eV)**

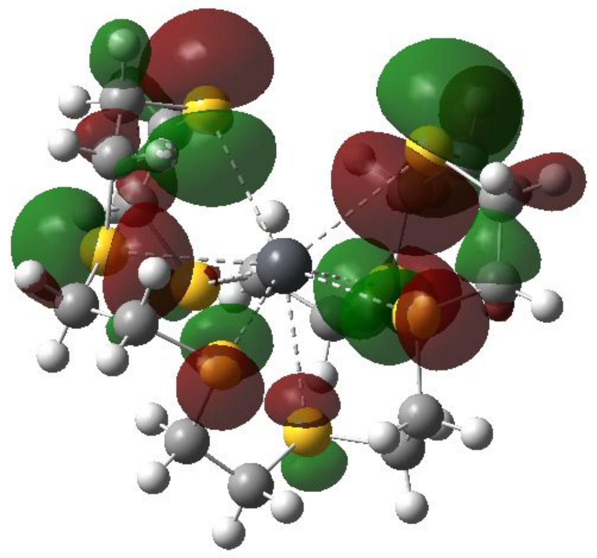	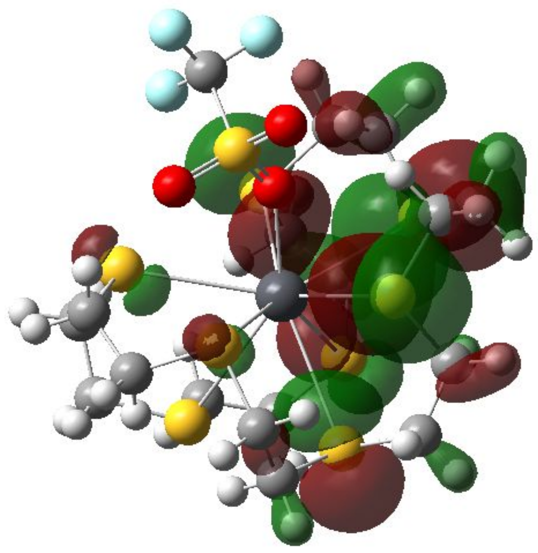
**HOMO-2 (-12.32 eV)**	**HOMO-2 (-9.44 eV)**
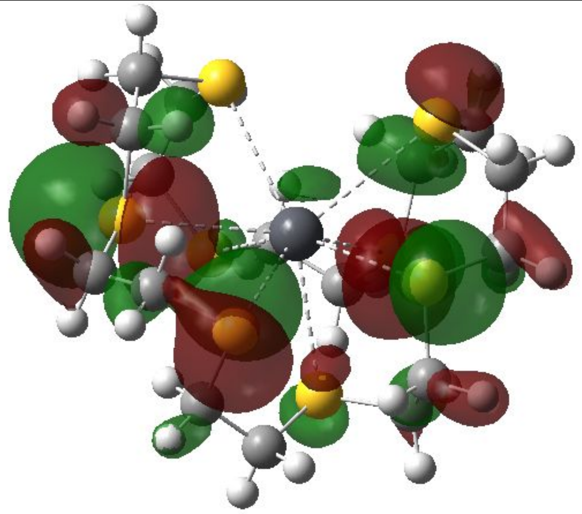	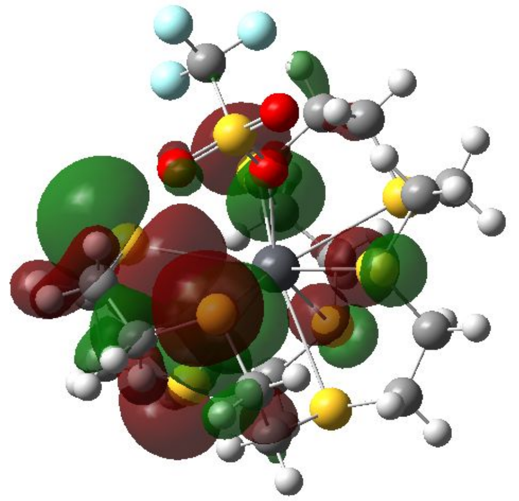

Table S2 Comparison of experimental (X-ray) and computed (DFT) bond lengths (Å) and angles (°) for the divalent group 14 thia-macrocyclic species

$[Ge([9]aneS_3)]^{2+}$	X-ray	DFT
Ge-S	2.4854(7)<br>2.5080(7)<br>2.5450(7)	2.51424<br>2.51408<br>2.51437
S-Ge-S	84.38(2)<br>83.76(2)<br>82.77(2)	86.18039<br>86.18351<br>86.19315

$[Sn([9]aneS_3)]^{2+}$	X-ray	DFT
Sn-S	2.7301(5)<br>2.6996(6)<br>2.7789(6)	2.71360<br>2.71384<br>2.71358
S-Sn-S	76.409(17)<br>78.250(18)<br>77.905(17)	81.01612<br>81.00723<br>81.00890

$[Pb([9]aneS_3)]^{2+}$	X-ray	DFT
Pb-S	2.7831(9)<br>2.8279(10)<br>2.8622(8)	2.76312<br>2.76344<br>2.76336
S-Pb-S	74.16(3)<br>76.20(2)<br>76.13(3)	79.87542<br>79.87227<br>79.87224

$[Ge([12]aneS_4)]^{2+}$	X-ray	DFT
Ge1-S1	2.4446(4)	2.49304
Ge1-S2	2.7338(5)	2.75706
Ge1-S3	2.5050(4)	2.50211
Ge1-S4	2.7717(5)	2.75711
S1-Ge1-S2	75.098(14)	78.16270
S2-Ge1-S3	79.714(14)	82.33145
S3-Ge1-S4	80.724(14)	82.32577
S4-Ge1-S1	74.685(14)	78.16474
S1-Ge1-S3	83.465(14)	89.06879
S2-Ge1-S4	145.525(14)	151.88019

$[Sn([12]aneS_4)]^{2+}$	X-ray	DFT
Sn1-S1	2.9053(9)	2.83855
Sn1-S2	2.8176(8)	2.83838
Sn1-S3	2.8160(8)	2.83864
Sn1-S4	2.8675(8)	2.83873
S1-Sn1-S2	72.30(3)	75.41065
S2-Sn1-S3	74.20(2)	75.40650
S3-Sn1-S4	73.41(2)	75.40151
S4-Sn1-S1	72.65(3)	75,41267
S1-Sn1-S3	115.72(2)	119.73688
S2-Sn1-S4	113.91(3)	119.75115

$[Pb([12]aneS_4)]^{2+}$	X-ray	DFT
Pb-S	3.080(3)	2.90551
	3.070(3)	2.82374
	2.995(3)	2.85784
	3.0315(3)	2.92404
S-Pb-S	67.84(6)	75.45746
	67.85(6)	73.30492
	71.41(6)	74.93315
	68.87(6)	75.19551

$[Pb([24]aneS_8)]^{2+}$	X-ray	DFT $[Pb([24]aneS_8]^{2+}$	DFT $[Pb([24]aneS_8(OTf)]^{+}$
Pb-S	3.1320(9)	3.09634	3.18878
	3.2627(9)	3.20125	3.22010
	3.0889(10)	3.28950	3.03574
	2.9665(9)	3.26591	3.03771
	3.0620(9)	3.35296	3.14015
	3.0674(10)	3.05608	3.36787
	3.1320(9)	3.06861	3.31053
	3.1770(9)	3.10245	3.23048
Pb-O	2.645(3)	-	2.29479
